# Uncovering Statistical Links Between Gene Expression and Structural Connectivity Patterns in the Mouse Brain

**DOI:** 10.1007/s12021-021-09511-0

**Published:** 2021-03-11

**Authors:** Nestor Timonidis, Alberto Llera, Paul H. E. Tiesinga

**Affiliations:** 1 Neuroinformatics department, Donders Centre for Neuroscience, Radboud University Nijmegen, Heyendaalseweg 135, 6525AJ Nijmegen, The Netherlands; 2 Department of Cognitive Neuroscience, Radboud University Medical Centre, Kapittelweg 29, 6525EN Nijmegen, The Netherlands; 3 Centre for Cognitive Neuroimaging, Donders Institute for Brain, Cognition and Behavior, Kapittelweg 29, 6525EN Nijmegen, The Netherlands

**Keywords:** Linked ICA, Axonal projection density, Gene expression, Bayesian machine learning, Matrix factorisation, Dictionary learning and sparse coding, Mouse brain mesoconnectome, Spatial transcriptomics, Connectomics, Computational framework, Tract-tracing, In situ hybridization, Volumetric brain representation

## Abstract

Finding links between genes and structural connectivity is of the utmost importance for unravelling the underlying mechanism of the brain connectome. In this study we identify links between the gene expression and the axonal projection density in the mouse brain, by applying a modified version of the Linked ICA method to volumetric data from the Allen Institute for Brain Science for identifying independent sources of information that link both modalities at the voxel level. We performed separate analyses on sets of projections from the visual cortex, the caudoputamen and the midbrain reticular nucleus, and we determined those brain areas, injections and genes that were most involved in independent components that link both gene expression and projection density data, while we validated their biological context through enrichment analysis. We identified representative and literature-validated cortico-midbrain and cortico-striatal projections, whose gene subsets were enriched with annotations for neuronal and synaptic function and related developmental and metabolic processes. The results were highly reproducible when including all available projections, as well as consistent with factorisations obtained using the Dictionary Learning and Sparse Coding technique. Hence, Linked ICA yielded reproducible independent components that were preserved under increasing data variance. Taken together, we have developed and validated a novel paradigm for linking gene expression and structural projection patterns in the mouse mesoconnectome, which can power future studies aiming to relate genes to brain function.

**Supplementary Information:**

The online version contains supplementary material available at doi: 10.1007/s12021-021-09511-0.

## Introduction

Bridging the gap between genes and brain structural connectivity is of the utmost importance to make further progress in neuroscience. One important reason for doing so is to unravel the internal wiring diagram of the brain often referred to as the connectome, given its generation by specific spatiotemporal patterns of gene expression during development and its fine-tuning by neural activity beyond that period (Kang et al. 2011; Henry and Hohmann 2012). Hence, gene expression has been suggested to explain aspects of the connectome that can not be fully explained by its spatial constraints, such as heritability, optimizing network-cost efficiency and the overdispersion of projections (Smit et al. 2008; Glahn et al. 2010; Fornito et al. 2011; van den Heuvel et al. 2013; Gǎmǎnuţ et al. 2018; Wang 2020b). Additionally, finding correlations between gene expression patterns and changes in endophenotypes such as cortical thickness has been used to understand aspects of neurodegerative diseases, such as autism, Huntington’s disease, schizophrenia and Alzheimer’s disease (Rittman et al. 2016; Romme et al. 2017; Lein et al. 2017b; McColgan et al. 2018; Grothe et al. 2018; Romero-Garcia et al. 2019).

In Sperry (1963), it was first suggested that there are correlations between connected neurons and their transcriptional profiles, which was termed the *chemoaffinity hypothesis*. Since then, classical candidate-variant and GWAS studies have been used to search for relations between genetic variants and interindividual phenotypical variance related to a brain network of interest (Lein et al. 2017a; Luo et al. 2018). As a subsequent step, the emergence of brain-wide gene expression atlases paved the way for new types of hypotheses testing. In particular, the new studies centered around investigating associations between the spatial organization of gene expression and properties related to brain structure or function (Lein et al. 2007; Hawrylycz et al. 2009; Keil et al. 2018). In tandem with this new era of spatial transcriptomics, Roy et al. and Zhu et al. investigated its proteomic counterpart. Specifically, they found postsynaptic protein profiles of excitatory synapses to be markers of synaptic diversity patterns across brain regions that account for different brain networks (Roy et al. 2018; Zhu et al. 2018).

The first studies to establish structural links between gene expression and connectivity were done for the Caenorhabditis elegans (C. elegans) species, by applying computational models to predict synaptic connections between neurons using their gene expression profiles (Kaufman et al. 2006; Baruch et al. 2008). Afterwards, multiple studies focused on the rodent brain and applied statistical and computational analyses finding relationships between gene expression and structural connectivity at the mesoscale level (French and Pavlidis 2011a, 2011b; Wolf et al. 2011; Rubinov et al. 2015; Fulcher and Fornito 2016). The common denominator between these studies was the finding of significant correlations across brain areas between network properties of the mesoconnectome, such as the number and strength of ingoing and outgoing connections, and correlated gene expression (CGE) patterns.

Fakhry et al. (2015) applied the partial Mantel test to find relationships between gene expression and the projection target specificity of different source brain areas. This was the first analysis to be done on a volumetric level instead of using a graph representation of the connectome-based network properties, thus retaining a level of information that was closer to the original experimental data than before.

However, the partial Mantel test faces a number of limitations. First, it computes the correlation between multiple distance matrices, with the pairwise distance being taken across a shared dimension (Castellano and Balletto 2002). In Fakhry et al. (2015), the shared dimension was at the level of brain areas and the original matrices used for estimating the distance matrices were the projection density, injection density and gene expression datasets, respectively.

Given that genes do not correspond to the shared dimension, the effects of genes on connectivity patterns can only be accounted in a consequent analysis. Second, a consequent gene ranking strategy does not highlight modules of gene co-expression, modules of heavily interconnected areas and interactions between the two types of modules, whose importance in brain structures and function have been highlighted in multiple studies and can serve as a dimensionality reduction strategy (Langfelder and Horvath 2008; Grange et al. 2014; Li et al. 2017; Kobak et al. 2019).

In this study we simultaneously identify links between the gene expression and the axonal projection density in the mouse brain, using volumetric data from the Allen Institute for Brain Science and applying a modified version of the Linked ICA method (Groves et al. 2011) to identify independent sources of information that link both modalities at the voxel level. This approach overcomes the limitations of post-hoc correlation strategies by providing multiple implicit linkages between groups of gene expression and projection density patterns, whose functional context can be validated by comparison with literature and ontology enrichment analysis.

## Methods

The aim of this study is the identification of links between volumetric gene expression and axonal projection density data, that were made publicly available by the Allen Institute (Lein et al. 2007; Oh et al. 2014). To uncover such linkage we use a modified version of the Linked ICA method (Groves et al. 2011) to mine for independent components linking the data modalities at the voxel level.

### Data

#### Gene Expression

We retrieved volumetric gene expression data using the Mouse Connectivity Cache (MCC) API provided by the Allen Institute for Brain Science (2). The data are part of the Allen Mouse Brain Atlas (AMBA), in which the expression patterns of $\sim $20.000 genes have been quantified and registered to a 3D space that represents the entire mouse brain at 200 *μm*^3^ resolution.

The experimental setup for quantifying the raw gene expression data is described in Lein et al. (2007), where a combination of the *in situ hybridization* (ISH) and *fluorescence microscopy* techniques was used together with an image processing pipeline. Based on these techniques (Amann and Fuchs 2008), they dissected tissues of 56-days-old (P56) C57BL/6J (wild-type) male mice and generated 25 *μm* thick sections with a 200 *μm* interspacing. These sections were sampled either along the posterior-anterior axis or along the right-left axis, hence labelled as coronal or sagittal sections, respectively. Within each dissected brain slice, they quantified the expression of $\sim $20.000 genes for sagittal sections and 3318 genes for coronal sections by attaching fluorescent RNA probes with complementary sequences to the RNAs of interest and then visualizing the labeling with fluorescence microscopy (Lein et al. 2007).

These coronal and sagittal sections were registered and aligned to a 3D anatomical template based on the Common Coordinate Framework (CCF) version 3.0 of the Allen Institute, which was created by averaging the structural volumes of 1,675 mouse brains (Wang et al. 2020a). The result was 3D volumetric gene expression data of 200 *μm* resolution that was stored in the PIR orientation, where the x-axis corresponds to the Posterior-Anterior axis, y-axis corresponds to the Inferior-Superior axis and z-axis corresponds to the Right-Left axis. We retrieved the expression data from 3318 genes that originated from the coronal slices, given that their in plane resolution was higher than that of the sagittal slices (Lein et al. 2007). From the retrieval options of the MCC API, we chose the expression energy measure of representing the data, which for a given gene in a given voxel can be defined as the summed intensity of expressing pixels divided by the number of all pixels belonging to that voxel.

#### Projection Density

We retrieved volumetric projection density data from the Allen Institute for Brain Science (see Table 2), also with the use of the MCC API. The data are part of the Allen Mouse Brain Connectivity Atlas (AMBCA), in which the axonal projection patterns of 157 brain areas have been quantified and registered to a 3D space that represents the entire mouse brain in multiple scales of resolution.

The experimental setup for quantifying the axonal projection patterns can be found in (Oh et al. 2014), where they processed coronal brain sections of P56 wild-type male mice with a combination of the anterograde tract tracing technique, two-photon microscopy and an image processing pipeline. Specifically, they injected a recombinant adeno-associated virus (rAAV) expressing enhanced green fluorescent protein (EGFP) in the source cortical brain area, which was transported in an anterograde fashion from the injected source areas through the axons to terminate in the target brain areas (Chamberlin et al. 1998; Harris et al. 2012).

This process was visualized with two-photon microscopy and the produced brain volumes were registered and aligned to the CCF v3.0 similarly to the procedure for the gene expression data (see “Gene Expression”). The result was 3D volumes of 498 wild-type tract-tracing experiments that could be retrieved in 10, 25, 50 and 100 *μm*^3^ scales of resolution (Oh et al. 2014).

Regarding data representation options of the MCC API, we chose the projection density measure which for a given tracing experiment in a given voxel can be defined as the sum of detected fluorescent pixels divided by the sum of all pixels that belong to that voxel (see Table 2).

#### Data Preprocessing

Following the data retrieval, we preprocessed the data to an appropriate form for the Linked ICA algorithm. One issue in preprocessing was that the lowest level of resolution for downloading the projection density data was 100 *μm*^3^, which was higher than the 200 *μm*^3^ resolution of the gene expression data. In order to bridge the gap between the modalities, we selected the 100 *μm*^3^ resolution for the projection data and we downsampled them to 200 *μm*^3^ using continuous interpolation (see Supplementary Material 1.13 for the implementation details). This resulted in a 4D array for each modality of 67 voxels in Posterior-Anterior axis × 41 voxels along the Inferior-Superior axis × 58 voxels in Right-Left axis × 498 tract tracing injections (Oh et al. 2014) or 3318 genes (Lein et al. 2007).

Further preprocessing steps were necessary, including filtering out the background voxels outside the brain space and reshaping the 4D arrays into 2D in order for the data to fit into our Linked ICA implementation (see “Linking data modalities”). First, we removed the background voxels by applying a voxel mask that filtered out voxels with the background value of -1. Moreover, we iterated over all genes and injections and we reordered the 3 spatial dimensions into 1 dimension using row-major ordering, one gene at a time. The resulting 2D gene expression and projection density arrays consisted of 63113 voxels × 3318 genes and 63113 voxels × 498 wild-type injections, respectively.

We had to account for the high variance of projection densities, resulting from heterogeneous and hemisphere-asymmetric injections that span the entire cortex and vary in injection volume. Hence, we created projection subsets from the three most densely sampled brain areas, which were the visual cortex (*vis*), the caudoputamen (*cp*) and the midbrain reticular nucleus (*mrn*, see Table 1). The *vis* injection group comprised 47 injections in different areas of the visual cortex, specifically 33 injections in the primary visual area, 4 in the lateral area, 3 in the anteromedial area, 2 in the postrhinal and laterointermediate areas, respectively, and one each in the posteromedial, posterolateral and anterolateral areas, respectively. The *cp* and *mrn* injection groups comprised 19 and 16 injections in the caudoputamen and midbrain reticular nucleus areas, respectively. The three subsets were used in the analysis alongside the entire dataset, as explained in “Evaluation”.

**Table 1:** Injection counts for brain regions with more than 5 injections

	Injection frequency
VISp	33
CP	19
MRN	16
ENTl	10
DG	9
PAG	9
MOs	9
MD	8
ENTm	8
CA3	8
MOp	8
PIR	7
LHA	7
SI	7
SSp-bfd	6
IRN	6
CA1	6
AHN	6
PRNc	6
GRN	6

Abbreviations are listed in supplementary file 1

### Linking data modalities

Linked Independent Component Analysis (Linked ICA) is a bayesian multimodal variation of the classical ICA method (Hyvarinen 1991; Bell and Sejnowski 1995), developed for analysing neuroimaging data (Groves et al. 2011). Linked ICA can be understood as an extension of *tensor ICA* (Beckmann and Smith 2005), where the input data matrices (modalities) share one dimension which is used to link the ICA factorisations of the data modalities. This approach has been shown to outperform other multimodal approaches such as *joint* or *concat ICA* (Calhoun et al. 2006), because it is able to estimate individual modality noise models and number of degrees of freedom, in addition to the estimation of the relative contribution of each modality to each independent source, which allow identifying sources of variation relating to unique data modalities as well as sources shared across modalities (Groves et al. 2011). In terms of implementation, linked ICA uses Variational Bayes. Full details regarding its algorithmic implementation can be found in Groves et al. (2011). Linked ICA has been previously applied to multiple neuroimaging data modalities, such as functional, structural and diffusion MRI, for which the subject dimension has served as the shared element across all modalities (Kincses et al. 2013; Douaud et al. 2014; Itahashi et al. 2015; Wolfers et al. 2017; Llera et al. 2019; Wu et al. 2019; Maglanoc et al. 2020).

In the datasets we consider, all gene expression and projection density patterns have been aggregated from multiple mice (see “Gene Expression”-“Projection Density”). Despite the lack of a shared subject dimension, all data is in fact registered to a common template, namely to CCF v3.0 (Wang et al. 2020a), whose voxels can be used for linkage across modalities. Consequently, we adjusted the original Linked ICA formulation to satisfy the requirements of our datasets and to define a generative model that simultaneously factorises both data modalities matrices $Y^{k} \in M_{R_{k},N}$, with *k* ∈{1,2} being the gene expression or projection density data respectively, *R*_*k*_ the number of genes (*k* = 1) or injection locations (sources) (*k* = 2), and *N* the number of sample brain locations (voxels), as

1 $$ Y^{k} = X^{k} W^{k} H + E^{k}.  $$

$X^{k} \in M_{R_{k},L}$ contains the feature coefficients for each independent component *i* ∈ *L*, *W*^*k*^ ∈ *M*_*L*,*L*_ is a diagonal matrix summarising the *k*^*th*^ modality contribution to each independent component, *H* ∈ *M*_*L*,*N*_ is a matrix shared across modalities that represents the shared spatial source of variation in each independent component, and *E*^*k*^ contains the modality-dependent additive noise. In addition, the independent components are being sorted in descending order based on the total amount of variance that they explain across both data modalities.

The only difference between this formulation and the one originally presented in Groves et al. (2011) is that in our formulation the shared dimension is at the voxel level while in Groves et al. (2011) it was at the subject level. Consequently, although the Bayesian inference process required to learn all the model parameters can be performed as in Groves et al. (2011), the interpretation is different since here we search for independent components of genes and projection densities that are linked through patterns of brain spatial variation.

### Evaluation

Our analysis of the volumetric gene expression and projection density data proceeded in two steps. First we performed 3 different ’local’ analyses by selecting for each analysis only injections in the one of the three most densely sampled brain areas. Specifically, we selected the visual cortex (*vis*), the caudoputamen (*cp*) and the midbrain reticular nucleus (*mrn*, see “Projection Density” for details). Subsequently, we performed a ’global’ linked ICA analysis by including all (498) available injections. For each run of the linked ICA model we monitored convergence using the relative free energy and assessed convergence using a conservative relative change < 10^− 6^. The convergence flag chosen represents a very conservative convergence criterion, and in fact a relative free energy change of order 10^− 6^ represents a change of machine precision order at each model parameter. Setting the parameter to even more conservative (smaller) values does not change the solutions found. This convergence flag was reached for the local analyses but not for the global analysis, due to the maximum number of 3000 iteration steps being exceeded. Instead, the global analysis reached a convergence flag of $\sim 10^{-4}$.

For each of the local analyses we determined a number of components that was equal to the rank of their projection matrices (i.e. number of injections) and for the global analysis we selected 200 components instead of the minimum rank of 498 across the two modalities. We considered it unnecessary to include the additional 298 components in the analysis, since correlation statistics indicated strong links between the lower indexed global analysis components and the local analysis components at the level of spatial maps and coefficients (*H* and *X* matrices respectively, see Eq. 1), as shown in results “Local and Global Independent Components”. In addition, it has been argued in Llera et al. (2019) that it is not necessary to increase model order in a Linked ICA analysis when the correlations of interest have been found in lower indexed components, since they are strongly reproducible at higher order decompositions.

**Fig. 1:**
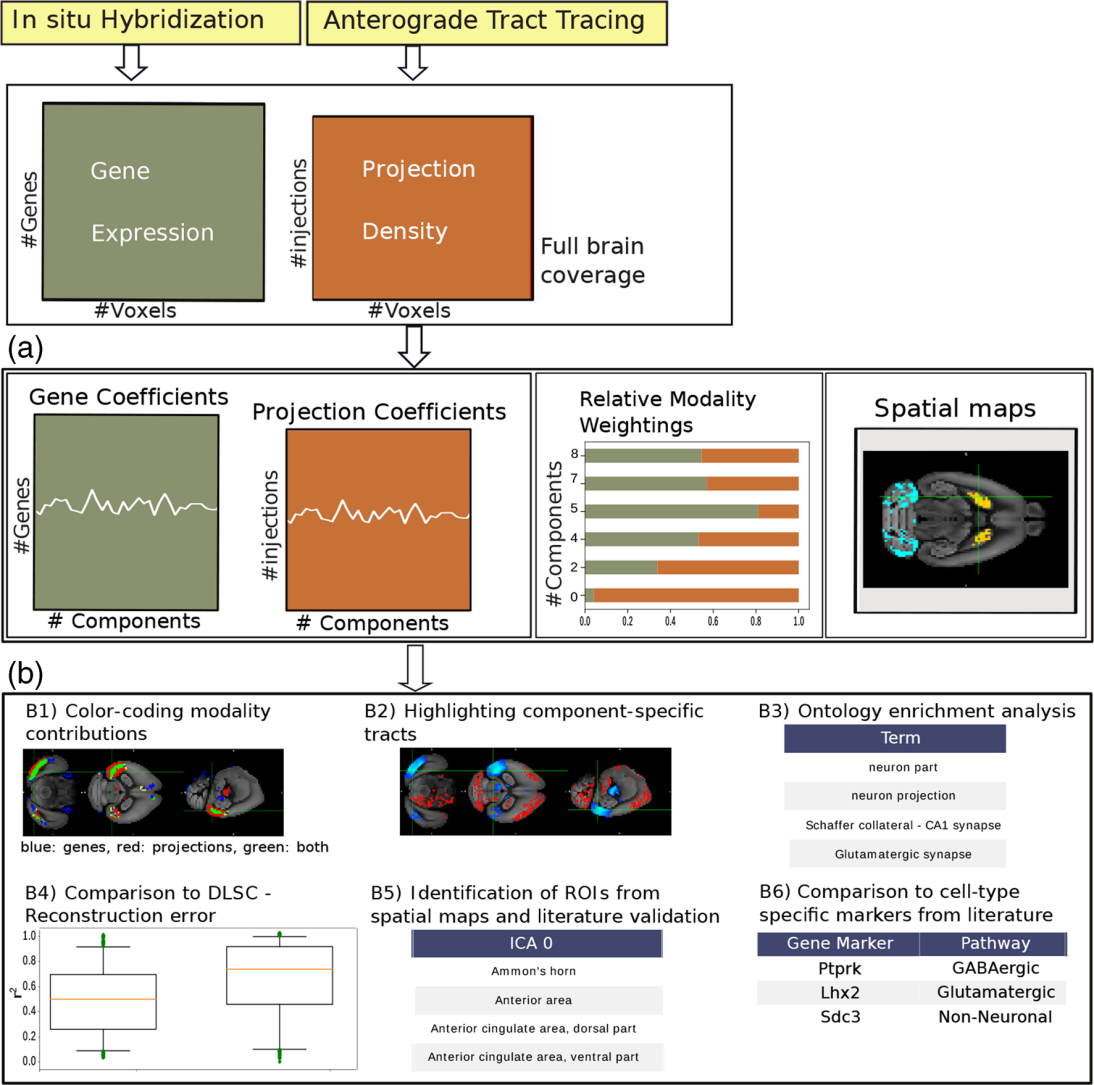
Schematic overview of the Linked ICA analysis workflow. The analysis is based on volumes of the gene expression and projection density modalities, from which we obtain using Linked ICA (**A**) a number of coefficients for genes and projection patterns (*X*^1^ and *X*^2^), the relative modality weightings (*W* ) and a number of spatial maps (*H*). For a more illustrative example in the ’Spatial Maps’ subplot, we presented a visualization of a spatial map instead of a color-coded matrix. Regarding the dimensionality of *H*, rows correspond to independent components and columns correspond to voxels. Hence, the visual example can be interpreted as a row of *H*, reordered in 3D and overlaid with an anatomical template provided by the Allen Institute. **B** We analyse the obtained results and we highlight the contribution of the modalities to the spatial maps using a color-coding scheme (B1), we highlight tracts formed by specific components (B2), we apply ontology enrichment analysis to the gene coefficients in order to find significant functional annotations (B3), we compare the results to the ones obtained from DLSC (B4) and we validate by comparison with literature a number of identified regions of interest from the spatial maps (B5) and a number of cell-type-specific gene markers (B6)

**Fig. 2:**
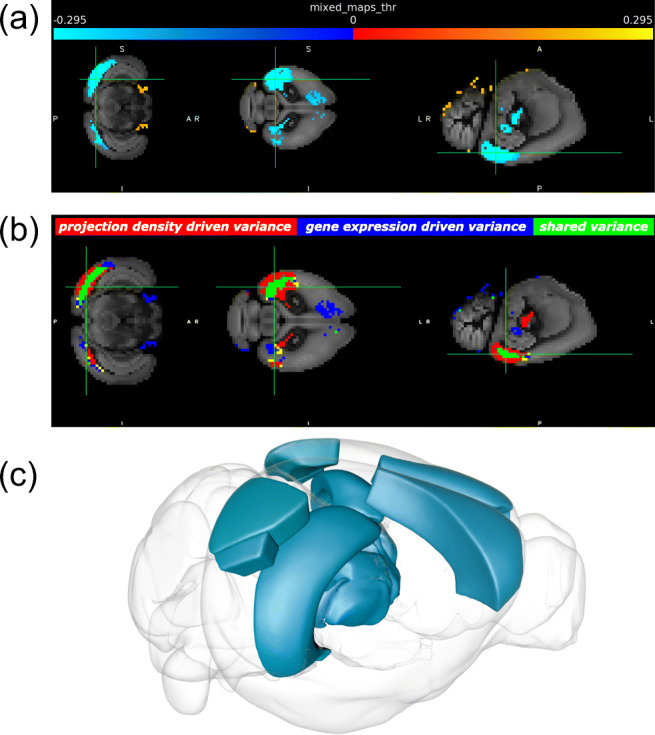
Spatial map visualizations of highlighted brain areas with high variance in component 0 of the *vis* injection group. **a** Shared spatial map, in which the blue-to-lightblue color represents voxels with large negative values (below 1^*st*^-percentile) and the red-to-yellow color represents voxels with large positive values (above 99^*th*^-percentile), see colorbar. A number of highlighted subcortical areas give the impression of being located outside of brain space. This is explained by the low density of the Nissl volume in these areas, which serves as the anatomical template. The template has been plotted overlaid with the spatial map, based on CCF v3.0 (Wang et al. 2020a). **b** Color-coded spatial map used to identify which modality drives the component’s variation in the regions of interest. Green corresponds to voxels with high shared variance, red corresponds to voxels dominated by projection density variance and blue corresponds to voxels dominated by gene expression variance. A detailed description of the color-coding convention can be found in Supplementary Material Section 1.6. **c** Nissl stain volume representation of the highlighted areas using a screen shot from the Scalable Brain Atlas Composer, a 3D brain visualization tool (Bakker et al. 2015) (see Table 2 for more details). Areas with less than 10 voxels of high variance were not visualized with the SBA composer.

**Fig. 3:**
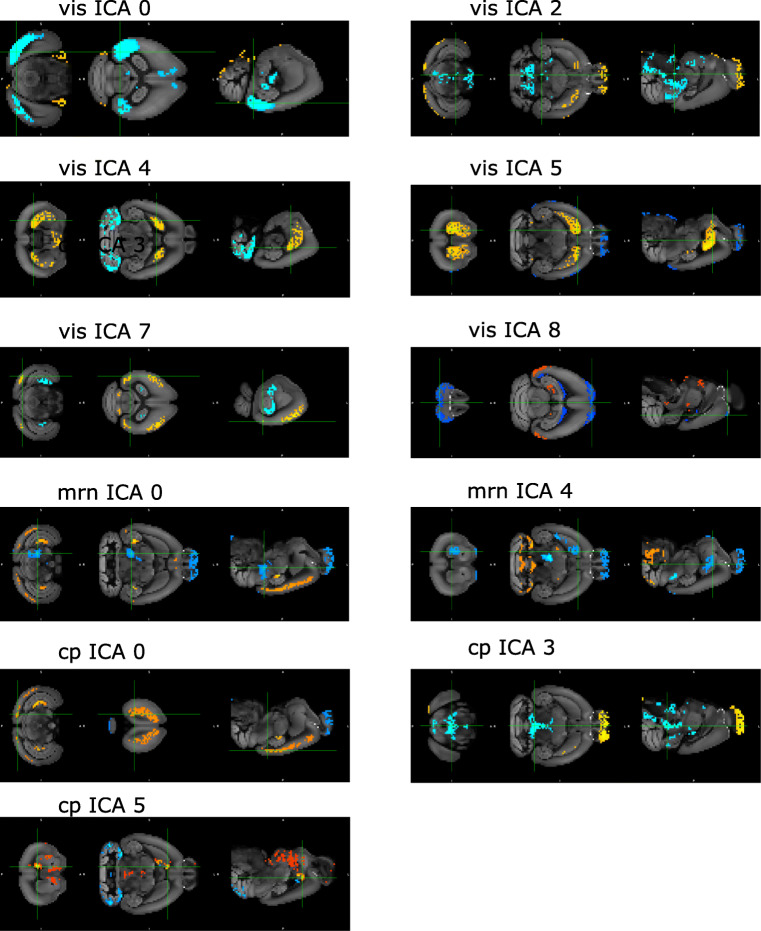
Shared spatial maps for components of interest from the *vis*, *cp* and *mrn* injection groups. The title of each panel identifies the injection group (vis, mrn or cp) and the index of the component. The blue-to-lightblue color represents voxels with large negative values (below 1^*st*^-percentile) and the red-to-yellow color represents voxels with large positive values (above 99^*th*^-percentile), see colorbar in Fig. 2

From the results of each of these 4 analyses we selected as components of interest the ones having a non-zero contribution from both data modalities, meaning that their variance was partly explained by both the gene expression and projection density modality (see Fig. 1 for the relative modality contributions across components). Each component was associated with a brain spatial map that was shared across both modalities and reflected brain areas with shared variance across genes and injections, as well as two weight vectors weighting each modality variable, and a two-dimensional vector reporting the contribution of each modality to the component (*H*, *X* and *W* matrices respectively, see Eq. 1). To aid the interpretation of the results, the spatial maps and the vectors weighting injections and genes were thresholded at percentiles 1 and 99, for each component individually. That is, values within this percentile range were set to zero, while values exceeding that range were considered to highlight the brain areas, genes and injections most involved in each relevant independent component (Figs. 2 and 3). The genes we selected were subjected to enrichment analyses in “Gene Enrichment Analysis”.

In addition, we replicated the analyses by imputing a number of missing values in the gene expression dataset. This subset was originally represented in AMBA using the background value of -1 and comprised 0.8*%* of the total dataset. As an alternative strategy, we imputed each missing value of a given gene to a particular voxel, by assigning the average value of all expressed voxels belonging to the same anatomical subregion, as defined by the Allen annotation volume (see Table 2). This missing value imputation strategy was performed for all genes and was not necessary for the projection density data because it was acquired volumetrically. However, the extracted linked components were not affected by the imputation strategy.

**Table 2:** Hyperlinks for websites, tool descriptions and format descriptions related to our analysis

Linked ICA matlab implementation	https://github.com/allera/Llera_elife_2019_1
Github repository of our Code	https://github.com/ntimonid/Connectomic-Composition-Predictor-CCP-
Allen Institute	https://alleninstitute.org/
CCF v3.0	http://help.brain-map.org/display/mouseconnectivity/Documentation
MCC documentation	https://allensdk.readthedocs.io/en/latest/connectivity.html
AMBA Documentation	https://help.brain-map.org/display/mousebrain/Documentation
AMBCA documentation	https://help.brain-map.org/display/mouseconnectivity/Documentation
FMRIB Software Library	https://fsl.fmrib.ox.ac.uk/fsl/fslwiki
Org.Mm.eg database	http://bioconductor.org/packages/release/data/annotation/html/org.Mm.eg.db.html
KEGG pathway database	https://www.genome.jp/kegg/pathway.html
GOHyperGParams-class	https://www.rdocumentation.org/packages/Category/versions/2.38.0/topics/GOHyperGParams-class
clusterProfiler package	https://www.rdocumentation.org/packages/clusterProfiler/versions/3.0.4/
NIfTI files	https://nifti.nimh.nih.gov/
JSON files	https://en.wikipedia.org/wiki/JSON
Nilearn lbrary	https://nilearn.github.io/
Scikit-learn library	https://scikit-learn.org/stable/
NumPy library	https://numpy.org/
SciPy library	https://github.com/scipy/scipy/
NCBI	https://www.ncbi.nlm.nih.gov/
SBA Composer	https://scalablebrainatlas.incf.org/composer-dev/?template=ABA_v3 (Bakker et al. 2015)

See main text for details

This suggests that the minor percentage of missing values in the gene expression dataset was inadequate to affect any links identified in our analyses.

### Gene Enrichment Analysis

In order to validate the biological context of the selected gene groups, we used gene ontology (GO) and KEGG enrichment analyses (Rice 2007; Rivals et al. 2007). In particular, we represented the selected gene groups with their Entrez id and then searched for annotations belonging to the *KEGG* and *Org.Mm.eg.db* databases (Ogata et al. 1999) that were significantly enriched in these groups (see Table 2). For the GO and KEGG enrichment analysis of each tested gene group, we used the hypergeometric test and we collected significant annotations with a p-value lower than a cutoff of 3.8 × 10^− 5^. This cutoff was calculated by applying the false discovery rate (FDR) to the p-values obtained by the enrichment analysis, in order to correct for all annotations and injection groups under comparison.

See Supplementary Material 1.7 for more details concerning the enrichment analyses, the *KEGG* and *Org.Mm.eg.db* databases and the hypergeometric test, as well as Supplementary Material 1.13 for the software-specific implementations of this form of analysis. Subsequent strategies related to the meta-analysis of the thresholded spatial maps, the modality-specific spatial contributions and the weighted injections and genes, can be found in Supplementary Material 1.6.

### Comparing Linked ICA to DLSC

To compare the results obtained with Linked ICA to alternative decomposition methods, we also considered the *Dictionary Learning and Sparse Coding* (DLSC) method (Mairal et al. 2010; Li et al. 2017) for performing factorisations in the same dimension as in the Linked ICA cases (see Supplementary Material 1.2 for details). To allow for data fusion, we concatenated both data modalities in the voxel dimension and computed the DLSC decomposition of the concatenated matrix, hence creating a fused dictionary set (*concat*-DLSC). This is similar to the *concat-ICA* approach (Calhoun et al. 2006), but by using DLSC instead of ICA. For completeness, we also decomposed the gene expression and projection density datasets independently (i.e. not concatenated), hence creating exclusive dictionary sets of gene expression and projection density (*exclusive*-DLSC). Although these factorizations did not force any linking across modalities, post-hoc statistics were used to extract and quantify relationships between the two independent factorizations and the linked independent components (Li et al. 2017).

We then compared the global Linked ICA and both DLSC approaches (*concat* and *exclusive*) in two different ways. First, we evaluated for each factorization the reconstruction accuracy in terms of *r*^2^. In the context of our work, the *r*^2^ measure can be defined as the fraction of total variance of the original datasets that can be explained by the reconstruction from a factorisation approach (Dodge 2008):

2 $$ r^{2} = 1 - \frac{{\sum}_{i}{(y_{i} - f_{i})^{2}}}{{\sum}_{i}{(y_{i} - \tilde{y})^{2}}},  $$

here the index i represents voxels, y represents the volumetric gene expression or projection density data, $\tilde {y}$ represents its mean and f represents the reconstructed data.

**Fig. 4:**
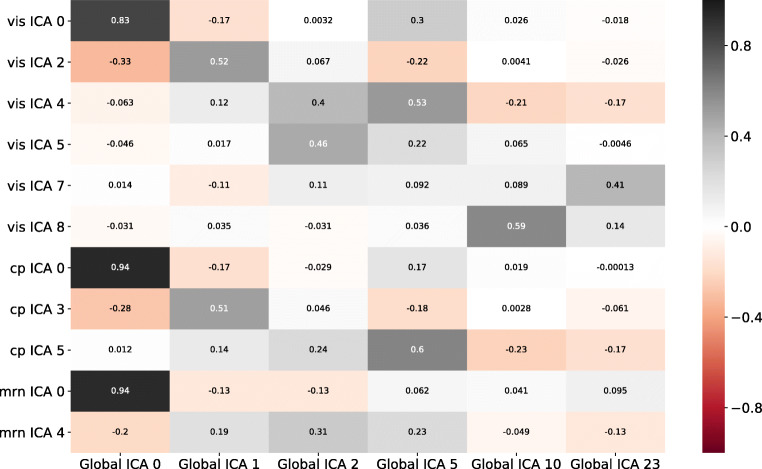
Heatmap displaying the correlation between the local and global components. x-axis: global components 0, 1, 2, 5, 10, 23. y-axis: *vis* components 0, 2, 4, 5, 7 and 8, *cp* components 0, 3 and 5, *mrn* components 0 and 4. The correlation measure used is Pearson’s rho, with the heatmap colors being brighter as the correlation gets higher

Second, we considered the overlap between the different analyses by assessing the significant correlations between the global independent components and the *exclusive* and *concat* dictionaries, estimated by Pearson’s rho, at the level of both spatial maps and coefficients (Fig. 4). We considered significant correlations the ones yielding a p-value lower than 0.004. This is the same threshold as the one used in the comparison between local and global components, as explained in Supplementary Material 1.8. A supplemental evaluation test can be found in Supplementary Material 1.12, where Linked ICA was assessed in reconstructing the spatial patterns of previously unseen genes. Specifically, it yielded a median mean squared error (MSE) of 0.28 with 75 *%* of tested genes having an MSE lower than 1.0.

### Example of a component and its characterization

As a visual summary of the analysis steps described above, Fig. 1 shows a schematic overview of the Linked-ICA and post-hoc analyses performed, and Fig. 2 shows exemplar visualizations of *vis* component 0 in the form of spatial maps, where various brain areas have been highlighted with respect to their shared variance, their modality-specific spatial contribution and their gray-matter volume. The highlighted areas include the primary visual area (417 voxels, 47*%* of intra-area volume), lateral visual area (102 voxels, 66*%* of intra-area volume), secondary motor area (97 voxels, 0.7*%* of intra-area volume), polymodal association cortex of thalamus (13 voxels in total, 7*%* of intra-area volume), ammon’s horn (62 voxels, 0.7*%* of intra-area volume), cochlear nuclei (12 voxels, 0.9*%* of intra-area volume) and ansiform lobule (31 voxels, 0.2*%* of intra-area volume). For *vis* component 0, the highlighted voxels have values of shared variance below the 1^*st*^ or above the 99^*th*^ percentiles (-0.41 and 0.29 z-scored values, see Supplementary Material Tables 4a–7a). Moreover, the spatial map volume of the global analysis was found to have a negative correlation with the Nissl volume (z-scored values, rho = -0.36, p < 10^− 10^). While more brain areas exhibited voxels with high shared variance, such as the laterointermediate area, we selected these areas to report because of their clear visibility in the exemplary visualizations (see Supplementary Material Table 4a for a more extensive report on the highlighted brain areas of *vis* component 0).

**Fig. 5:**
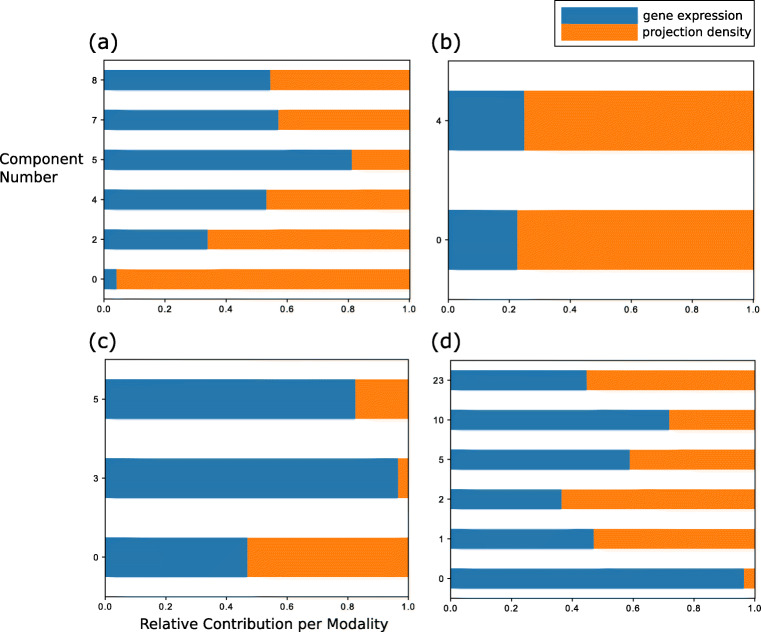
Modality contributions for the components of interest from the *vis*, *mrn*, *cp* injection groups and from the global analysis. **a** *vis* components 0, 2, 4, 5, 7, 8. **b-c** *mrn* components 0, 4 and *cp* components 0, 3, 5. **d** global components 0, 1, 2, 5, 10, 23. x-axis: contribution percentage. y-axis: components (starting from 0). The blue color represents the gene expression data and the orange color represents the projection density data

The variance for the visual areas was mostly driven by the projection density dataset with 215 projection-driven voxels for the primary visual area and 47 projection-driven voxels for the lateral visual area, followed by 140 and 43 voxels with high contribution from both modalities in these areas, respectively. In contrast, the variance of the secondary motor area, and of the subcortical areas were mostly driven by gene expression (88*%* for secondary motor area, 66*%* for ammon’s horn, 75*%* for cochlear nuclei and 83*%* for ansiform lobule, respectively), while the variance of the thalamic module was mostly driven by projection density (100*%*). The modality-specific spatial contributions are reflected in the green, blue and red colors shown in Fig. 2 b. Green corresponds to voxels with high variance contribution from both modalities, red corresponds to voxels dominated by projection density variance and blue corresponds to voxels dominated by gene expression variance (see Supplementary Material Section 1.6 for more details on the color-coding scheme). In addition, the projection density modality accounts for 96*%* of the total variance in *vis* component 0, which can explain its strong contribution to this spatial map (Fig. 5).

Our assumption for the strong projection density dominated variance in the visual areas was that a highly sampled brain region such as the visual cortex will contain numerous dense intra-regional projections that will be explained by the model through its components, based on the fact that source locations tend to project to nearby regions (Oh et al. 2014). Our subsequent assumption for a core of voxels with shared variance in the visual areas, as indicated by the green voxel cluster in Fig. 2, was that the model will explain the variance of gene expression patterns which are present in both a source area and a number of its targets, given that strongly connected areas tend to have highly correlated gene expression patterns (French et al. 2011b; Rubinov et al. 2015; Fulcher and Fornito 2016), hence the gene expression influences the source and target related voxels. As a supplemental assessment regarding the anatomical consistency of the results, readers are suggested to consult Supplementary Material Section 1.9, in which a comparison is made between the global independent components and the parcellation scheme of CCF v3.0.

## Results

### Local and Global Independent Components

We analyzed the results of the *vis*, *mrn* and *cp* injection groups (Fig. 3). The components of interest for the *vis* group were 0, 2, 4, 5, 7 and 8, while for the *mrn* group they were 0 and 4, and for the *cp* group they were 0, 3 and 5. The corresponding components of the global analysis that were significantly correlated with the local components were 0, 1, 2, 4, 5, 10 and 23 (p < 0.004, see Fig. 4 and Table 3). Note that the components are ordered in terms of their total explained variance, hence the number of the component has an independent meaning and is reproducible across different analyses.

**Table 3:** Table containing correlation statistics (Pearson’s rho) between a number of local and global independent components

	Global ICA	Spatial map rho	Gene coefficient rho	Gene coefficient p	Injection coefficient rho	Injection coefficient p
vis ICA 0	0	0.833	0.997	0.0E + 00	0.934	1.1E-21
vis ICA 2	1	0.52	0.876	0.0	0.252	8.8E-02
vis ICA 4	5	0.528	0.8	0.0	0.414	3.8E-03
vis ICA 5	2	0.461	0.577	3.7E-294	-0.129	3.9E-01
vis ICA 7	23	0.407	0.465	7.2E-178	0.5	3.5E-04
vis ICA 8	10	0.588	0.757	0.0	0.293	4.6E-02
cp ICA 0	0	0.94	0.999	0.0	0.95	4.7E-10
cp ICA 3	1	0.507	0.868	0.0	0.467	4.4E-02
cp ICA 5	5	0.603	0.844	0.0	0.251	3.0E-01
mrn ICA 0	0	0.936	0.997	0.0	0.884	5.6E-06
mrn ICA 4	2	0.309	0.799	0.0	0.229	3.9E-01

The global components correspond to the elements of the first column, while the local components correspond to the row indices. The spatial map statistics stem from correlations between the spatial maps of the corresponding components), while the gene coefficient and injection coefficient statistics stem from correlations between the modality-specific coefficients for the same components. The p-values of all spatial map correlations were 0

Each panel of Fig. 3 shows the linked-ICA shared spatial map for an independent component from one of the analyses (see subfigure titles). Each of these maps highlights brain areas having shared variance across modalities (Fig. 5), and Supplementary Material Figs. 3-5 and Tables 4-7 show which genes and injections were relevant for identifying such shared spatial configuration (see Supplementary Material Section 1.6).

Figure 4 shows the similarity of local components to the global ones (see Supplementary Material Section 1.8). When comparing the results between the local analyses and the global one, the correlation values ranged from 0.12 to 0.93 (Table 3). In addition, the respective p-values indicated the significance of most correlations, with only three cases being higher than 0.004 (vis ICA 2 to global ICA 1, vis ICA 5 to global ICA 2, cp ICA 5 to global ICA 5, all on a injection coefficient level). Moreover, all spatial map correlations had a significant p-value below 0.004. Thus, the significant correlations suggest the preservation of the content of local analyses in higher order models, hence establishing some degree of robustness of the approach.

Note that the areas surrounding the injected locations, namely the various visual areas, the midbrain and the dorsal striatum region were highlighted in the spatial maps and tracts, despite the fact that the injection volume was excluded from the projection density dataset. Thus, we assume that the components highlight these locations by capturing the correlated gene expression patterns between the injected locations and their densely projected areas.

The aforementioned brain areas were also highlighted by components that did not belong to the corresponding injection group, for instance *vis* component 2 highlighting the midbrain and *mrn* component 0 highlighting the primary visual area (see Supplementary Material Table 4a and 4b). These correspond to known cortico-striatal and cortico-midbrain connections. Moreover, areas proximal to the injected ones, such as the retrosplenial area for the *vis* group, medulla, pons and hypothalamus for the *cp* group and pallidum for the *mrn* group, were also highlighted.

Direct connections from the primary visual cortex to the dorsal striatum in the mouse brain have been suggested to subserve visually guided motor behaviors and the influence of early visual processing on cortico-striatal synaptic plasticity (Khibnik et al. 2014). Moreover, alterations in cortico-striatal pathways have been shown in rodent model studies to be associated with Huntington’s disease (Cepeda et al. 2007), attention-deficit hyperactivity disorder (ADHD), Tourette syndrome, obsessive compulsive disorder (OCD), autism spectrum disorder (ASD), and schizophrenia (Kuo and Liu 2019). Furthermore, the medial superior colliculus in the midbrain receives connections from multiple cortical areas, including the primary and secondary visual areas, and those connections have been suggested to be involved in avoidance behaviors in mice (Savage et al. 2017). Results related to these cortico-striatal and cortico-midbrain projections in our analysis are represented in *cp* ICAs 0, 3 to the motor, somatosensory and visual areas, *vis* ICAs 2, 4, 5, 7 to the dorsal and ventral striatum, and *vis* ICA 2 to the midbrain (see Fig. 3 and Supplementary Material Table 4).

### Assessing the Biological Context of Selected Gene Groups

The model suggests that the gene subsets which were relevant to the aforementioned components could have a neuronal and synaptic function and might influence various developmental and metabolic processes related to the cortico-striatal and cortico-midbrain pathways in the mouse brain. For the *vis* group, we selected 294 out of 3318 genes (8%), by aggregating all genes that were most involved in each *vis* component of interest (see “Gene Enrichment Analysis” for the selection process). We identified 14 glutamatergic neuronal, 12 gabaergic neuronal and 5 non-neuronal genetic markers (see Supplementary Material Table 3a for the marker gene list), by comparing these 294 genes with the cell-class-specific markers provided by (Tasic et al. 2018b). We also found glutamatergic markers in all *cp* and *mrn* components of interest (Supplementary Material Table 3b). This could explain the uncovering of distal pathways between distinct, spatially separated regions, as long-range projections formed by glutamatergic neurons, in which the identified gene markers are co-expressed.

From the selected genes, LIM motif-containing protein kinase 2 (Limk2) has been shown to be highly expressed in the striatum and assist the migration of cortical interneurons through the subpallium (Cuberos et al. 2015). Another gene selected was syntaxin binding protein 2 (Stxbp2) that belongs to the Syntaxin family, members of which have been suggested to influence the development of Huntington’s disease by interaction alterations in the cortico-striatal pathway (Cepeda et al. 2007). Two additional selected genes were nicotinic acetylcholine receptor subunit alpha-2 (Chrna2), and follistatin-like 1 (Fstl1), which have been shown to be highly expressed in the ventral tegmental area (VTA) of the mouse midbrain (Viereckel et al. 2016). Furthermore, members of the G protein-coupled receptor (Gpr) family were also selected (’Gpr4’, ’Gpr98’, ’Gpr116’, ’Gpr133’, ’Gpr176’, ’Gprc5b’), a member of which has been screened as a VTA marker (Viereckel et al. 2016). Markers Stxbp2 and Fstl1 were also found in the *cp* group, with an additional inclusion being nicotinic acetylcholine receptor subunit alpha-3 (Chrna3) from the same protein family as Chrna2 (see Supplementary Material Table 3a for more details regarding the gene subsets and markers of the *cp* and *mrn* groups).

When performing gene ontology analysis for all components of interest, the major significant annotations from the *Org.Mm.eg.db* database included terms such as postsynaptic specialization membrane, postsynaptic density, synapse part, neuron spine, neuron projection, neurogenesis and neuronal cell body, dendritic tree, axon, plasma membrane bounded cell projection part (p < 3.8 × 10^− 5^), and from the *KEGG* database it included glutamatergic synapse (vis-component 4, p = 0.0003, and cp-component 5, p = 0.0002), phospholipase D signaling pathway (mrn-component 0, p = 0.0002), cholinergic synapse (mrn-component 4, p = 0.0002) and retrograde endocannabinoid signaling (cp-component 5, p = 0.0007). See Supplementary Material Figs. 1 and 2 for further details, as well as Table 4 for comparison of our enrichment results with literature.

**Table 4:** Significant GO and KEGG annotations identified in our analysis that have been cross-linked with previous works

GO/KEGG term	vis ICA	cp ICA	mrn ICA	Literature
synapse	0, 7	0, 3	0, 4	Ji et al. (2014) and Richiardi et al. (2015)
dendrite	7	0	0, 4	Ji et al. (2014)
synapse part	0, 7	0, 3	0	Ji et al. (2014)
generation of neurons	0, 4, 7	0, 3	0	French et al. (2011b)
neuron differentiation	0, 7	0, 3	0	French et al. (2011a, 2011b)
neurogenesis	0, 7	0, 3	0	French et al. (2011a, 2011b), and Richiardi et al. (2015)
neuron projection	0, 7	0	0, 4	French et al. (2011a, 2011b), and Fulcher and Fornito (2016)
neuron development	0	0	0	French et al. (2011a, 2011b)
axon part		0		Ji et al. (2014)
dendritic spine	5		0	Ji et al. (2014) and Fulcher and Fornito (2016)
neuron spine	5		0	Ji et al. (2014) and Fulcher and Fornito (2016)
neuron migration			0	French et al. (2011a, 2011b), and Fulcher and Fornito (2016)
cholinergic synapse			4	French et al. (2011b)
glutamatergic synapse	4	5		French et al. (2011b) and Fulcher and Fornito (2016)

### Comparison with Dictionary Learning and Sparse Coding

We compared the global independent components with the *concat* and *exclusive* dictionaries that were obtained from the *Dictionary Learning and Sparse Coding* (DLSC) method. We made two different comparisons of the two methods. First, we calculated their reconstruction accuracy, followed by estimating the Pearson’s correlation coefficient (rho) between the shared spatial maps and a number of *concat* and *exclusive* dictionaries (see “?? ?? ”).

The reconstruction accuracy was assessed using the *r*^2^ measure, as shown in Table 5a. The performance of *exclusive*-DLSC was slightly better than Linked-ICA for the projection density modality (*r*^2^ = 0.67 and 0.64 respectively), and slightly worse for the gene expression modality (*r*^2^ = 0.66 and 0.67 respectively). Meanwhile, *concat*-DLSC had lower *r*^2^ values. The results were qualitatively similar when using the MSE as a measure. Taken together, this suggests that the different methods capture a similar amount of variance.

Regarding their pairwise correlation, Supplementary Material Tables 5b-d display significant correlations (p < 0.004) between components of interest and *exclusive* or *concat* dictionaries, respectively, at the level of both coefficients and spatial maps. Moreover, as shown in Table 5d, correlations of coefficients between components and *concat* dictionaries followed the relative modality contributions of the components, such as in component 0 where gene-coefficient correlations were higher than the injection-coefficient ones (0.78 versus 0.44), and the gene expression relative contribution was also higher than the projection density one (0.96 versus 0.03).

**Table 5:** Tables containing statistical comparisons between the Linked ICA and DLSC results

(a)						
	*exclusive*-DLSC	*concat*-DLSC	linked-ICA			
*r*^2^ gene	0.67	0.65	0.68			
*r*^2^ projection	0.69	0.45	0.64			
(b)						
	Gene Dictionary	A1 rho	A1 p	A2 rho	A2 p	
global ICA 0	9	0.447	0.0	0.779	0.0	
global ICA 1	5	0.334	0.0	0.584	0.0	
global ICA 2	22	0.265	0.0	0.473	0.0	
global ICA 4	2	0.170	0.0	0.697	0.0	
global ICA 5	2	0.349	0.0	0.803	0.0	
global ICA 10	187	0.321	0.0	0.401	0.0	
global ICA 23	7	0.258	0.0	0.431	0.0	
(c)						
	Projection Dictionary	B1 rho	B1 p	B2 rho	B2 p	
global ICA 0	47	0.030	0.0	0.578	0.0	
global ICA 1	166	0.470	0.0	0.336	0.0	
global ICA 2	2	0.455	0.0	0.558	0.0	
global ICA 4	3	0.444	0.0	0.636	0.0	
global ICA 5	2	0.206	0.0	0.598	0.0	
global ICA 10	195	0.187	0.0	0.412	0.0	
global ICA 23	197	0.381	0.0	0.369	0.0	
(d)						
	*Concat* Dictionary	C1 rho	C1 p	C2 rho	C3 rho	C4
global ICA 0	182	0.285	0.0	0.787	0.447	0.964
global ICA 1	47	0.449	0.0	0.422	0.713	0.470
global ICA 2	10	0.362	0.0	0.381	0.867	0.364
global ICA 4	129	0.412	0.0	0.356	0.694	0.165
global ICA 5	43	0.249	0.0	0.689	0.485	0.588
global ICA 10	92	0.250	0.0	0.439	0.276	0.718
global ICA 23	4	0.326	0.0	0.468	0.571	0.447

a) reconstruction error statistics (*r*^2^) across the analyses of global linked-ICA, *exclusive*-DLSC and *concat*-DLSC, for both the gene expression and projection density modalities. b,c) correlation statistics (Pearson’s rho) between the global components of interest and gene and projection exclusive dictionaries, labeled as A and B respectively. A1,A2,B1,B2: correlations between spatial maps (A1,B1), gene coefficients (A2) and injection coefficients (B2). d) correlation statistics between the global components of interest and the *concat* dictionaries. C1, C2, C3: correlation between spatial maps (C1), gene coefficients (C2) and injection coefficients (C3). C4: the relative contribution of the gene expression modality to the component of interest. For all comparisons, p < 0.004

**Fig. 6:**
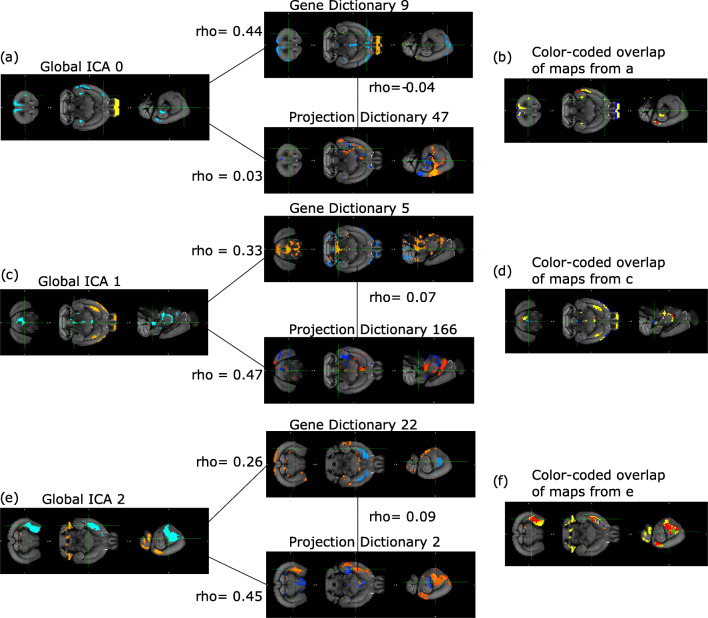
A visual comparison of the spatial maps derived from Linked ICA and *exclusive*-DLSC. **a-f** spatial map visualizations of independent components, gene dictionaries and projection dictionaries, which are followed by their color-coded overlap maps. **a-b** component 0, gene dictionary 9 and projection dictionary 47. **c-d** component 1, gene dictionary 5 and projection dictionary 166. (e-f) component 2, gene dictionary 22 and projection dictionary 2. The spatial maps from (a,c,e) are related through lines with their Pearson’s rho correlation value. The color-coding in maps (b,d,f) is used to identify which modality drives the component’s variation in the regions of interest that were selected by thresholding and masking the spatial maps (below 1^*st*^-percentile and above 99^*th*^-percentile, see Supplementary Material Section 1.6 for more details). The color convention for the spatial map visualizations is similar to the one used in Fig. 2 and a detailed description of the color-coding convention can be found in Supplementary Material Section 1.6

The presence of significant correlations between components of interest, gene dictionaries and projection dictionaries was evident when visually comparing their spatial maps (see Figure 6). As shown by their color-coded maps, most of the areas covaried either between components and gene dictionaries or components and projection dictionaries (highlighted by the blue and red colors in Fig. 6 b, d, f). Examples include the primary visual area (components 0 and 2, gene dictionary 2 and projection dictionary 47), anterior cingulate area (component 0 and gene dictionary 9), secondary motor area (component 1, gene dictionary 5), hypothalamus (component 1, projection dictionary 166) and dorsal striatum region (component 2, gene dictionary 22 and projection density 2). For a more extensive comparison between the two methods, see Supplementary Material Section 1.5.

## Discussion

In this work we searched for links between gene expression and axonal projection densities in the mouse brain. Specifically, we used a modified version of the Linked ICA method (Groves et al. 2011) to link volumetric gene expression and axonal projection data, which were provided by the Allen Institute for Brain Science. Specifically, we identified independent components that account for shared spatial variance across both data modalities.

Initially, we created projection subsets from the three most densely sampled brain areas, namely the visual cortex, midbrain reticular nucleus and caudoputamen injection groups (see “Projection Density”). For each group, we performed a local analysis and we identified independent components whose spatial patterns exhibited high shared variance in brain areas related to the injected location (source) and long-range projections. These results were validated by literature, including known cortico-midbrain and cortico-striatal projections as well as intra-connections within the cortex, brainstem and subcortical nuclei. Moreover, the results were highly preserved when including the complete dataset of 498 injections in the analysis, hence indicating the capability of Linked ICA to preserve independent components under increasing data variance and size (see “Local and Global Independent Components”, Table 3). The validity of these results was enhanced by consistency with previous studies and the well-established *Org.Mm.eg.db* and *KEGG* databases (see Table 2). This consistency was related to a number of detected white-matter tracts and to identified gene groups with functional annotations relative to neurotransmitter-relevant pathways, neuronal function and cell-type specific markers (Tasic et al. 2016, 2018a).

To our knowledge, this is the first study that identified data-driven links between volumetric gene expression and projection density in the mouse brain, instead of links between simplified graph representations at the level of brain areas. Thus, this work expands spatial transcriptomic-based and connectomic-based analyses to high-dimensional data.

The reason to compare the local analyses with the global one is the reduced connectivity sample sizes available when performing the local analyses with respect to the obviously bigger sample size of the global analysis. Note that the gene expression sample size is constant since it is fixed across all analyses. While the advantage of a higher sample size is clear, including different injected areas strongly increases the connectivity variance. Therefore, the increased sample size might provide less specific results. Since it is not absolutely clear which approach is the optimal one, we decided to explore both and we found that the spatial maps of the local analyses were significantly reproduced in the global analysis.

As an additional validation, we compared the components from Linked ICA with dictionaries from the DLSC technique, which explained exclusive variance from each data modality (*exclusive*-DLSC) and shared variance (*concat*-DLSC). We observed that a pairwise correlation between the spatial maps and the coefficients of both approaches revealed significant links between components and dictionaries that indicated high variance in the same brain regions. Therefore, these patterns of shared spatial variance were captured by multiple decomposition methods. A comparison of their reconstruction accuracy revealed that Linked ICA was superior to *concat*-DLSC but slightly inferior to *exclusive*-DLSC. Hence, Linked ICA was more optimal in data fusion instead of reconstruction which is reasonable given that it focuses on explaining variance of multiple modalities (see “Comparison with Dictionary Learning and Sparse Coding”).

These findings suggest that relating both types of dictionaries using pair-wise correlations is not a trivial issue, since a gene dictionary might be more accurately represented as a mixture of projection dictionaries and vice versa. This points out the necessity of conducting post-hoc regression analyses for identifying the most optimal mixtures of dictionaries. In Ji et al. (2014) and Timonidis et al. (2020), predictions of projection patterns as sparse linear combinations of gene expression patterns were shown to be significant when representing both modalities at the level of brain areas. However, Linked ICA provides an advantage in terms of interpretation, since reconstructing both data modalities is implicitly modelled by the method instead of requiring post-hoc analyses.

We acknowledge some limitations. Unlike the Diffusion Tensor Imaging technique that uses seeds to directly represent source locations (Le Bihan and Breton 1985), the injected locations were indirectly represented by the feature space of the projection matrix. This resulted in difficulties to find connections between the identified components and axonal pathways. For augmenting the source representation, incorporating single-neuron morphological data could shed light on projection motifs that have not been covered by tract tracing data, as shown in (Han et al. 2018). An exemplary repository was made available by the *Mouselight* project, where they have provided reconstructions of long-range projections from $\sim $1000 individual neurons in the mouse brain (Gerfen et al. 2016; Hooks et al. 2018; Economo et al. 2018, 2019; Winnubst et al.^2019^). Such data could be fused together with the bulk tracing data and the gene expression data using Linked ICA, with the resulting components linking genes to previously unidentified projection motifs. A preliminary evaluation of this strategy can be found in Supplementary Material Section 1.10, where we have linked single-neuron morphology data with the other two modalities using Linked ICA. We show that the resulting spatial patterns highlight brain regions shown in previous studies (Winnubst et al. 2019), and that they can be used to complement tract-tracing data from less sampled brain regions in the Allen Mouse Brain Connectivity Atlas, such as the motor cortex.

Second, the cell-type specificity of components was evaluated through ontology enrichment analysis and comparison with literature. Note that we could not present direct evidence of cell-type specificity, since the 200 *μm*^3^ spatial resolution of the data is insufficient to resolve the cellular-level. The relation to cytoarchitecture is important, since it has been shown in literature that connected brain areas have similar synaptic and protein profiles (Sperry 1963; Roy et al. 2018; Zhu et al. 2018). Therefore, relating cell-type-specific densities or expression patterns to connectome-based data is crucial for understanding the causative factors that link molecules to brain structure. A pivotal step would be to incorporate single-cell RNA sequencing data with the use of tools such as *SEURAT* (Satija et al. 2015) for identifying cell-types with less bias and imputing missing data that were caused by the 200 *μm* thick sections of the original ISH volumes along the posterior-anterior axis (Lein et al. 2007). Important single-cell RNA-seq sources can be found in Tasic et al. (2016, 2018b), and Mancarci et al. (2017).

A question that can arise is whether the observed links can be attributed to spatial autocorrelation, meaning an increased connection likelihood and correlated gene expression between nearby brain regions. It is well known that spatial gene expression patterns have a strong spatial autocorrelation that reflects the mouse brain cytoarchitecture (French et al. 2011a, 2011b). Previous studies have shown that highly correlated gene expression patterns exhibit both strong global spatial autocorrelation and spatially overlap with connectivity networks (Richiardi et al. 2015; Pantazatos and Schmidt 2020). Linked ICA automatically estimates spatial degrees of freedom that are included in the cost function (Groves et al. 2011), Therefore, spatial autocorrelation is carried downstream by our analysis, since there is no explicit correction for this in output spatial maps. However, previous studies have identified significant correlations between connectivity and gene expression, when correcting for spatial correlation by regressing correlations on the distance and assessing the significance of the residuals (French and Pavlidis 2011a). Thus, we acknowledge that there might be relevant statistical links between structural connectivity and gene expression beyond spatial autocorrelation that need further characterization (Fulcher and Fornito 2016; Fornito et al. 2019). This could be exemplified by components exhibiting high variance in brain areas distal to each other and the injected region, with a relatively balanced contribution between both modalities suggesting a strong linkage beyond spatial autocorrelation. Exemplar cases include *vis* ICAs 4,7,8 and *cp* ICA 0 (see Fig. 5a-c for the modality contributions and Fig. 3 for the spatial maps). For instance, the major highlighted areas in *vis* ICA 4 were striatum, both dorsal and ventral regions, and cerebellum-related fiber tracts (see Supplementary Material Table 4), which can not be fully attributed to spatial proximity. Moreover, the identification of glutamatergic markers in the gene modules of these components (see Supplementary Material Table 3) could explain the presence of long-range projection patterns between the distal areas, given that glutamatergic neurons from one area are known to project to different brain areas (Tasic et al. 2018b). Determining the underlying cellular subtypes of these markers could shed light on the regional and layer-specific projection preferences of these areas. Consequently, links between connectivity and genes might be more localized than it was presented in this work; the 200 *μm* resolution at the source level smoothes out cell-type-specific patterns and their potential links, hence retrieving data at 25 *μm* or higher could resolve this issue (Cheveé et al. 2018; Han et al. 2018; Huang et al. 2020; Kim et al. 2020).

A follow-up question to the spatial autocorrelation issue is whether the underlying causal factors connecting both modalities can be uncovered through linked ICA. The patterns that are linked are spatial patterns, hence the density of a particular gene can be expressed as a pattern across regions, which matches the patterning of the fluorescent labels of the connectivity data. Thus, this can be an epiphenomenon. There are two approaches for validating the causality of the link. First, via a gene ontology analysis that establishes a functional role of the involved genes in generating the projection, for instance, by being expressed in the subset of neurons that make up the projection. For that reason, integrating single-cell RNA seq data is useful. Second, via experimental manipulation of the identified genes (Polleux 2005; Miller et al. 2010; De la Rossa et al. 2013; Daimon et al. 2015; Razoux et al. 2017; Goodman and Bonni 2019). The goal of the paper is to provide a toolbox that can generate such hypotheses and be used to formulate experimental studies to validate them. It has to be borne in mind that development processes generating the projections have finished by the time we quantify the gene expression patterns (French and Pavlidis 2011a), so a direct link is difficult and must rely on turn-over of molecules at the synapse.

While the main focus of this study was to find links between genes and projection patterns on the mouse mesoconnectome, we aim to go beyond qualitative descriptions of such links and move towards more into quantitative tests. We intend to do that by manipulating the expression of genes of interest according to their functional gene modules and then predicting the brain-wide changes in projection densities. Hence, this would make it possible to test in silico a number of neurodegenerative disease-related hypotheses. For two preliminary test cases highlighting the predictive capabilities of Linked ICA, readers are referred to Supplementary Material Section 1.11 in which controlled manipulations of gene expression patterns lead to changes in projection patterns.

Subsequently, the activity of the resulting structural patterns could be tested in frameworks such as the Virtual Mouse Brain (Sanz-Leon et al. 2013; Ritter et al. 2013; Woodman et al. 2014) or it could be validated by electrophysiology-based experiments. These approaches could be useful for translational neuroscientists.

Taken together, we have built and validated a novel paradigm for linking gene expression and structural projection patterns in the mouse mesoconnectome, based on volumetric data from the Allen Institute and using a modified version of the Linked ICA method. A comparison with the DLSC technique and the preservation of the results under increasing data volume suggest robustness of the method in capturing independent components of shared variance across both modalities. Finally, our method presents a relevant framework through a number of use-cases, which could support assisting studies aiming to relate genes to brain function.

## Electronic supplementary material

Below is the link to the electronic supplementary material.

(ZIP 1.91 MB)

## References

[CR1] Amann R, Fuchs BM (2008). Single-cell identification in microbial communities by improved fluorescence in situ hybridization techniques. Nature Reviews Microbiology.

[CR2] Bakker R, Tiesinga P, Kötter R (2015). The scalable brain atlas: Instant web-based access to public brain atlases and related content. Neuroinformatics.

[CR3] Baruch L, Itzkovitz S, Golan Mashiach M, Shapiro E, Segal E (2008). Using expression profiles of caenorhabditis elegans neurons to identify genes that mediate synaptic connectivity. PLoS Computational Biology.

[CR4] Beckmann CF, Smith SM (2005). Tensorial extensions of independent component analysis for multisubject fmri analysis. NeuroImage.

[CR5] Bell AJ, Sejnowski TJ (1995). An information-maximization approach to blind separation and blind deconvolution. Neural Computation.

[CR6] Calhoun VD, Adali T, Giuliani NR, Pekar JJ, Pearlson GD (2006). Method for multimodal analysis of independent source differences in schizophrenia: combining gray matter structural and auditory oddball functional data. Human Brain Mapping.

[CR7] Castellano S, Balletto E (2002). Is the partial mantel test inadequate?. Evolution.

[CR8] Cepeda C, Wu N, Andr VM, Cummings DM, Levine MS (2007). The corticostriatal pathway in huntingtons disease. Progress in Neurobiology.

[CR9] Chamberlin NL, Du B, de Lacalle S, Saper CB (1998). Recombinant adeno-associated virus vector: use for transgene expression and anterograde tract tracing in the cns. Brain Research.

[CR10] Cheveé M, Robertson JDJ, Cannon GH, Brown SP, Goff LA (2018). Variation in activity state, axonal projection, and position define the transcriptional identity of individual neocortical projection neurons. Cell Reports.

[CR11] Cuberos H, Valle B, Vourch P, Tastet J, Andres CR, Bénédetti H (2015). Roles of lim kinases in central nervous system function and dysfunction. FEBS Letters.

[CR12] Daimon CM, Jasien JM, Wood WH, Zhang Y, Becker KG, Silverman JL, Crawley JN, Martin B, Maudsley S (2015). Hippocampal transcriptomic and proteomic alterations in the btbr mouse model of autism spectrum disorder. Frontiers in Physiology.

[CR13] De la Rossa A, Bellone C, Golding B, Vitali I, Moss J, Toni N, Lüscher C, Jabaudon D (2013). In vivo reprogramming of circuit connectivity in postmitotic neocortical neurons. Nature Neuroscience.

[CR14] Dodge Y (2008). The concise encyclopedia of statistics.

[CR15] Douaud G, Groves AR, Tamnes CK, Westlye LT, Duff EP, Engvig A, Walhovd KB, James A, Gass A, Monsch AU, Matthews PM, Fjell AM, Smith SM, Johansen-Berg H (2014). A common brain network links development, aging, and vulnerability to disease. Proceedings of the National Academy of Sciences.

[CR16] Economo MN, Viswanathan S, Tasic B, Bas E, Winnubst J, Menon V, Graybuck LT, Nguyen TN, Smith KA, Yao Z, Wang L, Gerfen CR, Chandrashekar J, Zeng H, Looger LL, Svoboda K (2018). Distinct descending motor cortex pathways and their roles in movement. Nature.

[CR17] Economo, M.N., Winnubst, J., Bas, E., Ferreira, T.A., & Chandrashekar, J. (2019). Single-neuron axonal reconstruction: The search for a wiring diagram of the brain. *Journal of Comparative Neurology*, 1–10. 10.1002/cne.24674 30859571

[CR18] Fakhry A, Zeng T, Peng H, Ji S (2015). Global analysis of gene expression and projection target correlations in the mouse brain. Brain Informatics.

[CR19] Fornito A, Arnatkeviit A, Fulcher BD (2019). Bridging the gap between connectome and transcriptome. Trends in Cognitive Sciences.

[CR20] Fornito A, Zalesky A, Bassett DS, Meunier D, Ellison-Wright I, Yücel M, Wood SJ, Shaw K, O’Connor J, Nertney D, Mowry BJ, Pantelis C, Bullmore ET (2011). Genetic influences on cost-efficient organization of human cortical functional networks. Journal of Neuroscience.

[CR21] French L, Pavlidis P (2011). Relationships between gene expression and brain wiring in the adult mouse brain. PLoS Computational Biology.

[CR22] French L, Tan PPC, Pavlidis P (2011). Large-scale analysis of gene expression and connectivity in the mouse brain: insights through data integration. Frontiers in Neuroinformatics.

[CR23] Fulcher BD, Fornito A (2016). A transcriptional signature of hub connectivity in the mouse connectome. PNAS.

[CR24] Gerfen CR, Economo MN, Chandrashekar J (2016). Long distance projections of cortical pyramidal neurons. Journal of Neuroscience Research.

[CR25] Glahn DC, Winkler AM, Kochunov P, Almasy L, Duggirala R, Carless MA, Curran JC, Olvera RL, Laird AR, Smith SM, Beckmann CF, Fox PT, Blangero J (2010). Genetic control over the resting brain. Proceedings of the National Academy of Sciences.

[CR26] Gǎmǎnuţ R, Kennedy H, Toroczkai Z, Ercsey-Ravasz M, Essen DCV, Knoblauch K, Burkhalter A (2018). The mouse cortical connectome, characterized by an ultra-dense cortical graph, maintains specificity by distinct connectivity profiles. Neuron.

[CR27] Goodman JV, Bonni A (2019). Regulation of neuronal connectivity in the mammalian brain by chromatin remodeling. Current Opinion in Neurobiology.

[CR28] Grange P, Bohland JW, Okaty BW, Sugino K, Bokil H, Nelson SB, Ng L, Hawrylycz M, Mitra MP (2014). Cell-type-based model explaining coexpression patterns of genes in the brain. PNAS.

[CR29] Grothe MJ, Sepulcre J, Gonzalez-Escamilla G, Jelistratova I, Schöll M, Hansson O, Teipel SJ, Initiative ADN (2018). Molecular properties underlying regional vulnerability to Alzheimers disease pathology. Brain: A Journal of Neurology.

[CR30] Groves AR, Beckmann CF, Smith SM, Woolrich MW (2011). Linked independent component analysis for multimodal data fusion. NeuroImage.

[CR31] Han Y, Kebschull J, Campbell R, Cowan D, Imhof F, Zador AM, Mrsic-Flogel TD (2018). The logic of single-cell projections from visual cortex. Nature.

[CR32] Harris JA, Oh SW, Zeng H (2012). Adeno-associated viral vectors for anterograde axonal tracing with fluorescent proteins in nontransgenic and cre driver mice. Current Protocols in Neuroscience.

[CR33] Hawrylycz M, Lein E, Guillozet-Bongaarts A (2009). An anatomically comprehensive atlas of the adult human brain transcriptome. Nature.

[CR34] Henry AM, Hohmann JG (2012). High-resolution gene expression atlases for adult and developing mouse brain and spinal cord. Mammalian Genome.

[CR35] Hooks BM, Papale AE, Paletzki RF, Feroze MW, Eastwood BS, Couey JJ, Winnubst J, Chandrashekar J, Gerfen CR (2018). Topographic precision in sensory and motor corticostriatal projections varies across cell type and cortical area. Nature Communications.

[CR36] Huang L, Kebschull JM, Fürth D, Musall S, Kaufman MT, Churchland AK, Zador AM (2020). Bricseq bridges brain-wide interregional connectivity to neural activity and gene expression in single animals. Cell.

[CR37] Hyvarinen A (1991). Fast and robust fixed-point algorithms for independent component analysis. IEEE Transactions on Neural Networks.

[CR38] Itahashi T, Yamada T, Nakamura M, Watanabe H, Yamagata B, Jimbo D, Shioda S, Kuroda M, Toriizuka K, Kato N, Hashimoto R (2015). Linked alterations in gray and white matter morphology in adults with high-functioning autism spectrum disorder: A multimodal brain imaging study. NeuroImage: Clinical.

[CR39] Ji S, Fakhry A, Deng H (2014). Integrative analysis of the connectivity and gene expression atlases in the mouse brain. NeuroImage.

[CR40] Kang HJ, Kawasawa YI, Cheng F, Zhu Y, Xu X, Li M, Sousa AMM, Pletikos M, Meyer KA, Sedmak G, Guennel T, Shin Y, Johnson MB, Krsnik Z, Mayer S, Fertuzinhos S, Umlauf S, Lisgo SN, Vortmeyer A, Weinberger DR, Mane S, Hyde TM, Huttner A, Reimers M, Kleinman JE, Sestan N (2011). Spatio-temporal transcriptome of the human brain. Nature.

[CR41] Kaufman A, Dror G, Meilijson I, Ruppin E (2006). Gene expression of caenorhabditis elegans neurons carries information on their synaptic connectivity. PLoS Computational Biology.

[CR42] Keil JM, Qalieh A, Kwan KY (2018). Brain transcriptome databases: A user’s guide. Journal of Neuroscience.

[CR43] Khibnik LA, Tritsch NX, Sabatini BL (2014). A direct projection from mouse primary visual cortex to dorsomedial striatum. PloS One.

[CR44] Kim EJ, Zhang Z, Huang L, Ito-Cole T, Jacobs MW, Juavinett AL, Senturk G, Hu M, Ku M, Ecker JR, Callaway EM (2020). Extraction of distinct neuronal cell types from within a genetically continuous population. Neuron.

[CR45] Kincses ZT, Horinek D, Szabo N, Toth E, Csete G, Stepan-Buksakowska I, Hort J, Vecsei L (2013). The pattern of diffusion parameter changes in alzheimers disease, identified by means of linked independent component analysis. Journal of Alzheimers Disease.

[CR46] Kobak, D., Bernaerts, Y., Weis, M.A., Scala, F., Tolias, A., & Berens, P. (2019). Sparse reduced-rank regression for exploratory visualization of multimodal data sets. *bioRxiv*.

[CR47] Kuo HY, Liu FC (2019). Synaptic wiring of corticostriatal circuits in basal ganglia: Insights into the pathogenesis of neuropsychiatric disorders. eNeuro.

[CR48] Langfelder P, Horvath S (2008). Wgcna: an r package for weighted correlation network analysis. BMC Bioinformatics.

[CR49] Le Bihan, D., & Breton, E. (1985). Imagerie de diffusion in vivo par résonance magnétique nucléaire. *Comptes rendus de l’Académie des sciences. Série 2, Mécanique, Physique, Chimie, Sciences de l’univers, Sciences de la Terre*.

[CR50] Lein ES (2007). Genome-wide atlas of gene expression in the adult mouse brain. Nature.

[CR51] Lein E, Borm LE, Linnarsson S (2017). The promise of spatial transcriptomics for neuroscience in the era of molecular cell typing. Science.

[CR52] Lein ES, Belgard TG, Hawrylycz M, Molnr Z (2017). Transcriptomic perspectives on neocortical structure, development, evolution, and disease. Annual Review of Neuroscience.

[CR53] Li Y, Chen H, Jiang X, Li X, Lv J, Peng H, Tsien JZ, Liu T (2017). Discover mouse gene coexpression landscapes using dictionary learning and sparse coding. Brain Structure and Function.

[CR54] Llera A, Wolfers T, Mulders P, Beckmann CF (2019). Inter-individual differences in human brain structure and morphology link to variation in demographics and behavior. eLife.

[CR55] Luo L, Callaway EM, Svoboda K (2018). Genetic dissection of neural circuits: A decade of progress. Neuron.

[CR56] Maglanoc LA, Kaufmann T, Jonassen R, Hilland E, Beck D, Landrø NI, Westlye LT (2020). Multimodal fusion of structural and functional brain imaging indepression using linked independent component analysis. Human Brain Mapping.

[CR57] Mairal J, Bach F, Ponce J, Sapiro G (2010). Online learning for matrix factorization and sparse coding. Journal of Machine Learning Research.

[CR58] Mancarci BO, Toker L, Tripathy S, Li B, Rocco B, Sibille E, Pavlidis P (2017). Cross-laboratory analysis of brain cell type transcriptomes with applications to interpretation of bulk tissue data. eNeuro.

[CR59] McColgan P, Gregory S, Seunarine KK, Razi A, Papoutsi M, Johnson E, Durr A, Roos RAC, Leavitt BR, Holmans P, Scahill RI, Clark CA, Rees G, Tabrizi SJ, Coleman A, Decolongon J, Fan M, Petkau T, Jauffret C, Justo D, Lehericy S, Nigaud K, Valabrégue R, Schoonderbeek A, t Hart EP, Moss DJH, Ghosh R, Crawford H, Papoutsi M, Berna C, Mahaleskshmi D, Reilmann R, Weber N, Labuschagne I, Stout J, Landwehrmeyer B, Orth M, Mayer I, Johnson H, Crawfurd D (2018). Brain regions showing white matter loss in huntingtons disease are enriched for synaptic and metabolic genes. Biological Psychiatry.

[CR60] Miller JA, Horvath S, Geschwind DH (2010). Divergence of human and mouse brain transcriptome highlights alzheimer disease pathways. Proceedings of the National Academy of Sciences.

[CR61] Ogata H, Goto S, Sato K, Fujibuchi W, Bono H, Kanehisa M (1999). Kegg: Kyoto encyclopedia of genes and genomes. Nucleic Acids Research.

[CR62] Oh SW (2014). A mesoscale connectome of the mouse brain. Nature.

[CR63] Pantazatos SP, Schmidt MF (2020). Toward establishing internal validity for correlated gene expression measures in imaging genomics of functional networks: Why distance corrections and external face validity alone fall short. reply to distance is not everything in imaging genomics of functional networks: Reply to a commentary on correlated gene expression supports synchronous activity in brain networks. Frontiers in Neuroscience.

[CR64] Polleux F (2005). Genetic mechanisms specifying cortical connectivity: Lets makesome projections together. Neuron.

[CR65] Razoux F, Russig H, Mueggler T, Baltes C, Dikaiou K, Rudin M, Mansuy IM (2017). Transgenerational disruption of functional 5-ht1ar-induced connectivity in the adult mouse brain by traumatic stress in early life. Molecular Psychiatry.

[CR66] Rice JA (2007). Mathematical statistics and data analysis.

[CR67] Richiardi J, Altmann A, Milazzo A-C, Chang C, Chakravarty MM, Banaschewski T, Barker GJ, Bokde ALW, Bromberg U, Büchel C, Conrod P, Fauth-Bühler M, Flor H, Frouin V, Gallinat J, Garavan H, Gowland P, Heinz A, Lemaître H, Mann KF, Martinot J-L, Nees F, Paus T, Pausova Z, Rietschel M, Robbins TW, Smolka MN, Spanagel R, Ströhle A, Schumann G, Hawrylycz M, Poline J-B, Greicius MD (2015). Correlated gene expression supports synchronous activity in brain networks. Science.

[CR68] Ritter P, Schirner M, McIntosh AR, Jirsa VK (2013). The virtual brain integrates computational modeling and multimodal neuroimaging. Brain Connectivity.

[CR69] Rittman T, Rubinov M, Vértes PE, Patel AX, Ginestet CE, Ghosh BCP, Barker RA, Spillantini MG, Bullmore ET, Rowe JB (2016). Regional expression of the mapt gene is associated with loss of hubs in brain networks and cognitive impairment in parkinson disease and progressive supranuclear palsy. Neurobiology of Aging.

[CR70] Rivals I, Personnaz L, Taing L, Potier MC (2007). Enrichment or depletion of a go category within a class of genes: which test?. Bioinformatics.

[CR71] Romero-Garcia R, Warrier V, Bullmore EEA (2019). Synaptic and transcriptionally downregulated genes are associated with cortical thickness differences in autism. Molecular Psychiatry.

[CR72] Romme IAC, de Reus MA, Ophoff RA, Kahn RS, van den Heuvel MP (2017). Connectome disconnectivity and cortical gene expression in patients with schizophrenia. Biological Psychiatry.

[CR73] Roy M, Sorokina O, McLean C, Tapia-Gonzlez S, DeFelipe J, Armstrong JD, Grant SGN (2018). Regional diversity in the postsynaptic proteome of the mouse brain. Proteomes.

[CR74] Rubinov M, Ypma RJF, Watson C, Bullmore ET (2015). Wiring cost and topological participation of the mouse brain connectome. PNAS.

[CR75] Sanz-Leon P, Knock SA, Woodman MM, Domide L, Mersmann J, McIntosh AR, Jirsa VK (2013). The virtual brain: a simulator of primate brain network dynamics.. Frontiers in Neuroinformatics.

[CR76] Satija R, Farrell J, Gennert D, Schier AF, Regev A (2015). Spatial reconstruction of single-cell gene expression. Nature Biotechnology.

[CR77] Savage MA, McQuade R, Thiele A (2017). Segregated fronto-cortical and midbrain connections in the mouse and their relation to approach and avoidance orienting behaviors. Journal of Comparative Neurology.

[CR78] Smit DJA, Stam CJ, Posthuma D, Boomsma DI, de Geus EJC (2008). Heritability of small-world networks in the brain: A graph theoretical analysis of resting-state EEG functional connectivity. Human Brain Mapping.

[CR79] Sperry RW (1963). Chemoaffinity in the orderly growth of nerve fiber patterns and connections. PNAS.

[CR80] Tasic B (2016). Adult mouse cortical cell taxonomy by single cell transcriptomics. Nature Neuroscience.

[CR81] Tasic B (2018). Single cell transcriptomics in neuroscience: cell classification and beyond. Current Opinion in Neurobiology.

[CR82] Tasic B (2018). Shared and distinct transcriptomic cell types across neocortical areas. Nature.

[CR83] Timonidis N, Bakker R, Tiesinga P (2020). Prediction of a cell-class-specific mouse mesoconnectome using gene expression data. Neuroinformatics.

[CR84] van den Heuvel MP, van Soelen ILC, Stam CJ, Kahn RS, Boomsma DI, Hulshoff Pol HE (2013). Genetic control of functional brain network efficiency in children. European Neuropsychopharmacology.

[CR85] Viereckel T, Dumas S, Smith-Anttila CJA, Vlcek B, Bimpisidis Z, Lagerström MC, Konradsson-Geuken, Wallén-Mackenzie (2016). Midbrain gene screening identifies a new mesoaccumbal glutamatergic pathway and a marker for dopamine cells neuroprotected in parkinsons disease. Scientific Reports.

[CR86] Wang Q, Ding S-L, Li Y, Royall J, Feng D, Lesnar P, Graddis N, Naeemi M, Facer B, Ho A, Dolbeare T, Blanchard B, Dee N, Wakeman W, Hirokawa KE, Szafer A, Sunkin SM, Oh SW, Bernard A, Phillips JW, Hawrylycz M, Koch C, Zeng H, Harris JA, Ng L (2020). The allen mouse brain common coordinate framework: A 3d reference atlas. Cell.

[CR87] Wang X-J (2020). Macroscopic gradients of synaptic excitation and inhibition in the neocortex. Nature Reviews Neuroscience.

[CR88] Winnubst J, Bas E, Ferreira TA, Wu Z, Economo MN, Edson P, Arthur BJ, Bruns C, Rokicki K, Schauder D, Olbris DJ, Murphy SD, Ackerman DG, Arshadi C, Baldwin P, Blake R, Elsayed A, Hasan M, Ramirez D, Santos] BD, Weldon M, Zafar A, Dudman JT, Gerfen CR, Hantman AW, Korff W, Sternson SM, Spruston N, Svoboda K, Chandrashekar J (2019). Reconstruction of 1,000 projection neurons reveals new cell types and organization of long-range connectivity in the mouse brain. Cell.

[CR89] Wolf L, Goldberg C, Manor N (2011). Gene expression in the mouse brain is associated with its regional connectivity. PLoS Computational Biology.

[CR90] Wolfers T, Llera Arenas A, Onnink AMH, Dammers J, Hoogman M, Zwiers MP, Buitelaar JK, Franke B, Marquand AF, Beckmann CF (2017). Refinement by integration: aggregated effects of multimodal imaging markers on adult adhd. Journal of Psychiatry and Neuroscience.

[CR91] Woodman MM, Pezard L, Domide L, Knock S, Sanz Leon P, Mersmann J, McIntosh AR, Jirsa VK (2014). Integrating neuroinformatics tools in the virtual brain. Frontiers in Neuroinformatics.

[CR92] Wu Z-M, Llera A, Hoogman M, Cao Q-J, Zwiers MP, Bralten J, An L, Sun L, Yang L, Yang B-R, Zang Y-F, Franke B, Beckmann CF, Mennes M, Wang Y-F (2019). Linked anatomical and functional brain alterations in children with attention-deficit/hyperactivity disorder. NeuroImage: Clinical.

[CR93] Zhu F, Cizeron M, Qiu Z, Benavides-Piccione R, Kopanitsa MV, Skene NG, Koniaris B, DeFelipe J, Fransén E, Komiyama NH, Grant SGN (2018). Architecture of the mouse brain synaptome. Neuron.

