# Supplementary material for: Uncovering Statistical Links Between Gene Expression and Structural Connectivity Patterns in the Mouse Brain
Source: Neuroinformatics. 2021 Mar 11;19(4):649–67. doi: 10.1007/s12021-021-09511-0 (PMC8566442; doi:10.1007/s12021-021-09511-0)
Supplement: Supplementary file 1 — (ZIP 1.91 MB) [file 12021_2021_9511_MOESM1_ESM.zip › Supplementary_Manuscript_2.pdf]

Neuroinformatics manuscript No.
(will be inserted by the editor)

Uncovering statistical links between gene expression and structural connectivity patterns in the mouse brain

Nestor Timonidis · Alberto Llera · Paul H.E. Tiesinga

Received: date / Accepted: date

1 Supplemental Methods

1.1 Linked ICA

The original Linked ICA equation for modelling the input data as a sum of independent components is:

$$Y^k_{n,t,r} = \sum_{i=1}^{L} X^k_{n,i} W^k_{t,i} H^k_{i,r} + E^k_{n,t,r} \tag{1}$$

where k represents a modality group from K groups, L represents the number of independent components, n represents a voxel from N_k voxels, t represents a modality from T_k data modalities within group k and r represents a subject from R subjects, for which all modality groups are available. X is a stack of spatial maps across components for each modality group, W represents the modality weightings of the components in modalities of different groups and H represents the subject weights, which are shared across all groups.

The first major change was to use the voxels as the shared dimension rather than the subjects, since both datasets have been registered, aligned and scaled to the same reference space. Therefore, the spatial map size $N_k = N$ was constant across all modality groups and replaced R in the original formula (eq. S 1). Subsequently, the H representation changed from subject weights to spatial maps, since it now represented the variance of components over space.

The second change was to redefine X, given that it was no longer defined by voxels and components. The two data

Nestor Timonidis
[1]Neuroinformatics department, Donders Centre for Neuroscience, Radboud University Nijmegen, Heyendaalseweg 135, 6525 AJ Nijmegen, the Netherlands
Tel.: +31-649552777
E-mail: n.timonidis@donders.ru.nl

Alberto Llera Arenas
[1] Department of Cognitive Neuroscience, Radboud University Medical Centre, Kapittelweg 29, 6525 EN Nijmegen, the Netherlands
[2] Centre for Cognitive Neuroimaging, Donders Institute for Brain, Cognition and Behavior, Kapittelweg 29, 6525 EN Nijmegen, the Netherlands

Paul Tiesinga
[1]Neuroinformatics department, Donders Centre for Neuroscience, Radboud University Nijmegen, Heyendaalseweg 135, 6525 AJ Nijmegen, the Netherlands

modalities could not form a common modality group since they did not share any other common dimension besides the voxels. For that reason, we defined both data modalities as two distinct modality groups and that resulted in the symbols t and T_k being removed from the new formula as redundant. Modality group 1 corresponded to the gene expression modality and modality group 2 corresponded to the projection density modality. Since each modality had a different set of features, we redefined R as the feature dimension. with R_1 representing genes and R_2 representing injection sites/source areas. The resulting changes can be seen in Main eq. 1.

1.2 Dictionary Learning and Sparse Coding (DLSC)

In the DLSC method, the input data are being represented as a linear combination of positive and non-orthogonal basis vectors, defined as dictionaries, and their sparse coefficients, defined as atoms. In contrast to the classical PCA factorization method (Pearson, 1901), DLSC uses the l_1 constraint to create sparse non-orthogonal coefficients.(Mairal et al., 2010). The optimization problem corresponding to the Dictionary Learning and Sparse Coding method is:

$$\begin{aligned} (D,a) &= \operatorname{argmin} \frac{1}{2}||X - Da||_2^2, \\ &||a||_1 \leq \lambda, \quad ||a|| > 0, \quad D_{ij} > 0, \quad \forall i,j \in \mathbb{N} \end{aligned} \tag{2}$$

With respect to our study, X represents the gene expression matrix (voxels $\times$ genes), a represents the atoms or coefficients of individual genes to each dictionary (dictionaries $\times$ genes) and D represents the dictionary-based spatial maps in brain space (voxels $\times$ dictionaries).

This formula represents the DLSC problem as a regularized linear least-squares problem, which can be seen as a l_1 counterpart to eq. S 3, and the block-coordinate descent approach is being used to update the atoms and dictionaries in a sequential fashion, for which more information can be found in (Mairal et al., 2010). Therefore, the number of dictionaries is not bound by the number of nonzero singular values in the array, hence allowing for representations of the input data by any finite number of dictionaries.

Regarding hyperparameter selection, we chose the number of dictionaries to be 200 for both datasets and their concatenation, hence keeping the same reduced dimensional space for both approaches in order to ensure spatial homogeneity. In addition, we set the sparsity constraint to 1, which was the default value of the library (see Main section 1.13). For the *exclusive* and the *concat* dictionaries, the chosen number of gene atoms accounted in both cases for 70% of the variance of all genes and the projection atoms accounted for 60% and 71% of the variance of all projection patterns, respectively.

1.3 Ridge Regression

Ridge regression or Tikhonov regularization is a penalized version of the classical linear regression analysis (Tikhonov and Arsenin, 1977; Friedman et al., 2009). Specifically, it adds the l_2 norm of the coefficients, which are estimated from the ordinary least-squares formula, multiplied by a shrinkage hyperparameter λ, to the objective function:

$$\hat{w} = \underset{w \in R}{\operatorname{argmin}}(||y - Xw - b||_2^2 + \lambda||w||_2^2) \tag{3}$$

where X represents the input array comprised of the independent variables, y represents the vector/array comprised of the dependent variable or variables that are to be fitted, b is an intercept term and $\hat{w}$ represents the coefficient vector w that minimizes the residuals between the actual y and the fitted $\hat{y} = Xw + b$.

The inclusion of the l_2 norm constraint is very effective in datasets where the number of variables or columns is greater than the number of samples or rows.

1.4 Nested k-fold cross-validation

K-fold cross-validation is a technique for evaluating the generalization performance of a model, meaning its performance on previously unseen data (Bishop, 2006). It is based on splitting the data by k disjoint folds and training k different models wherein each model gets trained by k-1 folds and the remaining fold tests the model, while the overall generalization performance is quantified by the average performance across folds (Kohavi, 1995).
This technique can be biased in the cases where it is used for optimizing the hyperparameters of the model besides testing the generalization performance, since the same folds will be used for both tasks. In nested k-fold cross validation the training folds are nested by k-inner folds, labeled as internal training and validation folds, that assess the most optimal hyperparameter for a given fold (see figure S2). Therefore, the method ensures that the data partitioning will separate folds which validate hyperparameters, fit the data with the selected hyperparameters and test the fitted data (Varma and Simon, 2006).
For nested k-fold cross-validation, we chose 6 external folds for evaluating the generalization performance for the ridge-regression-based model and 6 internal folds for selecting the most optimal l_2 value among multiple values in the range [10^{-3} - 10^{5}] (for the model description see section S 1.3 and for the model usage see section S 1.5).

1.5 DLSC-based Predictions and comparison with Linked ICA

We evaluated the capability of DLSC to predict projection density patterns and compared it to the reconstruction accuracy of Linked ICA. Such findings would provide additional evidence that these methods can find links between genes and structural connections in the mouse brain. Specifically, we utilized the ridge regression method (Tikhonov and Arsenin, 1977; Friedman et al., 2009) to build models that predicted projection patterns from each source area independently using the *concat* dictionaries. We then compared the performance of the DLSC-based predictions with the Linked ICA-based reconstructions, per source area individually. Finally, the generalization performance of the predictive models was judged using the nested k-fold cross-validation (Varma and Simon, 2006) technique. For more details regarding the formulas of the ridge regression model and the nested k-fold cross-validation technique, see sections S 1.3 and S 1.4.
The projection density predictions using the spatial maps from the *concat*-dictionaries resulted in a total r^2 of 0.46. We compared the predictive accuracy of DLSC with the reconstruction accuracy of Linked ICA per projection pattern of an individual source/injection, resulting in two equally-sized distributions of r^2 values across all injections. When comparing these distributions, our paired t-test analysis revealed a t-statistic of -30.6 and a p-value of 10^{-117}, hence showing that Linked ICA has a higher accuracy mean compared to DLSC (see figure S1).

1.6 Modality-specific spatial contribution

A reasonable question arising was about the amount of variance that each modality contributed to the spatial map of a given component. To answer that question, we built modality-specific spatial maps for all local and global components of interest. First, tract-tracing maps were formed for a particular injection group by estimating the linear combination of projection patterns from that group multiplied with their injection coefficients. Second, gene

expression maps were formed by the inner product of all genes with their coefficients of each injection group. Both products were scaled by their modality weighting (W). The formula for the linear combination is:

$$\hat{H}_i^k = (X_i^k W_i^k)^T Y^k \tag{4}$$

where $\hat{H}_i^k$ represents the spatial map of modality k for the amount of variance that is contributed by the i-th independent component. See eq.1 of the main text for the definition of the symbols. For disambiguation purposes, we shall refer to the original Linked ICA spatial maps as shared spatial maps.

The following step was to estimate the variance of the modality-specific maps in the areas of interest. First, we built voxel masks of high variance by thresholding and binarizing all maps using the 1st and 99th percentile of their variance. This enabled us to determine the influence of projection patterns on the shared spatial maps by checking the overlap of high-variance areas in both the shared maps and the tract-tracing maps. For the respective gene maps, we adopted a color-coding strategy for determining the influence of both modalities in the spatial maps, since we are interested in the influence of genes in relation to the projection patterns.

Consequently, we used the thresholded masks of gene expression, projection density and the shared ones in order to find three different groups of voxels, for each component independently. The first group of voxels represented areas where all three masks had a non-zero value, meaning that a given component exhibited high variance in a brain area that was driven by both modalities, and we color-coded this bimodal group as green. The second group of voxels represented areas where the gene expression and shared masks had a non-zero value, meaning that a given component exhibited high variance in a brain areas that was driven by the gene expression data, and we color-coded this gene expression driven group as blue. The third group of voxels was a projection density driven group, similarly to the previous one, and was color-coded as red. A final group of voxels with high variance in the shared masks but not in either of the modality-exclusive masks was color-coded as yellow. Main figure 6 and figs. S3 - S5 contain representative examples of color-coded maps.

For each component of interest, we counted the number of voxels in each brain region belonging to the gene expression exclusive mask, to the projection density exclusive mask and to the bimodal mask, hence creating counts for each category. Finally, we reported the brain areas most strongly represented by each category across components (see tables S5a - S7c).

1.7 Gene Enrichment Analysis

Gene ontology (GO) enrichment analysis is a popular bioinformatics-based analysis (Rivals et al., 2007) that is useful for validating the biological context of a group of genes. GO enrichment analysis aims at finding functional annotations for which a gene group of interest is significantly enriched, through the means of a statistical test such as the hypergeometric test (Rice, 2007).
Given a gene set that emerges from an experiment, either associated with a disease in GWAS or up-regulated for an experimental condition, and a tested annotation that is enriched among a number of genes, the hypergeometric test estimates the statistical significance of enrichment of these genes in relation to randomly drawn and equally sized gene subsets from a gene set defined as global (Rivals et al., 2007).

For our specific analysis, we utilized annotations and gene sets from the Kyoto Encyclopedia of Genes and Genomes (*KEGG*) pathway database and from the *Org.Mm.eg.db* database. The *Org.Mm.eg.db* database provides annotations for the mouse genome based on the Entrez gene identifiers from the NCBI database (see main table 2). The *KEGG pathway* database contains information about biochemical pathways related to gene and molecule interactions (Ogata et al., 1999). The reason for using both databases was because they provide annotations that could serve as useful validation tools for assessing the biological relevance of the selected components. *Org.Mm.eg.db* contains annotations related to cellular components, such as synaptic functions, and *KEGG* contains annotations about neurotransmitter-related properties of potentially enriched cells, such as gabaergic or glutamatergic.

1.8 Global Independent Components

In order to evaluate the reproducibility of the independent components found across the different injection groups (vis, mrn, cp), we compared their spatial maps with the respective global ones. The comparison was made by estimating the Pearson's correlation coefficient (rho) between local and global components. We then assembled local-global component pairs by assigning for each local component of interest the global component with the highest rho. For each pair, we examined the p-value for the null hypothesis of uncorrelated spatial maps, and we compared the rho and p-values between the gene and injection coefficients to test whether a similar correlation could be found for their modality coefficients. Since the components of interest were 11 in total, a bonferroni correction was used for assessing the correlation significances by dividing the classical p-value cutoff of 0.05 by 11, thus resulting in a threshold of 0.004.

As shown by the local-global pair comparison (see main table 3), significant correlations can be found in both the spatial maps and the gene coefficients for all local-global pairs ($p < 0.004$). At the injection coefficient domain, only 5 pairs were shown to exhibit significant correlation: vis components 0, 4 and 7 with global components 0, 5 and 23, respectively, cp component 0 with global component 0 and mrn component 0 with global component 0). The respective components of the global analysis that were paired with the local components were 0, 1, 2, 4, 5, 10 and 23.

The spatial correlations can be validated when examining the spatial maps of the selected global components (see main figure S5 and table S4c). A number of areas were highlighted by the color-coded maps as having high variance in both the modality-specific and the shared spatial maps, areas with exclusively high variance in the gene expression and shared spatial maps and areas with exclusively high variance in the projection density and shared spatial maps (see main figure S5 and table S7c).

1.9 Linked ICA and anatomical parcellation

We assessed the capability of our local and global analyses to provide spatial patterns with anatomically cohesive segments. In particular, we applied the spectral clustering algorithm (von Luxburg, 2007) to the global components, in order to find groups of voxels that were clustered by each analysis. As a ground truth, we used the anatomical parcellation provided by ARA CCF v3.0 of the Allen Institute (Wang et al., 2020). We intended to calculate a moderate number of clusters for visualization purposes. Hence, we set the number of clusters equal to 170, which corresponded to a medium scale parcellation scheme that defines brain subregions but does not include distinction by layers (see supplementary file 2 for the aforementioned areas).

We selected the radial basis function, to build a kernel matrix in voxel space as input to the spectral clustering algorithm:

$$K(v_1, v_2) = \exp -\gamma ||v_1 - v_2||^2 \quad (5)$$

where v_1,v_2 are two voxels from the brain space and γ is a coefficient that was set to 1.0. The clustering quality was assessed using the normalized mutual information (NMI) measure (Strehl and Ghosh, 2002). NMI is a variation of the classical mutual information (MI) measure, which estimates the shared entropy between two variables (Cover and Thomas, 1991). In NMI, classical MI is normalized by the marginal entropies of the variables under

comparison:

$$
\begin{aligned}
U(X,Y) &= \frac{I(X,Y)}{\sqrt{(H(X)+H(Y))}} \\
I(X,Y) &= \sum_{x\in X}\sum_{y\in Y} p(x,y)\log\frac{p(x,y)}{p(x)p(y)} \\
H(X) &= \sum_{x\in X} p(x)\log p(x)
\end{aligned}
\tag{6}
$$

where X and Y correspond to the two variables, I and H are the MI and marginal entropy measures, while p(x), p(y) and p(x,y) correspond to the marginal and joint probability distribution of X and Y, respectively.

The global components yielded an NMI score of 0.48 (fig. S6). A p-value of 0 was obtained by randomly permutating the clustering labels 1000 times, estimating the NMI score between the permuted labels and the ARA parcelation and then calculating the percentage of comparisons with an NMI greater than the original one. A lack of higher NMI can partly be attributed to the assymmetry across hemispheres observed in the spatial maps with a relative contribution from both the gene expression and projection density datasets.

The sagittal sections shown at the bottom row of fig. S6 exhibit stripe-like patterns across the superior-inferior axis. These patterns have also been reported in (Li et al., 2017), where the DLSC method was applied at the gene expression dataset. The conclusion drawn in this work was that the stripes were due to the sampling inconsistencies of AMBA caused by the 200 μm interspacing along the posterior-anterior axis (Lein et al., 2007). We agree with this assessment and hypothesize that the relative contribution of the gene expression modality to the global components transfers this effect to the spatial maps of Linked ICA.

1.10 Integrating Neuronal Morphologies

We obtained axonal morphologies of $\sim$ 1100 neurons from the MouseLight database (Economo et al., 2016), whose cell bodies were primarily located in the thalamus, motor cortex, subiculum and hypothalamus. As described in (Winnubst et al., 2019), the morphologies were reconstructed through a serial process of viral projection labeling, high resolution imaging and anatomical segmentation. First, labeling was achieved with injecting P56 wild-type male mice with a combination of two AAV viruses. The combined viruses were high-titer AAV expressing cre-recombinase (AAV Syn-iCre) and a low-titer virus coding for a fluorescent reporter (AAV CAG-Flex eGFP/tdTomato). Second, they used serial two-photon tomography with an integrated vibratome for image production. Finally, they applied a semi-automatic approach that was supplemented by manual labeling for axonal branch segmentation and reconstruction. This dataset is currently the most extensive collection of single-neuron long-range projection data, alongside the one presented in (Han et al., 2018), and bridges the gap between local microcircuits and brain-wide axonal projections. A prime example was the capability to identify cell-types with hitherto unknown projection patterns, such as in the case of zona incerta and subiculum neurons (Winnubst et al., 2019).

The single-neuron projection data needed more preprocessing than the other two modalities, given that they were not represented as volumetric data. First, we downloaded the *.swc* files from the Mouselight database (http://ml-neuronbrowser.janelia.org/). Second, we mapped the coordinates into 100 μm^3 voxels by multiplying each coordinate by 10^{-3}, given that their original resolution was 1 μm^3. This initial mapping allowed the data to be plotted overlaid with the 100 μm^3 projection density volumes. Subsequently, we converted the coordinates from their LIP orientation (left-right, inferior-superior, posterior-anterior) to the PIR orientation of CCFv3. This was done by switching elements between the first and the third dimension, followed by mirroring the elements of the third dimension. By repeating the downsampling and pre-processing steps, as described in section 2.1.3, the resulting 2D single-neuron array consisted of 63113 voxels $\times$ 1103 neurons.

The single-neurons projections span most of the brain but each neuron has highly sparse projections. For that reason, we selected projection subsets from the motor cortex (referred to as *MO* group), which was the most densely sampled brain area of the Mouselight database. In particular, it comprised 300 out of 1103 neurons and 17 injections out of 498.

For the *MO* local analysis, we initially performed a decomposition by selecting 17 components as the minimum rank across the three modalities. However, the high sparsity of the single-neuron data resulted in most components being dominated by the single-neuron data. Thus, we repeated the decomposition with a lower model order of 10 components, for forcing a more balanced contribution of the three modalities across the components. From these results, we selected as components of interest the ones having a non-zero contribution from the three data modalities. This meant that the component variance was partly explained by all of the modalities (see figure S8 for the relative modality contributions across components). We also selected ICAs 1, 2, 3, 8 and 9 because they had strong contributions from either the gene expression or the single-neuron modality. This would enable us to analyse spatial patterns with respect to particular gene groups or cell-types.

Table S8 highlights brain areas with high shared variance from the three modalities across components 1, 2, 3, 8 and 9. A number of these brain areas were also highlighted in (Winnubst et al., 2019), including the motor areas, somatosensory areas, ectorhinal areas, medulla, midbrain, pons, striatum, olfactory areas and corpus callosum, as well as the ventral, lateral and geniculate groups of the dorsal thalamic nuclei. Linked ICA can therefore uncover the motor cortex-related pathways mentioned in the aforementioned study.

Given that projections from the motor cortex were weakly sampled in the projection density data, we wanted to assess the additive effects of single-neuron data as a third modality in the fusion. Hence, we repeated the MO group analysis by removing the single-neuron data from the integrated modalities, while keeping the same number of 10 components. Subsequently, we removed the projection density data and repeated the factorisation with the gene expression and single-neuron data using 80 components given the higher sampling size of $\sim$1100 neurons. This lead to one trimodal and two bimodal MO group decomposition. We labeled the two bimodal factorisations as gene-injection and gene-neuron MO, while the original one was labeled as trimodal MO.

Subsequently, we correlated the spatial maps from the two decompositions, similarly to the global to local correlations shown in Main table 3. As shown in table S9, for each trimodal MO component there existed a bimodal MO component with a high and significant correlation. Exceptions existed for two trimodal-to-gene-injection pairs 1-0 and 8-9 (table S9 a), which had significant correlations at the level of spatial maps and gene coefficients but not at the level of injection coefficients ($p \geq 0.05$). This could be attributed to the low sampling size of the motor cortex injection (17 out of 498). Besides these two cases, all other correlations were significantly correlated at the level of spatial maps, genes and injections. Thus, results were quite preserved when including the single-neuron data. This suggests that neuronal reconstructions could be used as a complement to tract-tracing data when identifying components from sparsely sampled brain regions.

1.11 Predictive Model

As shown in the modified version of Linked ICA (Main eq. 1), the spatial map H is shared by two linear equations that explain the reconstruction of two data modalities with common independent components in the spatial dimension. Therefore, by estimating the pseudoinverse of the X^1W^1 array, which represents the feature coefficients multiplied by the modality weightings for modality 1, it is possible to formulate H as a function of modality 1 and subsequently formulate modality 2 a function of modality 1. The reformulation of modality 2 can be done either at the level of feature-coefficients (X matrix) or at the level of the original dataset (Y matrix), with H defined as based on modality k

$$H = f(Y^k, X^k, W^k) = [(X^kW^k)^T X^kW^k]^{-1}(X^kW^k)^T(Y^k - E^k) \tag{7}$$

yielding prediction for modality *k'* in terms of *Y*

$$Y^{k'} = X^{k'}W^{k'}H = X^{k'}W^{k'}f(Y^k, X^k, W^k) + E^{k'} \tag{8}$$

and *X*

$$X^{k'} = [(Y^{k'} - E^{k'})H^T](HH^T)^{-1}(W^{k'})^T[W^{k'}(W^{k'})^T]^{-1} \tag{9}$$

where $k \neq k'$ and E^k contains the modality-dependent additive noise, as shown in Main eq. 1. In the case where k represents the gene expression modality and k' represents the projection density modality, then we have a predictive model where changes in the gene expression of brain areas can lead to changes in the incoming projection density to these areas. This would allow us to test a number of hypotheses related to how pertubations in gene expression can lead to changes in connectivity density across multiple brain areas.

For this demonstration to succeed, causal links need to exist between the two modalities. Either both datasets should be sampled during closely related time-points in development or the gene expression data should contain traces of the development processes that have generated the projections (French and Pavlidis, 2011). Therefore, we would still need experimental manipulations to validate the tests (Polleux, 2005; Miller et al., 2010; De la Rossa et al., 2013; Daimon et al., 2015; Razoux et al., 2017; Goodman and Bonni, 2019). As a surrogate for experiments, we used the coefficient matrices (X) to test the fidelity of the predictions. The rationale is that controlled manipulations would create a specific and testable effect on the coefficient level. This means that changes in the feature coefficients of the projection density modality (X^2) can be expressed as a function of the changes in the feature coefficients of the gene expression modality (X^1), which is shown at eq. S 9.

We tested the predictive model under two manipulations. The first manipulation consisted of copying the expression-coefficients of all genes from component 44 to 45. The second manipulation consisted of swapping the expression-coefficients of all genes between components 44 and 45. We selected the *vis* injection group for the test given its densest set of 49 components compared to the other injection groups. We also selected higher indexed components because they had a low correlation with other components at the level of injection coefficients (see fig. S9). Based on the formula provided by eq. S 9, changes in X^1 would result in changes in *H*, labeled as $\tilde{H}$, and followed by changes in X^2, labeled as $\tilde{X^2}$. Therefore, we used Pearson's rho to measure the correlation between *H* and $\tilde{H}$ and between X^2 and $\tilde{X^2}$.

As shown in figure S9, both manipulations resulted in the same outcome for both $\tilde{H}$ and $\tilde{X^2}$. We observed a high correlation at the level of the injection coefficients between components 0 and 1 and between components 2-4, which have not been manipulated. This effect was not found in the spatial map level, which can be attributed to the diagonal covariance prior that has been imposed on the Linked ICA algorithm (Groves et al., 2011). The correlations could be explained by similar projection patterns across injections that reside in the same region.

We conducted this preliminary test as a proof of concept that Linked ICA can be used for predictive purposes. By linking two data modalities and modifying the objective function, it can allow for expressing one modality as a function of another. In future works we intend to integrate prior information and apply a full bayesian inference to the predictive model, in order to fully take advantage of the original Linked ICA algorithm.

1.12 Testing unseen data using Linked ICA

As a sanity check, we wanted to test the capability of Linked ICA to successfully reconstruct data that were not used in the shared factorisations performed in this analysis. The rationale is that the reconstruction performance will depend on the latent space proximity between the tested variable and the ones used in the analysis.

To perform this test, we repeated the vis group analysis by removing a number of variables or features from the datasets. Specifically, we removed 3% (99 out of 3318) of genes of the gene expression dataset, but we did not remove injections from the projection density dataset, given the limited amount of samples in the latter. This resulted in a subset analysis labeled vis sub.

The gene subset $\hat{g}$ that was left out of the vis sub, was encoded in the latent space using a variation of eq. S 9:

$$X^1_{\hat{g}} = [Y^1_{\hat{g}} H^T](HH^T)^{-1}(W^1)^T[W^1(W^1)^T]^{-1} \quad (10)$$

where value 1 represents the gene expression modality, $Y^1_{\hat{g}}$ contains the original expression values of $\hat{g}$ in voxel space and $X^1_{\hat{g}}$ contains the coefficients of the $\hat{g}$ across all vis sub components. Afterwards we decoded or reconstructed $X^1_{\hat{g}}$ in the voxel space using a variation of Main eq. 1:

$$\tilde{Y}^1_{\hat{g}} = X^1_{\hat{g}} W^1 H \quad (11)$$

where $\tilde{Y}^1_{\hat{g}}$ contains the reconstructed values. The total reconstruction accuracy was measured by calculating the mean squared error (MSE) between the actual and reconstructed spatial gene patterns, as shown in figure S10. The calculated MSE had a median of 0.28 and an iqr of 0.99. 75 % of tested genes had an MSE lower than 1.0.

Since the aim of this method is to find links between multiple modalities, reconstruction of each independent modality is not expected to be optimal. This is evident when examining the reconstruction accuracy of the gene expression modality that yielded an r^2 of 0.68 (see Main section 3.3, table 5a). As a consequence, any set of genes not used in the analysis is expected to be suboptimally reconstructed. This test was a simple sanity check, however, since to update the method to account for batches of unseen data will require additional modifications in its implementation.

1.13 Code

The visualizations of the spatial maps were created with the fsleyes tool, version 0.32.0, obtained from the FMRIB Software Library (fsl) version 6.0.3. Main table 2 contains references for all mentioned software packages, libaries, websites and databases. The gene Entrez ids have been acquired as metadata from Allen Mouse Brain Atlas. The *org.Mm.eg.db* database has been downloaded from the bioconductor website, and the ontology analysis related to it has been performed with the hyperGTest function from the GOHyperGParams-class package of R. The online access of the *KEGG* pathway database and the related ontology analysis have been done using the enrichKEGG function from the clusterProfiler package of R. Both *Org.Mm.eg.db* and *KEGG*-based functions utilized the hypergeometric test followed by a false discovery rate (FDR) test correction (Gold et al., 2009).

The downsampling of the projection density data was achieved using the *Nilearn* library of the *Python* programming language. Additionally, we used implementations of the DLSC, ridge regression and nested k-fold cross validation methods from the Python-based *scikit-learn* library. Last but not least, the Python-based *NumPy* and *SciPy* libraries were utilized for array-related numerical operations and for univariate statistical analyses, respectively.

2 Supplemental Figures

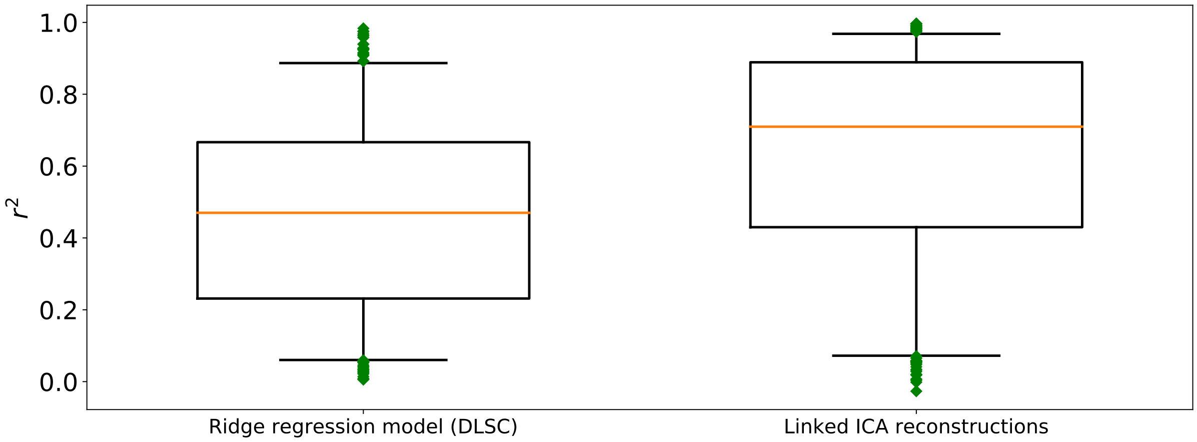

Fig. S1: Comparison of predictive accuracy between models trained using the DSLC dictionaries and the reconstruction accuracy of the Linked ICA model. y-axis: r^2 scores. x-axis: prediction performances from the ridge-regression-based models (left) and reconstruction performances from the Linked ICA (right). orange line: mean. box: 25-75 percentiles. bottom/top lines: 5-95 percentiles. green points: outliers exceeding the 5-95 percentiles. Each r^2 point represents the performance of a model in predicting or reconstructing the projection pattern of a particular injection. The statistic for the paired t-test between both distributions is -30.6 with a p-value of 10^{-117}.

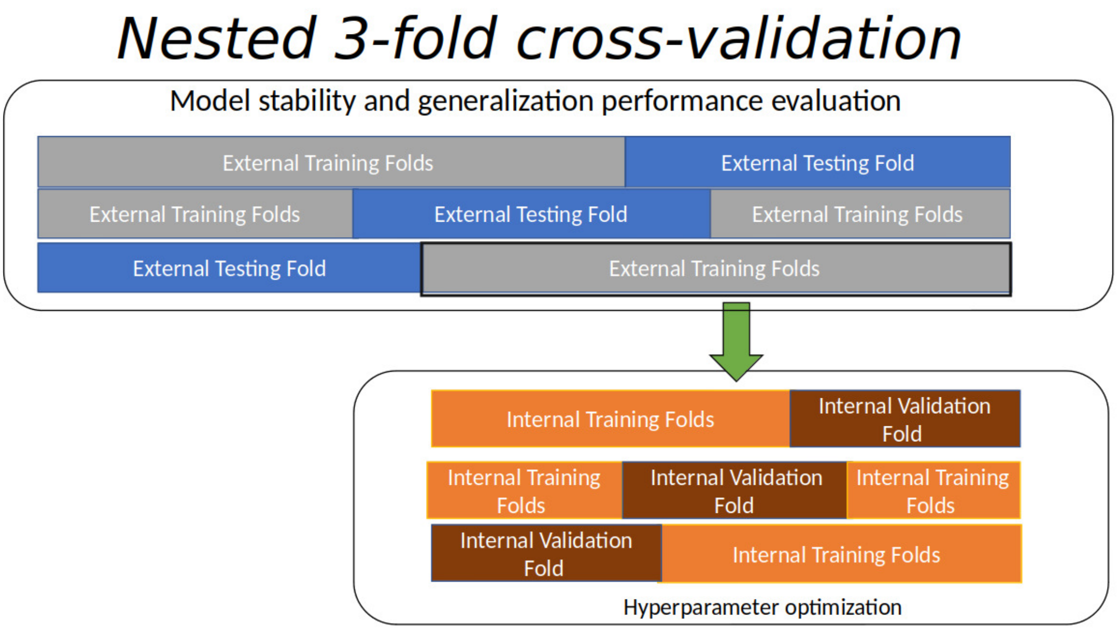

Fig. S2: Schematic describing the structure of the nested cross-validation method.

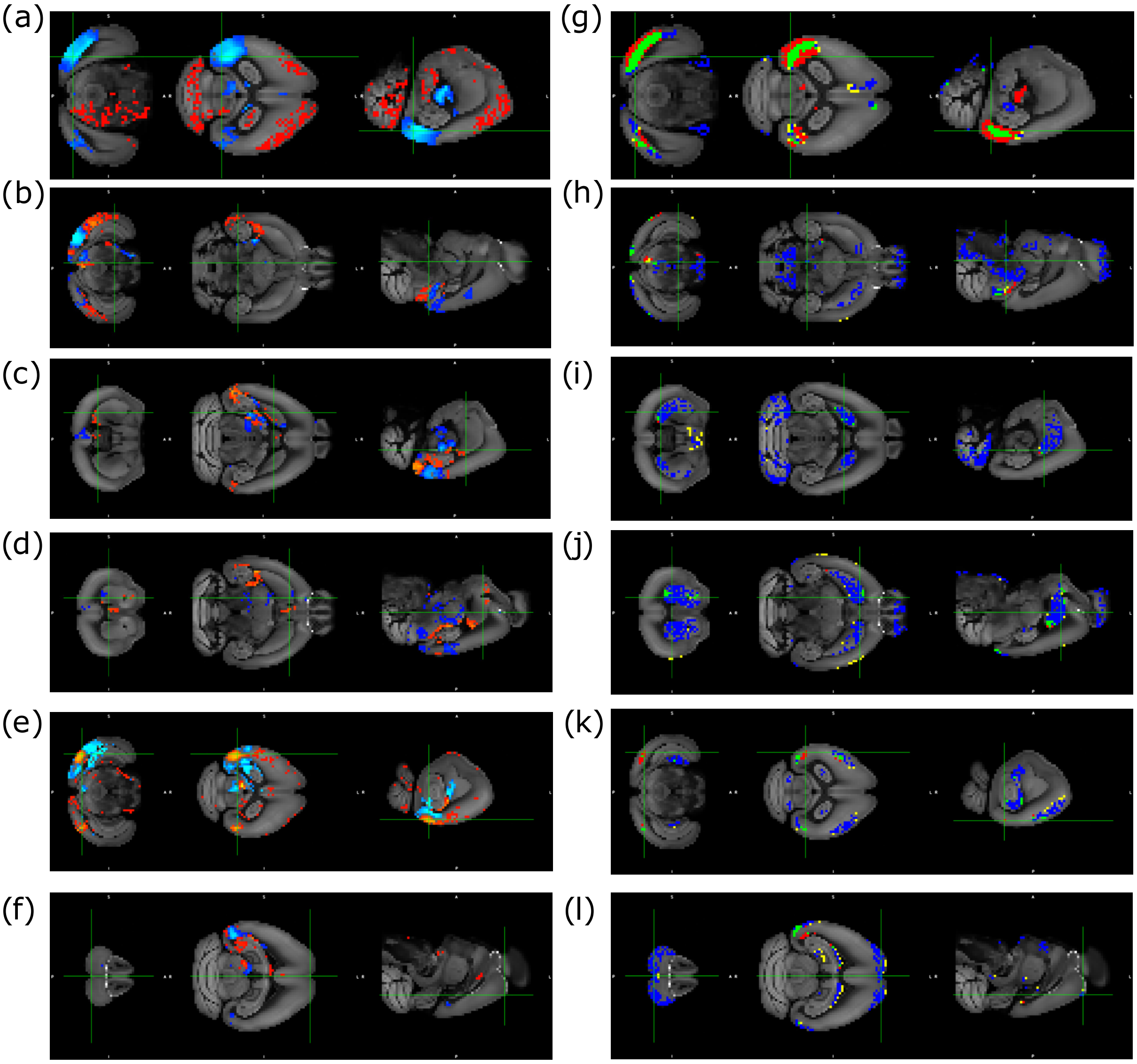

Fig. S3: Spatial maps for components of interest from the vis injection group. (a-f) tract-tracing maps (see section S 1.6 for more details). (g-l) color-coded spatial maps used to identify which modality drives the component's variation in the regions of interest. The thresholding and color convention for the spatial map visualizations is similar to the one used in Main figure 2 and a detailed description of the color-coding convention can be found in section S 1.6. In each column we show components 0, 2, 4, 5, 7 and 8, in the order of reference.

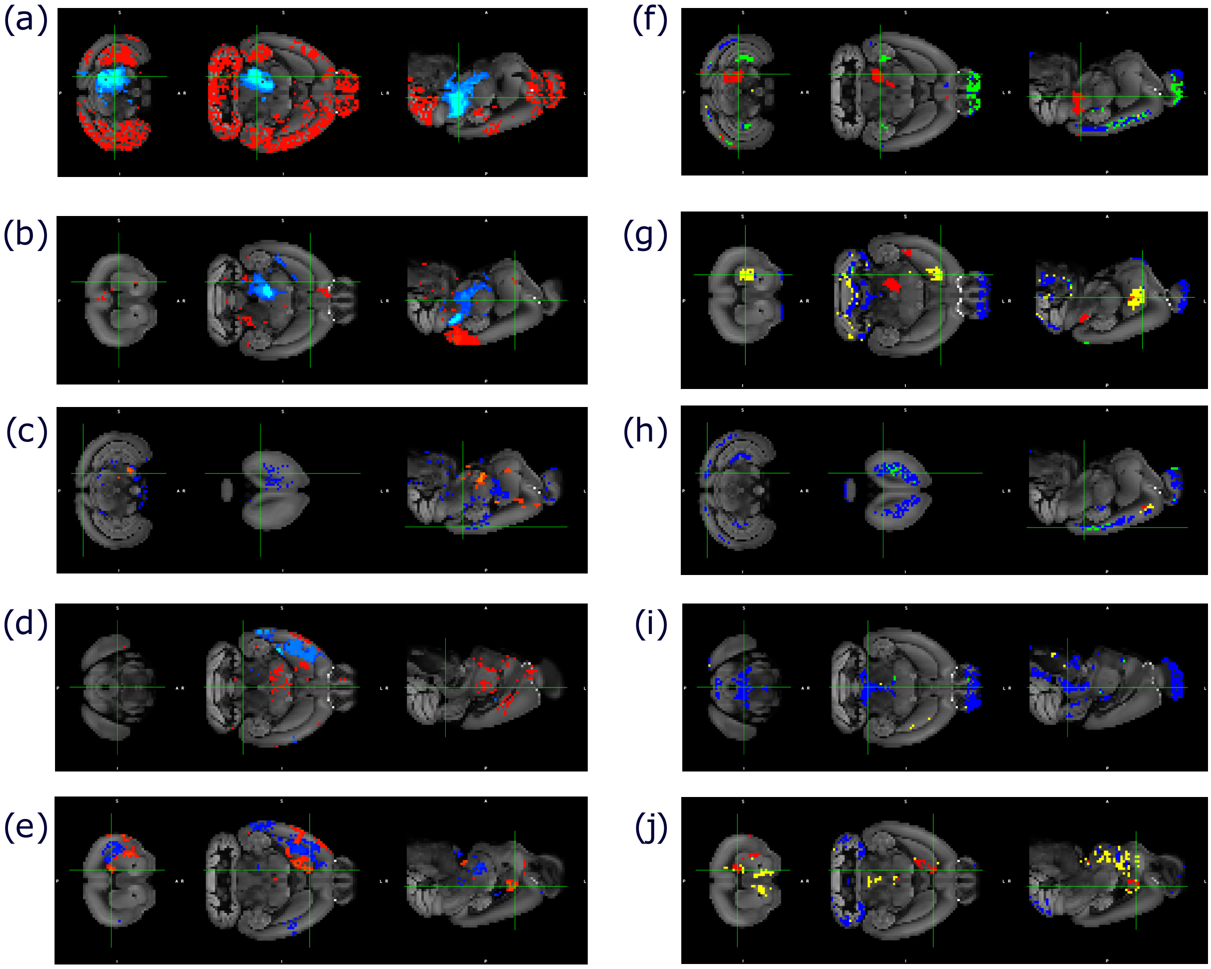

Fig. S4: Spatial maps for components of interest from the mrn and cp injection groups. (a-f) tract-tracing maps. (g-l) color-coded spatial maps. The thresholding and color convention for the spatial map visualizations is similar to the one used in Main figure 2 and a detailed description of the color-coding convention can be found in section S 1.6. In each column we show components 0 and 4 from mrn and components 0, 3 and 5 from cp, in the order of reference.

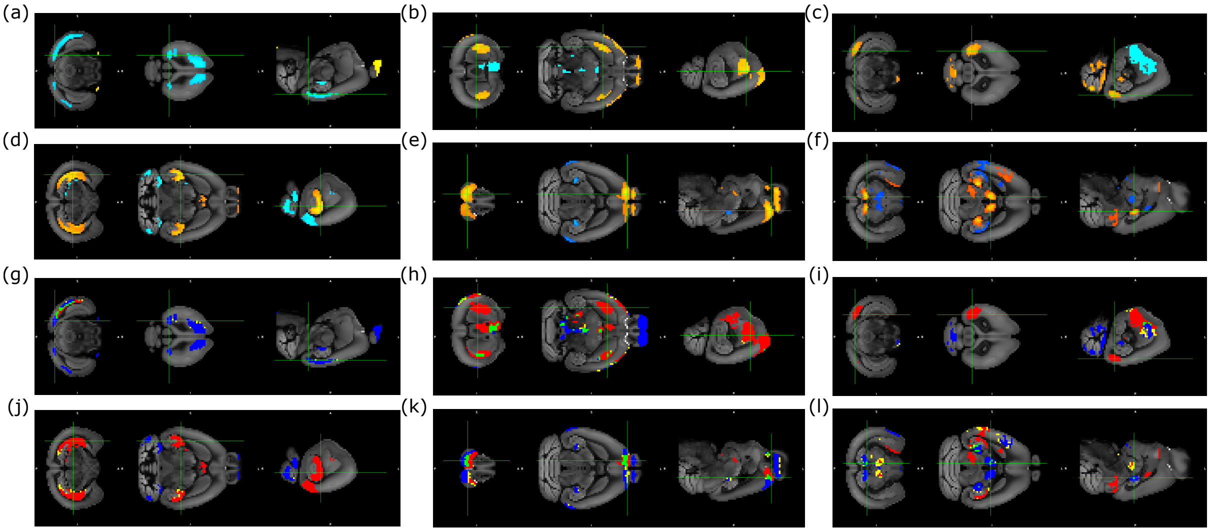

Fig. S5: Spatial maps for components of interest from the global analysis. (a-f) shared spatial maps. (g-l) color-coded spatial maps. The thresholding and color convention for the spatial map visualizations is similar to the one used in Main figure 2 and a detailed description of the color-coding convention can be found in section S 1.6. In each column we show components 0, 1, 2, 5, 10 and 23 in the order of reference.

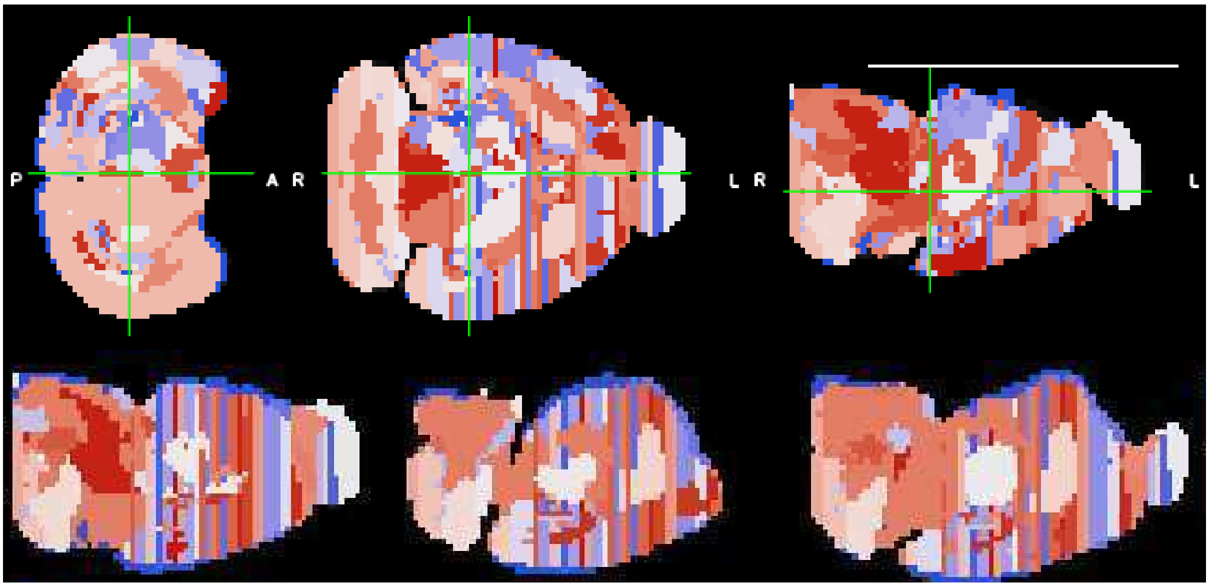

Fig. S6: Spatial map delineating parcellation of voxels to brain areas according to the approach shown in section S 1.9. The normalized mutual information (NMI) between this parcellation and the one provided by the ARA (Wang et al., 2020) is 0.48.

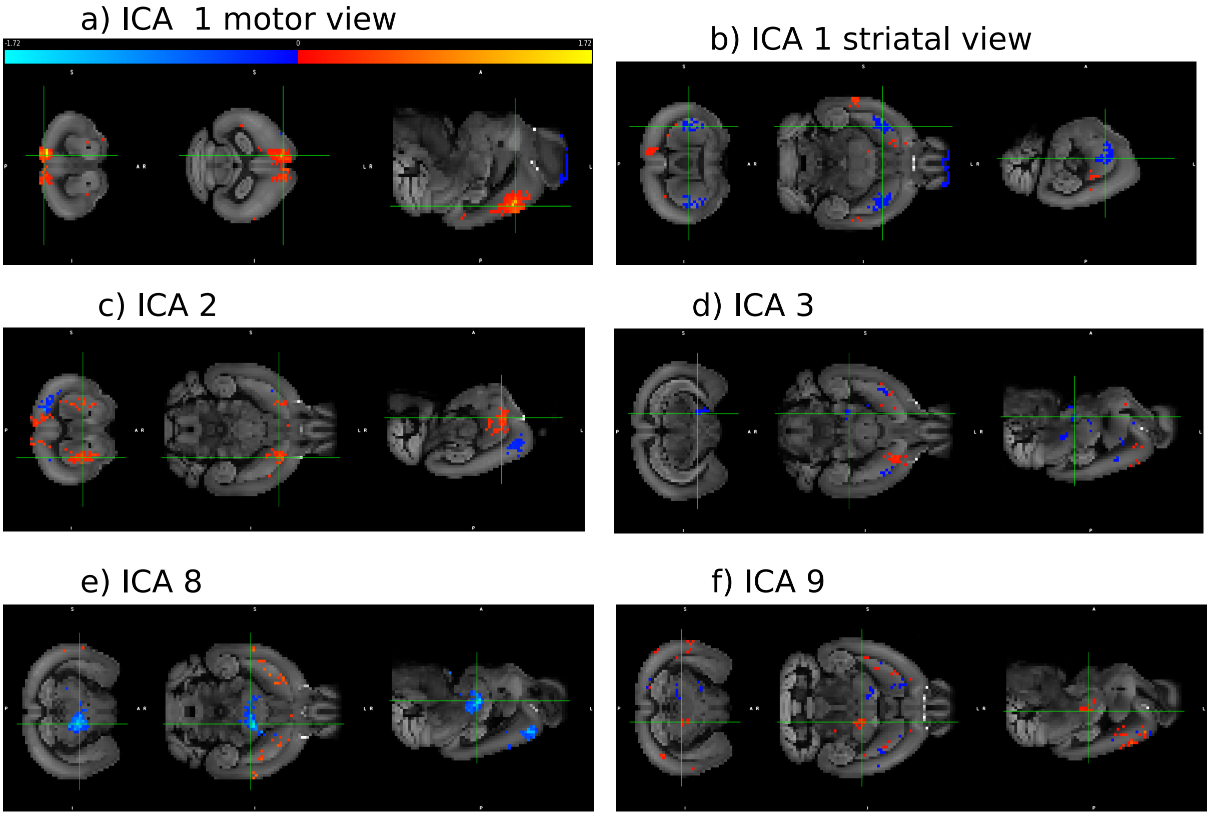

Fig. S7: Shared spatial maps for components of interest from the *MO* group, comprising a fusion of gene expression, projection density and single-neuron long-range reconstructions from the motor cortex. The thresholding details and color convention for the spatial maps are the same as the ones used in Main figure 2. a-b: ICA 1, views highlighting the motor areas (b) and the striatal areas (b). c-f: ICAs 2, 3, 8, 9 in the order of reference.

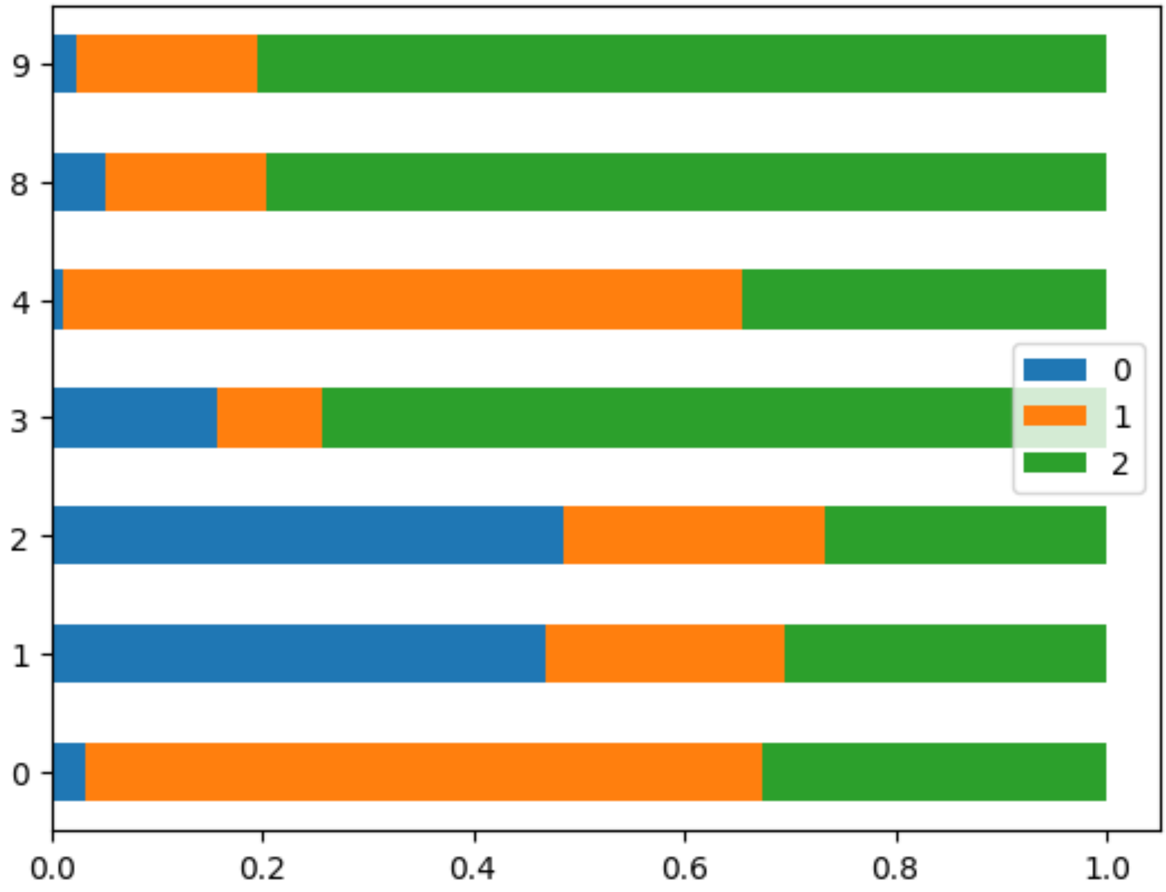

Fig. S8: Modality contributions for the components of interest from the *MO* group. The components shown are 0, 1, 2, 3, 4, 8, 9 in the order of reference. x-axis: contribution percentage. y-axis: components (starting from 0). The blue color represents the gene expression data, the orange color represents the projection density data and the green color represents the single-neuron data.

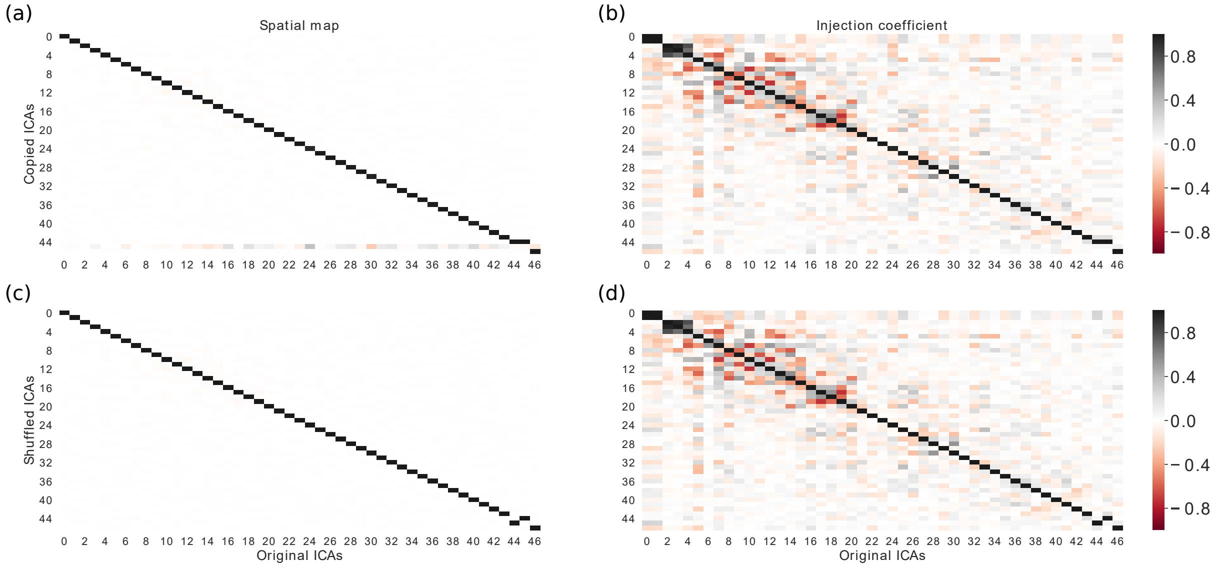

Fig. S9: Exemplar correlation matrices between two original arrays and their predicted form after two manipulations. The matrices correspond to the *vis* spatial map (H) (a,c) and the vis projection coefficients (X^1) (b,d). a,b) copying the expression-coefficients of all genes from component 44 to component 45. c,d) shuffling the expression-coefficients of all genes between components 44 and 45. Left panels: correlation matrix between the original and predicted form of the spatial map. Right panels: correlation matrix between the original and predicted form of the projection coefficients. The correlation was estimated using Pearson's rho.

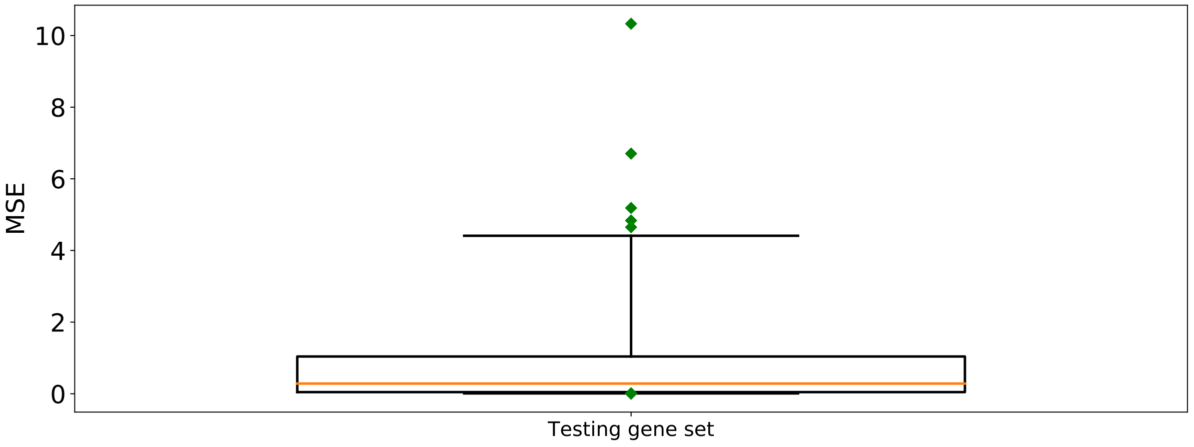

Fig. S10: Reconstruction accuracy of the Linked ICA model for a number of testing samples. y-axis: MSE scores. x-axis: testing set comprised of genes that were not included in the vis sub group analysis. orange line: mean. box: 25-75 percentiles. bottom/top lines: 5-95 percentiles. green points: outliers exceeding the 5-95 percentiles. Each MSE point represents the performance of the model in reconstructing the spatial pattern of a particular gene.

3 Supplemental Tables

Table S1: Enrichment-analysis-based annotations for gene subsets belonging to the vis injection group, which are related to neuronal and synaptic cellular components and metabolic pathways. (From left to right, top to bottom) gene subsets of the vis injection group for components 0,2,4,5,7 and 8 respectively. The gene subsets for each component have been selected based on high coefficient variance. The *KEGG* and *Org.Mm.eg.db* databases have been accessed for finding annotations with significant enrichments, by applying the hypergeometric test and false discovery rate, respectively ($p < 3.8 \times 10^{-5}$ for the *Org.Mm.eg.db* analysis). Due to the scarcity of the KEGG enrichment results, a number of KEGG-related annotations have been accepted based on a less strict cutoff value ($p < 0.001$).

	Term	Pvalue	Database	ICA
0	somatodendritic compartment	$< 3.8 \times 10^{-5}$	Org.Mm.eg CC	vis 0
1	neuron projection	$< 3.8 \times 10^{-5}$	Org.Mm.eg CC	vis 0
2	cell part	$< 3.8 \times 10^{-5}$	Org.Mm.eg CC	vis 0
3	cell	$< 3.8 \times 10^{-5}$	Org.Mm.eg CC	vis 0
4	axon	$< 3.8 \times 10^{-5}$	Org.Mm.eg CC	vis 0
5	neuron part	$< 3.8 \times 10^{-5}$	Org.Mm.eg CC	vis 0
6	plasma membrane bounded cell projection	$< 3.8 \times 10^{-5}$	Org.Mm.eg CC	vis 0
7	cell projection	$< 3.8 \times 10^{-5}$	Org.Mm.eg CC	vis 0
8	synapse	$< 3.8 \times 10^{-5}$	Org.Mm.eg CC	vis 0
9	cell surface	$< 3.8 \times 10^{-5}$	Org.Mm.eg CC	vis 0
10	synapse part	$< 3.8 \times 10^{-5}$	Org.Mm.eg CC	vis 0
11	multicellular organism development	$< 3.8 \times 10^{-5}$	Org.Mm.eg BP	vis 0
12	generation of neurons	$< 3.8 \times 10^{-5}$	Org.Mm.eg BP	vis 0
13	neurogenesis	$< 3.8 \times 10^{-5}$	Org.Mm.eg BP	vis 0
14	neuron differentiation	$< 3.8 \times 10^{-5}$	Org.Mm.eg BP	vis 0
15	neuron projection development	$< 3.8 \times 10^{-5}$	Org.Mm.eg BP	vis 0
16	multicellular organismal process	$< 3.8 \times 10^{-5}$	Org.Mm.eg BP	vis 0
17	cell development	$< 3.8 \times 10^{-5}$	Org.Mm.eg BP	vis 0
18	neuron development	$< 3.8 \times 10^{-5}$	Org.Mm.eg BP	vis 0
19	cell differentiation	$< 3.8 \times 10^{-5}$	Org.Mm.eg BP	vis 0

	Term	Pvalue	Database	ICA
0	cell part	$< 3.8 \times 10^{-5}$	Org.Mm.eg CC	vis 2
1	cell	$< 3.8 \times 10^{-5}$	Org.Mm.eg CC	vis 2
2	cell periphery	$< 3.8 \times 10^{-5}$	Org.Mm.eg CC	vis 2
3	cellular potassium ion transport	$< 3.8 \times 10^{-5}$	Org.Mm.eg BP	vis 2
4	cellular developmental process	$< 3.8 \times 10^{-5}$	Org.Mm.eg BP	vis 2
5	cellular process	$< 3.8 \times 10^{-5}$	Org.Mm.eg BP	vis 2
6	cell differentiation	$< 3.8 \times 10^{-5}$	Org.Mm.eg BP	vis 2

	Term	Pvalue	Database	ICA
0	cell part	$< 3.8 \times 10^{-5}$	Org.Mm.eg CC	vis 4
1	cell	$< 3.8 \times 10^{-5}$	Org.Mm.eg CC	vis 4
2	regulation of cell communication	$< 3.8 \times 10^{-5}$	Org.Mm.eg BP	vis 4
3	regulation of cellular process	$< 3.8 \times 10^{-5}$	Org.Mm.eg BP	vis 4
4	regulation of multicellular organismal process	$< 3.8 \times 10^{-5}$	Org.Mm.eg BP	vis 4
5	generation of neurons	$< 3.8 \times 10^{-5}$	Org.Mm.eg BP	vis 4
6	cell communication	$< 3.8 \times 10^{-5}$	Org.Mm.eg BP	vis 4
7	Glutamatergic synapse	0.0003	KEGG	vis 4

	Term	Pvalue	Database	ICA
0	dendritic spine	$< 3.8 \times 10^{-5}$	Org.Mm.eg CC	vis 5
1	neuron spine	$< 3.8 \times 10^{-5}$	Org.Mm.eg CC	vis 5
2	cell part	$< 3.8 \times 10^{-5}$	Org.Mm.eg CC	vis 5
3	cell	$< 3.8 \times 10^{-5}$	Org.Mm.eg CC	vis 5
4	cell periphery	$< 3.8 \times 10^{-5}$	Org.Mm.eg CC	vis 5
5	intracellular part	$< 3.8 \times 10^{-5}$	Org.Mm.eg CC	vis 5
6	intracellular	$< 3.8 \times 10^{-5}$	Org.Mm.eg CC	vis 5
7	cell junction	$< 3.8 \times 10^{-5}$	Org.Mm.eg CC	vis 5
8	plasma membrane bounded cell projection	$< 3.8 \times 10^{-5}$	Org.Mm.eg CC	vis 5
9	Endocrine and other factor-regulated calcium reabsorption	0.0010	KEGG	vis 5

	Term	Pvalue	Database	ICA
0	integral component of synaptic membrane	$< 3.8 \times 10^{-5}$	Org.Mm.eg CC	vis 7
1	integral component of postsynaptic membrane	$< 3.8 \times 10^{-5}$	Org.Mm.eg CC	vis 7
2	intrinsic component of synaptic membrane	$< 3.8 \times 10^{-5}$	Org.Mm.eg CC	vis 7
3	intrinsic component of postsynaptic membrane	$< 3.8 \times 10^{-5}$	Org.Mm.eg CC	vis 7
4	integral component of postsynaptic specialization membrane	$< 3.8 \times 10^{-5}$	Org.Mm.eg CC	vis 7
5	intrinsic component of postsynaptic specialization membrane	$< 3.8 \times 10^{-5}$	Org.Mm.eg CC	vis 7
6	postsynapse	$< 3.8 \times 10^{-5}$	Org.Mm.eg CC	vis 7
7	postsynaptic membrane	$< 3.8 \times 10^{-5}$	Org.Mm.eg CC	vis 7
8	postsynaptic specialization membrane	$< 3.8 \times 10^{-5}$	Org.Mm.eg CC	vis 7
9	synaptic membrane	$< 3.8 \times 10^{-5}$	Org.Mm.eg CC	vis 7
10	synapse part	$< 3.8 \times 10^{-5}$	Org.Mm.eg CC	vis 7
11	cell periphery	$< 3.8 \times 10^{-5}$	Org.Mm.eg CC	vis 7
12	neuronal cell body	$< 3.8 \times 10^{-5}$	Org.Mm.eg CC	vis 7
13	dendrite	$< 3.8 \times 10^{-5}$	Org.Mm.eg CC	vis 7
14	dendritic tree	$< 3.8 \times 10^{-5}$	Org.Mm.eg CC	vis 7
15	somatodendritic compartment	$< 3.8 \times 10^{-5}$	Org.Mm.eg CC	vis 7
16	synapse	$< 3.8 \times 10^{-5}$	Org.Mm.eg CC	vis 7
17	cell body	$< 3.8 \times 10^{-5}$	Org.Mm.eg CC	vis 7
18	integral component of postsynaptic density membrane	$< 3.8 \times 10^{-5}$	Org.Mm.eg CC	vis 7
19	neuron to neuron synapse	$< 3.8 \times 10^{-5}$	Org.Mm.eg CC	vis 7
20	cell projection part	$< 3.8 \times 10^{-5}$	Org.Mm.eg CC	vis 7
21	plasma membrane bounded cell projection part	$< 3.8 \times 10^{-5}$	Org.Mm.eg CC	vis 7
22	neuron part	$< 3.8 \times 10^{-5}$	Org.Mm.eg CC	vis 7
23	postsynaptic specialization	$< 3.8 \times 10^{-5}$	Org.Mm.eg CC	vis 7
24	intrinsic component of postsynaptic density membrane	$< 3.8 \times 10^{-5}$	Org.Mm.eg CC	vis 7
25	cell projection	$< 3.8 \times 10^{-5}$	Org.Mm.eg CC	vis 7
26	postsynaptic density membrane	$< 3.8 \times 10^{-5}$	Org.Mm.eg CC	vis 7
27	neuron projection	$< 3.8 \times 10^{-5}$	Org.Mm.eg CC	vis 7
28	postsynaptic density	$< 3.8 \times 10^{-5}$	Org.Mm.eg CC	vis 7
29	asymmetric synapse	$< 3.8 \times 10^{-5}$	Org.Mm.eg CC	vis 7
30	plasma membrane bounded cell projection	$< 3.8 \times 10^{-5}$	Org.Mm.eg CC	vis 7
31	cell part	$< 3.8 \times 10^{-5}$	Org.Mm.eg CC	vis 7
32	cell	$< 3.8 \times 10^{-5}$	Org.Mm.eg CC	vis 7
33	multicellular organism development	$< 3.8 \times 10^{-5}$	Org.Mm.eg BP	vis 7
34	neuron differentiation	$< 3.8 \times 10^{-5}$	Org.Mm.eg BP	vis 7
35	generation of neurons	$< 3.8 \times 10^{-5}$	Org.Mm.eg BP	vis 7
36	regulation of multicellular organismal process	$< 3.8 \times 10^{-5}$	Org.Mm.eg BP	vis 7
37	neurogenesis	$< 3.8 \times 10^{-5}$	Org.Mm.eg BP	vis 7
38	regulation of multicellular organismal development	$< 3.8 \times 10^{-5}$	Org.Mm.eg BP	vis 7
39	regulation of dendrite morphogenesis	$< 3.8 \times 10^{-5}$	Org.Mm.eg BP	vis 7
40	cell development	$< 3.8 \times 10^{-5}$	Org.Mm.eg BP	vis 7
41	negative regulation of multicellular organismal process	$< 3.8 \times 10^{-5}$	Org.Mm.eg BP	vis 7
42	multicellular organismal process	$< 3.8 \times 10^{-5}$	Org.Mm.eg BP	vis 7

	Term	Pvalue	Database	ICA
0	cell part	$< 3.8 \times 10^{-5}$	Org.Mm.eg CC	vis 8
1	cell	$< 3.8 \times 10^{-5}$	Org.Mm.eg CC	vis 8
2	intracellular part	$< 3.8 \times 10^{-5}$	Org.Mm.eg CC	vis 8
3	intracellular	$< 3.8 \times 10^{-5}$	Org.Mm.eg CC	vis 8
4	intracellular organelle	$< 3.8 \times 10^{-5}$	Org.Mm.eg CC	vis 8
5	cellular response to ammonium ion	$< 3.8 \times 10^{-5}$	Org.Mm.eg BP	vis 8
6	cellular process	$< 3.8 \times 10^{-5}$	Org.Mm.eg BP	vis 8
7	signal transduction involved in cellular response	$< 3.8 \times 10^{-5}$	Org.Mm.eg BP	vis 8
8	cellular response to acetylcholine	$< 3.8 \times 10^{-5}$	Org.Mm.eg BP	vis 8
9	Cardiac muscle contraction	0.0003	KEGG	vis 8

Table S2: Enrichment-analysis-based annotations for gene subsets belonging to the mrn and cp injection groups, which are related to neuronal and synaptic cellular components and metabolic pathways. (From left to right, top to bottom) gene subsets of the cp injection group for components 0, 3 and 5 respectively, followed by gene subsets of the mrn injection group for components 0 and 4 respectively. The gene subsets for each component have been selected based on high coefficient variance. The *KEGG* and *Org.Mm.eg.db* databases have been accessed for finding annotations of significant enrichments, by applying the hypergeometric test and false discovery rate procedure, respectively ($p < 3.8 \times 10^{-5}$ for the *Org.Mm.eg.db* analysis). Due to the scarcity of the KEGG enrichment results, a number of KEGG-related annotations have been accepted based on a less strict cutoff value ($p < 0.001$).

	Term	Pvalue	Database	ICA
0	somatodendritic compartment	$< 3.8 \times 10^{-5}$	Org.Mm.eg CC	cp 0
1	neuron projection	$< 3.8 \times 10^{-5}$	Org.Mm.eg CC	cp 0
2	neuron part	$< 3.8 \times 10^{-5}$	Org.Mm.eg CC	cp 0
3	axon	$< 3.8 \times 10^{-5}$	Org.Mm.eg CC	cp 0
4	plasma membrane bounded cell projection	$< 3.8 \times 10^{-5}$	Org.Mm.eg CC	cp 0
5	cell projection	$< 3.8 \times 10^{-5}$	Org.Mm.eg CC	cp 0
6	cell part	$< 3.8 \times 10^{-5}$	Org.Mm.eg CC	cp 0
7	cell	$< 3.8 \times 10^{-5}$	Org.Mm.eg CC	cp 0
8	cell body	$< 3.8 \times 10^{-5}$	Org.Mm.eg CC	cp 0
9	neuronal cell body	$< 3.8 \times 10^{-5}$	Org.Mm.eg CC	cp 0
10	dendrite	$< 3.8 \times 10^{-5}$	Org.Mm.eg CC	cp 0
11	dendritic tree	$< 3.8 \times 10^{-5}$	Org.Mm.eg CC	cp 0
12	synapse	$< 3.8 \times 10^{-5}$	Org.Mm.eg CC	cp 0
13	cell periphery	$< 3.8 \times 10^{-5}$	Org.Mm.eg CC	cp 0
14	cell surface	$< 3.8 \times 10^{-5}$	Org.Mm.eg CC	cp 0
15	axon part	$< 3.8 \times 10^{-5}$	Org.Mm.eg CC	cp 0
16	synapse part	$< 3.8 \times 10^{-5}$	Org.Mm.eg CC	cp 0
17	generation of neurons	$< 3.8 \times 10^{-5}$	Org.Mm.eg BP	cp 0
18	neurogenesis	$< 3.8 \times 10^{-5}$	Org.Mm.eg BP	cp 0
19	neuron differentiation	$< 3.8 \times 10^{-5}$	Org.Mm.eg BP	cp 0
20	multicellular organism development	$< 3.8 \times 10^{-5}$	Org.Mm.eg BP	cp 0
21	neuron projection development	$< 3.8 \times 10^{-5}$	Org.Mm.eg BP	cp 0
22	neuron development	$< 3.8 \times 10^{-5}$	Org.Mm.eg BP	cp 0
23	plasma membrane bounded cell projection organization	$< 3.8 \times 10^{-5}$	Org.Mm.eg BP	cp 0
24	cell development	$< 3.8 \times 10^{-5}$	Org.Mm.eg BP	cp 0
25	dendrite development	$< 3.8 \times 10^{-5}$	Org.Mm.eg BP	cp 0
26	cell projection organization	$< 3.8 \times 10^{-5}$	Org.Mm.eg BP	cp 0
27	cell motility	$< 3.8 \times 10^{-5}$	Org.Mm.eg BP	cp 0
28	localization of cell	$< 3.8 \times 10^{-5}$	Org.Mm.eg BP	cp 0
29	cell migration	$< 3.8 \times 10^{-5}$	Org.Mm.eg BP	cp 0

	Term	Pvalue	Database	ICA
0	cell part	$< 3.8 \times 10^{-5}$	Org.Mm.eg CC	cp 3
1	cell	$< 3.8 \times 10^{-5}$	Org.Mm.eg CC	cp 3
2	cell periphery	$< 3.8 \times 10^{-5}$	Org.Mm.eg CC	cp 3
3	synapse	$< 3.8 \times 10^{-5}$	Org.Mm.eg CC	cp 3
4	synapse part	$< 3.8 \times 10^{-5}$	Org.Mm.eg CC	cp 3
5	cellular developmental process	$< 3.8 \times 10^{-5}$	Org.Mm.eg BP	cp 3
6	cell differentiation	$< 3.8 \times 10^{-5}$	Org.Mm.eg BP	cp 3
7	cellular process	$< 3.8 \times 10^{-5}$	Org.Mm.eg BP	cp 3
8	multicellular organism development	$< 3.8 \times 10^{-5}$	Org.Mm.eg BP	cp 3
9	generation of neurons	$< 3.8 \times 10^{-5}$	Org.Mm.eg BP	cp 3
10	neuron differentiation	$< 3.8 \times 10^{-5}$	Org.Mm.eg BP	cp 3
11	cell development	$< 3.8 \times 10^{-5}$	Org.Mm.eg BP	cp 3
12	neurogenesis	$< 3.8 \times 10^{-5}$	Org.Mm.eg BP	cp 3
13	multicellular organismal process	$< 3.8 \times 10^{-5}$	Org.Mm.eg BP	cp 3

0	cell part	$< 3.8 \times 10^{-5}$	Org.Mm.eg CC	cp 5
1	cell	$< 3.8 \times 10^{-5}$	Org.Mm.eg CC	cp 5
2	Glutamatergic synapse	0.0003	KEGG	cp 5
3	Retrograde endocannabinoid signaling	0.0007	KEGG	cp 5

	Term	Pvalue	Database	ICA
0	somatodendritic compartment	$< 3.8 \times 10^{-5}$	Org.Mm.eg CC	mrn 0
1	neuron projection	$< 3.8 \times 10^{-5}$	Org.Mm.eg CC	mrn 0
2	neuron part	$< 3.8 \times 10^{-5}$	Org.Mm.eg CC	mrn 0
3	plasma membrane bounded cell projection	$< 3.8 \times 10^{-5}$	Org.Mm.eg CC	mrn 0
4	dendrite	$< 3.8 \times 10^{-5}$	Org.Mm.eg CC	mrn 0
5	dendritic tree	$< 3.8 \times 10^{-5}$	Org.Mm.eg CC	mrn 0
6	cell part	$< 3.8 \times 10^{-5}$	Org.Mm.eg CC	mrn 0
7	cell	$< 3.8 \times 10^{-5}$	Org.Mm.eg CC	mrn 0
8	synapse	$< 3.8 \times 10^{-5}$	Org.Mm.eg CC	mrn 0
9	cell body	$< 3.8 \times 10^{-5}$	Org.Mm.eg CC	mrn 0
10	cell projection	$< 3.8 \times 10^{-5}$	Org.Mm.eg CC	mrn 0
11	cell periphery	$< 3.8 \times 10^{-5}$	Org.Mm.eg CC	mrn 0
12	synapse part	$< 3.8 \times 10^{-5}$	Org.Mm.eg CC	mrn 0
13	axon	$< 3.8 \times 10^{-5}$	Org.Mm.eg CC	mrn 0
14	neuronal cell body	$< 3.8 \times 10^{-5}$	Org.Mm.eg CC	mrn 0
15	postsynapse	$< 3.8 \times 10^{-5}$	Org.Mm.eg CC	mrn 0
16	dendritic spine	$< 3.8 \times 10^{-5}$	Org.Mm.eg CC	mrn 0
17	neuron spine	$< 3.8 \times 10^{-5}$	Org.Mm.eg CC	mrn 0
18	cell surface	$< 3.8 \times 10^{-5}$	Org.Mm.eg CC	mrn 0
19	dendrite development	$< 3.8 \times 10^{-5}$	Org.Mm.eg BP	mrn 0
20	generation of neurons	$< 3.8 \times 10^{-5}$	Org.Mm.eg BP	mrn 0
21	neurogenesis	$< 3.8 \times 10^{-5}$	Org.Mm.eg BP	mrn 0
22	cell motility	$< 3.8 \times 10^{-5}$	Org.Mm.eg BP	mrn 0
23	localization of cell	$< 3.8 \times 10^{-5}$	Org.Mm.eg BP	mrn 0
24	neuron projection development	$< 3.8 \times 10^{-5}$	Org.Mm.eg BP	mrn 0
25	multicellular organism development	$< 3.8 \times 10^{-5}$	Org.Mm.eg BP	mrn 0
26	cell migration	$< 3.8 \times 10^{-5}$	Org.Mm.eg BP	mrn 0
27	neuron differentiation	$< 3.8 \times 10^{-5}$	Org.Mm.eg BP	mrn 0
28	regulation of cell differentiation	$< 3.8 \times 10^{-5}$	Org.Mm.eg BP	mrn 0
29	neuron development	$< 3.8 \times 10^{-5}$	Org.Mm.eg BP	mrn 0
30	regulation of multicellular organismal development	$< 3.8 \times 10^{-5}$	Org.Mm.eg BP	mrn 0
31	movement of cell or subcellular component	$< 3.8 \times 10^{-5}$	Org.Mm.eg BP	mrn 0
32	cell differentiation	$< 3.8 \times 10^{-5}$	Org.Mm.eg BP	mrn 0
33	neuron migration	$< 3.8 \times 10^{-5}$	Org.Mm.eg BP	mrn 0
34	plasma membrane bounded cell projection organization	$< 3.8 \times 10^{-5}$	Org.Mm.eg BP	mrn 0
35	neuron projection morphogenesis	$< 3.8 \times 10^{-5}$	Org.Mm.eg BP	mrn 0
36	cell development	$< 3.8 \times 10^{-5}$	Org.Mm.eg BP	mrn 0
37	plasma membrane bounded cell projection morphogenesis	$< 3.8 \times 10^{-5}$	Org.Mm.eg BP	mrn 0
38	cell projection morphogenesis	$< 3.8 \times 10^{-5}$	Org.Mm.eg BP	mrn 0
39	cellular developmental process	$< 3.8 \times 10^{-5}$	Org.Mm.eg BP	mrn 0
40	cell projection organization	$< 3.8 \times 10^{-5}$	Org.Mm.eg BP	mrn 0
41	positive regulation of cell death	$< 3.8 \times 10^{-5}$	Org.Mm.eg BP	mrn 0
42	cell part morphogenesis	$< 3.8 \times 10^{-5}$	Org.Mm.eg BP	mrn 0
43	cell morphogenesis	$< 3.8 \times 10^{-5}$	Org.Mm.eg BP	mrn 0
44	positive regulation of cell projection organization	$< 3.8 \times 10^{-5}$	Org.Mm.eg BP	mrn 0
45	positive regulation of cell differentiation	$< 3.8 \times 10^{-5}$	Org.Mm.eg BP	mrn 0
46	cell morphogenesis involved in neuron differentiation	$< 3.8 \times 10^{-5}$	Org.Mm.eg BP	mrn 0
47	regulation of neurogenesis	$< 3.8 \times 10^{-5}$	Org.Mm.eg BP	mrn 0
48	Phospholipase D signaling pathway	0.0002	KEGG	mrn 0

	Term	Pvalue	Database	ICA
0	cell part	$< 3.8 \times 10^{-5}$	Org.Mm.eg CC	mrn 4
1	cell	$< 3.8 \times 10^{-5}$	Org.Mm.eg CC	mrn 4
2	dendrite	$< 3.8 \times 10^{-5}$	Org.Mm.eg CC	mrn 4
3	dendritic tree	$< 3.8 \times 10^{-5}$	Org.Mm.eg CC	mrn 4
4	neuron part	$< 3.8 \times 10^{-5}$	Org.Mm.eg CC	mrn 4
5	somatodendritic compartment	$< 3.8 \times 10^{-5}$	Org.Mm.eg CC	mrn 4
6	neuron projection	$< 3.8 \times 10^{-5}$	Org.Mm.eg CC	mrn 4
7	synapse	$< 3.8 \times 10^{-5}$	Org.Mm.eg CC	mrn 4
8	cellular process	$< 3.8 \times 10^{-5}$	Org.Mm.eg BP	mrn 4
9	cell differentiation	$< 3.8 \times 10^{-5}$	Org.Mm.eg BP	mrn 4
10	Cholinergic synapse	0.0002	KEGG	mrn 4

Table S3: a) Gene markers from components of interest, that have been cell-type enriched based on lists from (Tasic et al., 2018). a) vis injection group, b) mrn and cp injection groups. c) global analysis.

(a)

	Gene Markers	Pathway	ICA	Gene Markers	Pathway	ICA	Gene Markers	Pathway	ICA	Gene Markers	Pathway	ICA	Gene Markers	Pathway	ICA	Gene Markers	Pathway	ICA
0	Ppapdc1a	GABAergic	vis 0	Sema5a	GABAergic	vis 2	Sema5a	GABAergic	vis 4	Syndig1	GABAergic	vis 5	Syndig1	GABAergic	vis 7	Syndig1	GABAergic	vis 8
1	Ptprk	GABAergic	vis 0	Ppapdc1a	GABAergic	vis 2	Rspo1	GABAergic	vis 4	Sema5a	GABAergic	vis 5	Sema5a	GABAergic	vis 7	Sema5a	GABAergic	vis 8
2	Sln	GABAergic	vis 0	Ptprk	GABAergic	vis 2	Ppapdc1a	GABAergic	vis 4	Rspo1	GABAergic	vis 5	Rspo1	GABAergic	vis 7	Rspo1	GABAergic	vis 8
3	Inpp4b	Glutamatergic	vis 0	Sln	GABAergic	vis 2	Ptprk	GABAergic	vis 4	Ppapdc1a	GABAergic	vis 5	Chrna3	GABAergic	vis 7	Chrna3	GABAergic	vis 8
4	Adssl1	Glutamatergic	vis 0	Grm1	GABAergic	vis 2	Sln	GABAergic	vis 4	Ptprk	GABAergic	vis 5	Ppapdc1a	GABAergic	vis 7	Ppapdc1a	GABAergic	vis 8
5	Lhx2	Glutamatergic	vis 0	Lypd1	Glutamatergic	vis 2	Grm1	GABAergic	vis 4	Chrnb3	GABAergic	vis 5	Ptprk	GABAergic	vis 7	Il1rapl2	GABAergic	vis 8
6	Unc5d	Glutamatergic	vis 0	Inpp4b	Glutamatergic	vis 2	Tmie	Glutamatergic	vis 4	Bche	GABAergic	vis 5	Chrnb3	GABAergic	vis 7	Fam212b	GABAergic	vis 8
7	Aqp4	Non-Neuronal	vis 0	Adssl1	Glutamatergic	vis 2	Lypd1	Glutamatergic	vis 4	Sln	GABAergic	vis 5	Bche	GABAergic	vis 7	Ptprk	GABAergic	vis 8
8	Sdc3	Non-Neuronal	vis 0	Chrna6	Glutamatergic	vis 2	Inpp4b	Glutamatergic	vis 4	Grm1	GABAergic	vis 5	Sln	GABAergic	vis 7	Chrnb3	GABAergic	vis 8
9	Siglech	Non-Neuronal	vis 0	Lhx2	Glutamatergic	vis 2	Adssl1	Glutamatergic	vis 4	Tmie	Glutamatergic	vis 5	Penk	GABAergic	vis 7	Bche	GABAergic	vis 8
10	NaN	NaN	NaN	Tshz2	Glutamatergic	vis 2	Chrna6	Glutamatergic	vis 4	Lypd1	Glutamatergic	vis 5	Grm1	GABAergic	vis 7	Sln	GABAergic	vis 8
11	NaN	NaN	NaN	Unc5d	Glutamatergic	vis 2	Lhx2	Glutamatergic	vis 4	Inpp4b	Glutamatergic	vis 5	Tmie	Glutamatergic	vis 7	Penk	GABAergic	vis 8
12	NaN	NaN	NaN	Aqp4	Non-Neuronal	vis 2	Tshz2	Glutamatergic	vis 4	Adssl1	Glutamatergic	vis 5	Lypd1	Glutamatergic	vis 7	Grm1	GABAergic	vis 8
13	NaN	NaN	NaN	Sdc3	Non-Neuronal	vis 2	Unc5d	Glutamatergic	vis 4	Chrna6	Glutamatergic	vis 5	Inpp4b	Glutamatergic	vis 7	Whrn	Glutamatergic	vis 8
14	NaN	NaN	NaN	Olfm2	Non-Neuronal	vis 2	Aqp4	Non-Neuronal	vis 4	Lhx2	Glutamatergic	vis 5	Adssl1	Glutamatergic	vis 7	Tmie	Glutamatergic	vis 8
15	NaN	NaN	NaN	Siglech	Non-Neuronal	vis 2	Sdc3	Non-Neuronal	vis 4	Tshz2	Glutamatergic	vis 5	Cd24a	Glutamatergic	vis 7	Lypd1	Glutamatergic	vis 8
16	NaN	NaN	NaN	NaN	NaN	NaN	Olfm2	Non-Neuronal	vis 4	Unc5d	Glutamatergic	vis 5	Chrna6	Glutamatergic	vis 7	Inpp4b	Glutamatergic	vis 8
17	NaN	NaN	NaN	NaN	NaN	NaN	Siglech	Non-Neuronal	vis 4	Defb1	Glutamatergic	vis 5	Lhx2	Glutamatergic	vis 7	Adssl1	Glutamatergic	vis 8
18	NaN	NaN	NaN	NaN	NaN	NaN	NaN	NaN	NaN	Aqp4	Non-Neuronal	vis 5	Tshz2	Glutamatergic	vis 7	Cd24a	Glutamatergic	vis 8
19	NaN	NaN	NaN	NaN	NaN	NaN	NaN	NaN	NaN	Sox10	Non-Neuronal	vis 5	Tdo2	Glutamatergic	vis 7	Chrna6	Glutamatergic	vis 8
20	NaN	NaN	NaN	NaN	NaN	NaN	NaN	NaN	NaN	Sdc3	Non-Neuronal	vis 5	Unc5d	Glutamatergic	vis 7	Man1a	Glutamatergic	vis 8
21	NaN	NaN	NaN	NaN	NaN	NaN	NaN	NaN	NaN	Olfm2	Non-Neuronal	vis 5	Defb1	Glutamatergic	vis 7	Lhx2	Glutamatergic	vis 8
22	NaN	NaN	NaN	NaN	NaN	NaN	NaN	NaN	NaN	Siglech	Non-Neuronal	vis 5	Aqp4	Non-Neuronal	vis 7	Tshz2	Glutamatergic	vis 8
23	NaN	NaN	NaN	NaN	NaN	NaN	NaN	NaN	NaN	NaN	NaN	NaN	Sox10	Non-Neuronal	vis 7	Tdo2	Glutamatergic	vis 8
24	NaN	NaN	NaN	NaN	NaN	NaN	NaN	NaN	NaN	NaN	NaN	NaN	Sdc3	Non-Neuronal	vis 7	Unc5d	Glutamatergic	vis 8
25	NaN	NaN	NaN	NaN	NaN	NaN	NaN	NaN	NaN	NaN	NaN	NaN	Olfm2	Non-Neuronal	vis 7	Defb1	Glutamatergic	vis 8
26	NaN	NaN	NaN	NaN	NaN	NaN	NaN	NaN	NaN	NaN	NaN	NaN	Siglech	Non-Neuronal	vis 7	Lhx1	Glutamatergic	vis 8
27	NaN	NaN	NaN	NaN	NaN	NaN	NaN	NaN	NaN	NaN	NaN	NaN	NaN	NaN	NaN	Aqp4	Non-Neuronal	vis 8
28	NaN	NaN	NaN	NaN	NaN	NaN	NaN	NaN	NaN	NaN	NaN	NaN	NaN	NaN	NaN	Sox10	Non-Neuronal	vis 8
29	NaN	NaN	NaN	NaN	NaN	NaN	NaN	NaN	NaN	NaN	NaN	NaN	NaN	NaN	NaN	Sdc3	Non-Neuronal	vis 8
30	NaN	NaN	NaN	NaN	NaN	NaN	NaN	NaN	NaN	NaN	NaN	NaN	NaN	NaN	NaN	Olfm2	Non-Neuronal	vis 8
31	NaN	NaN	NaN	NaN	NaN	NaN	NaN	NaN	NaN	NaN	NaN	NaN	NaN	NaN	NaN	Siglech	Non-Neuronal	vis 8

(b)

	Gene Markers	Pathway	ICA	Gene Markers	Pathway	ICA	Gene Markers	Pathway	ICA	Gene Markers	Pathway	ICA	Gene Markers	Pathway	ICA
0	Syndig1	GABAergic	cp 0	Syndig1	GABAergic	cp 3	Sema5a	GABAergic	cp 5	Sema5a	GABAergic	mrn 0	Sema5a	GABAergic	mrn 4
1	Sema5a	GABAergic	cp 0	Sema5a	GABAergic	cp 3	Rspo1	GABAergic	cp 5	Rspo1	GABAergic	mrn 0	Rspo1	GABAergic	mrn 4
2	Rspo1	GABAergic	cp 0	Rspo1	GABAergic	cp 3	Chrna3	GABAergic	cp 5	Chrna3	GABAergic	mrn 0	Chrna3	GABAergic	mrn 4
3	Chrna3	GABAergic	cp 0	Chrna3	GABAergic	cp 3	Ppapdc1a	GABAergic	cp 5	Ppapdc1a	GABAergic	mrn 0	Ppapdc1a	GABAergic	mrn 4
4	Ppapdc1a	GABAergic	cp 0	Ppapdc1a	GABAergic	cp 3	Il1rapl2	GABAergic	cp 5	Il1rapl2	GABAergic	mrn 0	Il1rapl2	GABAergic	mrn 4
5	Il1rapl2	GABAergic	cp 0	Il1rapl2	GABAergic	cp 3	Fam212b	GABAergic	cp 5	Fam212b	GABAergic	mrn 0	Fam212b	GABAergic	mrn 4
6	Fam212b	GABAergic	cp 0	Fam212b	GABAergic	cp 3	Sln	GABAergic	cp 5	Sln	GABAergic	mrn 0	Sln	GABAergic	mrn 4
7	Chrnb3	GABAergic	cp 0	Chrnb3	GABAergic	cp 3	Penk	GABAergic	cp 5	Penk	GABAergic	mrn 0	Penk	GABAergic	mrn 4
8	Bche	GABAergic	cp 0	Bche	GABAergic	cp 3	Grm1	GABAergic	cp 5	Grm1	GABAergic	mrn 0	Grm1	GABAergic	mrn 4
9	Sln	GABAergic	cp 0	Sln	GABAergic	cp 3	Whrn	Glutamatergic	cp 5	Col25a1	GABAergic	mrn 0	Col25a1	GABAergic	mrn 4
10	Penk	GABAergic	cp 0	Penk	GABAergic	cp 3	Tmie	Glutamatergic	cp 5	Whrn	Glutamatergic	mrn 0	Whrn	Glutamatergic	mrn 4
11	Grm1	GABAergic	cp 0	Grm1	GABAergic	cp 3	Lypd1	Glutamatergic	cp 5	Tmie	Glutamatergic	mrn 0	Tmie	Glutamatergic	mrn 4
12	Whrn	Glutamatergic	cp 0	Whrn	Glutamatergic	cp 3	Inpp4b	Glutamatergic	cp 5	Lypd1	Glutamatergic	mrn 0	Lypd1	Glutamatergic	mrn 4
13	Tmie	Glutamatergic	cp 0	Tmie	Glutamatergic	cp 3	Adssl1	Glutamatergic	cp 5	Inpp4b	Glutamatergic	mrn 0	Inpp4b	Glutamatergic	mrn 4
14	Lypd1	Glutamatergic	cp 0	Lypd1	Glutamatergic	cp 3	Cd24a	Glutamatergic	cp 5	Adssl1	Glutamatergic	mrn 0	Adssl1	Glutamatergic	mrn 4
15	Inpp4b	Glutamatergic	cp 0	Inpp4b	Glutamatergic	cp 3	Chrna6	Glutamatergic	cp 5	Cd24a	Glutamatergic	mrn 0	Cd24a	Glutamatergic	mrn 4
16	Adssl1	Glutamatergic	cp 0	Adssl1	Glutamatergic	cp 3	Man1a	Glutamatergic	cp 5	Chrna6	Glutamatergic	mrn 0	Chrna6	Glutamatergic	mrn 4
17	Cd24a	Glutamatergic	cp 0	Cd24a	Glutamatergic	cp 3	Tshz2	Glutamatergic	cp 5	Man1a	Glutamatergic	mrn 0	Man1a	Glutamatergic	mrn 4
18	Chrna6	Glutamatergic	cp 0	Chrna6	Glutamatergic	cp 3	Tdo2	Glutamatergic	cp 5	Tshz2	Glutamatergic	mrn 0	Tshz2	Glutamatergic	mrn 4
19	Man1a	Glutamatergic	cp 0	Man1a	Glutamatergic	cp 3	Unc5d	Glutamatergic	cp 5	Tdo2	Glutamatergic	mrn 0	Tdo2	Glutamatergic	mrn 4
20	Tshz2	Glutamatergic	cp 0	Tshz2	Glutamatergic	cp 3	Lhx1	Glutamatergic	cp 5	Unc5d	Glutamatergic	mrn 0	Unc5d	Glutamatergic	mrn 4
21	Tdo2	Glutamatergic	cp 0	Tdo2	Glutamatergic	cp 3	Aqp4	Non-Neuronal	cp 5	Lhx1	Glutamatergic	mrn 0	Lhx1	Glutamatergic	mrn 4
22	Unc5d	Glutamatergic	cp 0	Unc5d	Glutamatergic	cp 3	Sdc3	Non-Neuronal	cp 5	Aqp4	Non-Neuronal	mrn 0	Aqp4	Non-Neuronal	mrn 4
23	Defb1	Glutamatergic	cp 0	Defb1	Glutamatergic	cp 3	Olfm2	Non-Neuronal	cp 5	Sdc3	Non-Neuronal	mrn 0	Sdc3	Non-Neuronal	mrn 4
24	Lhx1	Glutamatergic	cp 0	Lhx1	Glutamatergic	cp 3	Siglech	Non-Neuronal	cp 5	Olfm2	Non-Neuronal	mrn 0	Olfm2	Non-Neuronal	mrn 4
25	Aqp4	Non-Neuronal	cp 0	Aqp4	Non-Neuronal	cp 3	NaN	NaN	NaN	Siglech	Non-Neuronal	mrn 0	Siglech	Non-Neuronal	mrn 4
26	Sox10	Non-Neuronal	cp 0	Sox10	Non-Neuronal	cp 3	NaN	NaN	NaN	NaN	NaN	NaN	NaN	NaN	NaN
27	Sdc3	Non-Neuronal	cp 0	Sdc3	Non-Neuronal	cp 3	NaN	NaN	NaN	NaN	NaN	NaN	NaN	NaN	NaN
28	Olfm2	Non-Neuronal	cp 0	Olfm2	Non-Neuronal	cp 3	NaN	NaN	NaN	NaN	NaN	NaN	NaN	NaN	NaN
29	Siglech	Non-Neuronal	cp 0	Siglech	Non-Neuronal	cp 3	NaN	NaN	NaN	NaN	NaN	NaN	NaN	NaN	NaN

(c)

	Gene Markers	Pathway	ICA	Gene Markers	Pathway	ICA	Gene Markers	Pathway	ICA	Gene Markers	Pathway	ICA	Gene Markers	Pathway	ICA	Gene Markers	Pathway	ICA
0	Sema5a	GABAergic	Global 0	Sema5a	GABAergic	Global 1	Sema5a	GABAergic	Global 2	Sema5a	GABAergic	Global 5	Gprin3	GABAergic	Global 10	Krt73	GABAergic	Global 23
1	Rspo1	GABAergic	Global 0	Rspo1	GABAergic	Global 1	Rspo1	GABAergic	Global 2	Rspo1	GABAergic	Global 5	Mob3b	GABAergic	Global 10	Gprin3	GABAergic	Global 23
2	Chrna3	GABAergic	Global 0	Chrna3	GABAergic	Global 1	Chrna3	GABAergic	Global 2	Chrna3	GABAergic	Global 5	Sema5a	GABAergic	Global 10	Mob3b	GABAergic	Global 23
3	Ppapdc1a	GABAergic	Global 0	Mme	GABAergic	Global 1	Mme	GABAergic	Global 2	Mme	GABAergic	Global 5	Rspo1	GABAergic	Global 10	Sema5a	GABAergic	Global 23
4	Il1rapl2	GABAergic	Global 0	Ppapdc1a	GABAergic	Global 1	Ppapdc1a	GABAergic	Global 2	Ppapdc1a	GABAergic	Global 5	Chrna3	GABAergic	Global 10	Rspo1	GABAergic	Global 23
5	Fam212b	GABAergic	Global 0	Il1rapl2	GABAergic	Global 1	Il1rapl2	GABAergic	Global 2	Il1rapl2	GABAergic	Global 5	Mme	GABAergic	Global 10	Chrna3	GABAergic	Global 23
6	Sln	GABAergic	Global 0	Fam212b	GABAergic	Global 1	Fam212b	GABAergic	Global 2	Fam212b	GABAergic	Global 5	Ppapdc1a	GABAergic	Global 10	Mme	GABAergic	Global 23
7	Penk	GABAergic	Global 0	Sln	GABAergic	Global 1	Sln	GABAergic	Global 2	Sln	GABAergic	Global 5	Il1rapl2	GABAergic	Global 10	Ppapdc1a	GABAergic	Global 23
8	Grm1	GABAergic	Global 0	Penk	GABAergic	Global 1	Penk	GABAergic	Global 2	Penk	GABAergic	Global 5	Fam212b	GABAergic	Global 10	Il1rapl2	GABAergic	Global 23
9	Whrn	Glutamatergic	Global 0	Grm1	GABAergic	Global 1	Grm1	GABAergic	Global 2	Grm1	GABAergic	Global 5	Sln	GABAergic	Global 10	Fam212b	GABAergic	Global 23
10	Tmie	Glutamatergic	Global 0	Whrn	Glutamatergic	Global 1	Whrn	Glutamatergic	Global 2	Whrn	Glutamatergic	Global 5	Penk	GABAergic	Global 10	Sln	GABAergic	Global 23
11	Lypd1	Glutamatergic	Global 0	Tmie	Glutamatergic	Global 1	Tmie	Glutamatergic	Global 2	Tmie	Glutamatergic	Global 5	Grm1	GABAergic	Global 10	Penk	GABAergic	Global 23
12	Inpp4b	Glutamatergic	Global 0	Lypd1	Glutamatergic	Global 1	Lypd1	Glutamatergic	Global 2	Lypd1	Glutamatergic	Global 5	Whrn	Glutamatergic	Global 10	Grm1	GABAergic	Global 23
13	Adssl1	Glutamatergic	Global 0	Inpp4b	Glutamatergic	Global 1	Inpp4b	Glutamatergic	Global 2	Inpp4b	Glutamatergic	Global 5	Tmie	Glutamatergic	Global 10	Whrn	Glutamatergic	Global 23
14	Cd24a	Glutamatergic	Global 0	Adssl1	Glutamatergic	Global 1	Adssl1	Glutamatergic	Global 2	Adssl1	Glutamatergic	Global 5	Lypd1	Glutamatergic	Global 10	Tmie	Glutamatergic	Global 23
15	Chrna6	Glutamatergic	Global 0	Cd24a	Glutamatergic	Global 1	Cd24a	Glutamatergic	Global 2	Cd24a	Glutamatergic	Global 5	Inpp4b	Glutamatergic	Global 10	Lypd1	Glutamatergic	Global 23
16	Man1a	Glutamatergic	Global 0	Chrna6	Glutamatergic	Global 1	Chrna6	Glutamatergic	Global 2	Chrna6	Glutamatergic	Global 5	Adssl1	Glutamatergic	Global 10	Inpp4b	Glutamatergic	Global 23
17	Tshz2	Glutamatergic	Global 0	Man1a	Glutamatergic	Global 1	Man1a	Glutamatergic	Global 2	Man1a	Glutamatergic	Global 5	Cd24a	Glutamatergic	Global 10	Adssl1	Glutamatergic	Global 23
18	Tdo2	Glutamatergic	Global 0	Tshz2	Glutamatergic	Global 1	Tshz2	Glutamatergic	Global 2	Tshz2	Glutamatergic	Global 5	Chrna6	Glutamatergic	Global 10	Cd24a	Glutamatergic	Global 23
19	Unc5d	Glutamatergic	Global 0	Tdo2	Glutamatergic	Global 1	Tdo2	Glutamatergic	Global 2	Tdo2	Glutamatergic	Global 5	Man1a	Glutamatergic	Global 10	Chrna6	Glutamatergic	Global 23
20	Lhx1	Glutamatergic	Global 0	Unc5d	Glutamatergic	Global 1	Unc5d	Glutamatergic	Global 2	Unc5d	Glutamatergic	Global 5	Tshz2	Glutamatergic	Global 10	Man1a	Glutamatergic	Global 23
21	Aqp4	Non-Neuronal	Global 0	Lhx1	Glutamatergic	Global 1	Defb1	Glutamatergic	Global 2	Defb1	Glutamatergic	Global 5	Tdo2	Glutamatergic	Global 10	Tshz2	Glutamatergic	Global 23
22	Sdc3	Non-Neuronal	Global 0	Aqp4	Non-Neuronal	Global 1	Lhx1	Glutamatergic	Global 2	Lhx1	Glutamatergic	Global 5	Unc5d	Glutamatergic	Global 10	Tdo2	Glutamatergic	Global 23
23	Olfm2	Non-Neuronal	Global 0	Sdc3	Non-Neuronal	Global 1	Aqp4	Non-Neuronal	Global 2	Aqp4	Non-Neuronal	Global 5	Defb1	Glutamatergic	Global 10	Unc5d	Glutamatergic	Global 23
24	Siglech	Non-Neuronal	Global 0	Olfm2	Non-Neuronal	Global 1	Sdc3	Non-Neuronal	Global 2	Sdc3	Non-Neuronal	Global 5	Lhx1	Glutamatergic	Global 10	Defb1	Glutamatergic	Global 23
25	NaN	NaN	NaN	Siglech	Non-Neuronal	Global 1	Olfm2	Non-Neuronal	Global 2	Olfm2	Non-Neuronal	Global 5	Aqp4	Non-Neuronal	Global 10	Lhx1	Glutamatergic	Global 23
26	NaN	NaN	NaN	NaN	NaN	NaN	Siglech	Non-Neuronal	Global 2	Siglech	Non-Neuronal	Global 5	Sdc3	Non-Neuronal	Global 10	Aqp4	Non-Neuronal	Global 23
27	NaN	NaN	NaN	NaN	NaN	NaN	NaN	NaN	NaN	NaN	NaN	NaN	Olfm2	Non-Neuronal	Global 10	Sdc3	Non-Neuronal	Global 23
28	NaN	NaN	NaN	NaN	NaN	NaN	NaN	NaN	NaN	NaN	NaN	NaN	Siglech	Non-Neuronal	Global 10	Olfm2	Non-Neuronal	Global 23
29	NaN	NaN	NaN	NaN	NaN	NaN	NaN	NaN	NaN	NaN	NaN	NaN	NaN	NaN	NaN	Siglech	Non-Neuronal	Global 23

Table S4: Areas exhibiting high variance at components of interest. a) components 0, 2, 4, 5, 7 and 8 of the vis injection group. b) components 0 and 4 of the cp injection group and components 0, 3 and 5 of the cp injection group. c) components 0, 1, 2, 5, 10 and 23 from the the global analysis. Areas with more than 40 highlighted voxels have been selected.

(a)

	Visual group 0	Visual group 2	Visual group 4	Visual group 5	Visual group 7	Visual group 8
0	Ammon's horn	Cortical plate	Ansiform lobule	Cortical plate	Ammon's horn	Agranular insular area, dorsal part
1	Cortical plate	Hindbrain	Culmen	Dentate gyrus	Dentate gyrus	Ammon's horn
2	Dentate gyrus	Hypothalamic lateral zone	Hemispheric regions	Entorhinal area, lateral part	Primary somatosensory area, barrel field	Cortical amygdalar area, posterior part
3	Hemispheric regions	Hypothalamic medial zone	Striatum dorsal region	Hemispheric regions	Primary somatosensory area, mouth	Dentate gyrus
4	Hindbrain	Medulla, motor related	Striatum ventral region	Hindbrain	Primary somatosensory area, nose	Entorhinal area, lateral part
5	Lateral visual area	Medulla, sensory related	Vermal regions	Olfactory areas	Primary somatosensory area, upper limb	Entorhinal area, medial part, dorsal zone
6	Olfactory areas	Midbrain, motor related	cerebellum related fiber tracts	Primary visual area	Primary visual area	Olfactory areas
7	Postrhinal area	Olfactory areas	None	Retrosplenial area, dorsal part	Striatum dorsal region	Postrhinal area
8	Primary visual area	Pons, motor related	None	Striatum dorsal region	Supplemental somatosensory area	Primary motor area
9	Retrosplenial area, dorsal part	Pons, sensory related	None	Striatum ventral region	fornix system	Primary visual area
10	Retrosplenial area, lateral agranular part	Pretectal region	None	olfactory nerve	ventricular systems	Retrosplenial area, lateral agranular part
11	Secondary motor area	Primary visual area	None	None	None	Secondary motor area
12	Temporal association areas	Striatum dorsal region	None	None	None	Striatum dorsal region
13	cerebellar peduncles	Striatum ventral region	None	None	None	Temporal association areas
14	olfactory nerve	Superior colliculus, motor related	None	None	None	corpus callosum
15	posteromedial visual area	Vestibular nuclei	None	None	None	fornix system
16	None	olfactory nerve	None	None	None	posteromedial visual area
17	None	None	None	None	None	thalamus related
18	None	None	None	None	None	ventricular systems

(b)

	Caudoputamen group 0	Caudoputamen group 3	Caudoputamen group 5	Midbrain group 0	Midbrain group 4
0	Agranular insular area, dorsal part	Cortical plate	Ansiform lobule	Ammon's horn	Ansiform lobule
1	Agranular insular area, posterior part	Hindbrain	Central amygdalar nucleus	Anterior area	Brain stem
2	Ammon's horn	Hypothalamic lateral zone	Culmen	Anterior cingulate area, dorsal part	Cortical plate
3	Anterior cingulate area, dorsal part	Hypothalamic medial zone	Hemispheric regions	Cortical plate	Culmen
4	Cortical plate	Medulla, motor related	Hypothalamic lateral zone	Dentate gyrus	Hemispheric regions
5	Dentate gyrus	Medulla, sensory related	Hypothalamic medial zone	Hemispheric regions	Hindbrain
6	Gustatory areas	Midbrain, motor related	Interbrain	Hindbrain	Medulla, motor related
7	Hemispheric regions	Olfactory areas	Midbrain, motor related	Midbrain, motor related	Medulla, sensory related
8	Hindbrain	Pons, motor related	Pallidum, caudal region	Olfactory areas	Midbrain, motor related
9	Olfactory areas	Pons, sensory related	Periventricular region	Prelimbic area	Olfactory areas
10	Primary motor area	Primary visual area	Periventricular zone	Primary motor area	Pons, motor related
11	Primary somatosensory area, mouth	Striatum dorsal region	Striatum dorsal region	Primary somatosensory area, lower limb	Striatum dorsal region
12	Retrosplenial area, lateral agranular part	Striatum ventral region	Striatum ventral region	Primary somatosensory area, trunk	Striatum ventral region
13	Secondary motor area	Vestibular nuclei	Striatum-like amygdalar nuclei	Primary visual area	Superior colliculus, motor related
14	Striatum dorsal region	olfactory nerve	Vermal regions	Retrosplenial area, lateral agranular part	Vermal regions
15	Supplemental somatosensory area	None	cerebellum related fiber tracts	Secondary motor area	Vestibular nuclei
16	Visceral area	None	corticospinal tract	Superior colliculus, motor related	cerebellum related fiber tracts
17	olfactory nerve	None	None	Temporal association areas	olfactory nerve
18	trigeminal nerve	None	None	olfactory nerve	None

(c)

	Global 0	Global 1	Global 2	Global 5	Global 10	Global 23
0	Ammon's horn	Agranular insular area, dorsal part	Agranular insular area, posterior part	Ammon's horn	Agranular insular area, dorsal part	Ammon's horn
1	Anterior area	Brain stem	Ammon's horn	Ansiform lobule	Agranular insular area, ventral part	Dentate gyrus
2	Anterior cingulate area, dorsal part	Hypothalamic lateral zone	Ansiform lobule	Culmen	Ammon's horn	Dorsal auditory area
3	Cortical plate	Hypothalamic medial zone	Basolateral amygdalar nucleus	Hemispheric regions	Cortical amygdalar area, posterior part	Ectorhinal area
4	Dentate gyrus	Interbrain	Basomedial amygdalar nucleus	Lateral visual area	Dentate gyrus	Entorhinal area, lateral part
5	Hemispheric regions	Lateral septal nucleus	Central amygdalar nucleus	Olfactory areas	Entorhinal area, lateral part	Intralaminar nuclei of the dorsal thalamus
6	Olfactory areas	Midbrain, motor related	Cerebral nuclei	Primary visual area	Entorhinal area, medial part, dorsal zone	Medial group of the dorsal thalamus
7	Prelimbic area	Midline group of the dorsal thalamus	Culmen	Vermal regions	Frontal pole, cerebral cortex	Midbrain, motor related
8	Primary motor area	Olfactory areas	Endopiriform nucleus	cerebellum related fiber tracts	Olfactory areas	Olfactory areas
9	Primary somatosensory area, lower limb	Pallidum, caudal region	Hemispheric regions	fornix system	Orbital area, lateral part	Primary auditory area
10	Primary visual area	Periventricular region	Lateral visual area	None	Orbital area, medial part	Primary somatosensory area, mouth
11	Retrosplenial area, lateral agranular part	Primary motor area	Medulla, motor related	None	Orbital area, ventrolateral part	Primary somatosensory area, nose
12	Secondary motor area	Primary somatosensory area, mouth	Primary visual area	None	Prelimbic area	Retrohippocampal region
13	Temporal association areas	Secondary motor area	Striatum dorsal region	None	Primary visual area	Striatum dorsal region
14	olfactory nerve	Striatum dorsal region	Striatum ventral region	None	Secondary motor area	Striatum-like amygdalar nuclei
15	None	Striatum-like amygdalar nuclei	Striatum-like amygdalar nuclei	None	Temporal association areas	Superior colliculus, motor related
16	None	None	Vermal regions	None	None	Supplemental somatosensory area
17	None	None	Vestibular nuclei	None	None	Temporal association areas
18	None	None	cerebellum related fiber tracts	None	None	Ventral auditory area
19	None	None	None	None	None	Ventral group of the dorsal thalamus

Table S5: Areas exhibiting high variance at the tract-tracing maps. a) vis group. b) cp and mrn groups. Areas with more than 40 highlighted voxels have been selected.

(a)

	Visual group 0	Visual group 2	Visual group 4	Visual group 5	Visual group 7	Visual group 8
0	Agranular insular area, dorsal part	Anterior cingulate area, dorsal part	Ammon's horn	Ammon's horn	Ammon's horn	Ammon's horn
1	Ammon's horn	Anterior group of the dorsal thalamus	Anterior area	Anterior area	Ansiform lobule	Anterior area
2	Ansiform lobule	Anterolateral visual area	Anterior cingulate area, dorsal part	Brain stem	Anterior group of the dorsal thalamus	Anterior group of the dorsal thalamus
3	Anterior group of the dorsal thalamus	Brain stem	Anterolateral visual area	Dorsal auditory area	Anteromedial visual area	Anteromedial visual area
4	Anterolateral visual area	Dorsal part of the lateral geniculate complex	Entorhinal area, lateral part	Inferior colliculus	Hemispheric regions	Lateral group of the dorsal thalamus
5	Brain stem	Entorhinal area, lateral part	Lateral group of the dorsal thalamus	Lateral group of the dorsal thalamus	Lateral group of the dorsal thalamus	Lateral septal nucleus
6	Central lobule	Lateral group of the dorsal thalamus	Lateral visual area	Lateral visual area	Lateral visual area	Lateral visual area
7	Cerebellar nuclei	Lateral visual area	Posterolateral visual area	Midbrain, motor related	Olfactory areas	Posterolateral visual area
8	Cortical plate	Posterolateral visual area	Postrhinal area	Postrhinal area	Postrhinal area	Pretectal region
9	Culmen	Postrhinal area	Pretectal region	Pretectal region	Primary somatosensory area, barrel field	Primary visual area
10	Dentate gyrus	Pretectal region	Primary somatosensory area, barrel field	Primary auditory area	Primary visual area	Retrohippocampal region
11	Dorsal part of the lateral geniculate complex	Primary auditory area	Primary visual area	Primary somatosensory area, trunk	Retrohippocampal region	Retrosplenial area, dorsal part
12	Geniculate group, ventral thalamus	Primary visual area	Retrohippocampal region	Primary visual area	Retrosplenial area, dorsal part	Retrosplenial area, lateral agranular part
13	Gustatory areas	Retrosplenial area, dorsal part	Retrosplenial area, dorsal part	Retrohippocampal region	Retrosplenial area, lateral agranular part	Retrosplenial area, ventral part
14	Hemispheric regions	Retrosplenial area, lateral agranular part	Retrosplenial area, lateral agranular part	Retrosplenial area, dorsal part	Retrosplenial area, ventral part	Rostrolateral visual area
15	Hindbrain	Retrosplenial area, ventral part	Retrosplenial area, ventral part	Retrosplenial area, lateral agranular part	Striatum dorsal region	Striatum dorsal region
16	Hypothalamic lateral zone	Rostrolateral visual area	Rostrolateral visual area	Retrosplenial area, ventral part	Striatum ventral region	Striatum ventral region
17	Inferior colliculus	Striatum dorsal region	Striatum dorsal region	Rostrolateral visual area	Superior colliculus, sensory related	Superior colliculus, motor related
18	Lateral group of the dorsal thalamus	Superior colliculus, motor related	Striatum ventral region	Striatum dorsal region	Temporal association areas	Superior colliculus, sensory related
19	Lateral visual area	Superior colliculus, sensory related	Superior colliculus, motor related	Striatum ventral region	corpus callosum	Temporal association areas
20	Medulla, motor related	Temporal association areas	Superior colliculus, sensory related	Superior colliculus, motor related	fornix system	corpus callosum
21	Medulla, sensory related	Ventral auditory area	Temporal association areas	Superior colliculus, sensory related	posteromedial visual area	fornix system
22	Midbrain, motor related	corpus callosum	corpus callosum	Supplemental somatosensory area	thalamus related	posteromedial visual area
23	Olfactory areas	posteromedial visual area	posteromedial visual area	Temporal association areas	None	thalamus related
24	Pons, motor related	thalamus related	thalamus related	corpus callosum	None	None
25	Pons, sensory related	None	None	corticospinal tract	None	None
26	Postrhinal area	None	None	thalamus related	None	None
27	Pretectal region	None	None	None	None	None
28	Primary motor area	None	None	None	None	None
29	Primary somatosensory area, barrel field	None	None	None	None	None
30	Primary somatosensory area, mouth	None	None	None	None	None
31	Primary somatosensory area, nose	None	None	None	None	None
32	Primary somatosensory area, upper limb	None	None	None	None	None
33	Primary visual area	None	None	None	None	None
34	Retrosplenial area, dorsal part	None	None	None	None	None
35	Retrosplenial area, lateral agranular part	None	None	None	None	None
36	Secondary motor area	None	None	None	None	None
37	Striatum dorsal region	None	None	None	None	None
38	Striatum ventral region	None	None	None	None	None
39	Striatum-like amygdalar nuclei	None	None	None	None	None
40	Superior colliculus, motor related	None	None	None	None	None
41	Superior colliculus, sensory related	None	None	None	None	None
42	Supplemental somatosensory area	None	None	None	None	None
43	Thalamus, polymodal association cortex related	None	None	None	None	None
44	Ventral posterior complex of the thalamus	None	None	None	None	None
45	Vermal regions	None	None	None	None	None
46	Vestibular nuclei	None	None	None	None	None
47	Visceral area	None	None	None	None	None
48	cerebellum related fiber tracts	None	None	None	None	None
49	corpus callosum	None	None	None	None	None
50	posteromedial visual area	None	None	None	None	None
51	thalamus related	None	None	None	None	None

(b)

	Caudoputamen group 0	Caudoputamen group 3	Caudoputamen group 5	Midbrain group 0	Midbrain group 4
0	Agranular insular area, dorsal part	Agranular insular area, dorsal part	Agranular insular area, dorsal part	Agranular insular area, dorsal part	Brain stem
1	Agranular insular area, posterior part	Agranular insular area, posterior part	Agranular insular area, posterior part	Agranular insular area, posterior part	Hindbrain
2	Anterior cingulate area, ventral part	Cerebral nuclei	Brain stem	Agranular insular area, ventral part	Hypothalamic lateral zone
3	Brain stem	Cortical subplate	Cerebral nuclei	Ammon's horn	Interbrain
4	Gustatory areas	Gustatory areas	Cortical subplate	Ansiform lobule	Intralaminar nuclei of the dorsal thalamus
5	Hindbrain	Olfactory areas	Ectorhinal area	Anterior area	Medulla, motor related
6	Midbrain, motor related	Pallidum, dorsal region	Endopiriform nucleus	Anterior cingulate area, dorsal part	Midbrain, motor related
7	Olfactory areas	Primary motor area	Gustatory areas	Anterior cingulate area, ventral part	Pons, behavioral state related
8	Pallidum, dorsal region	Primary somatosensory area, mouth	Medulla, sensory related	Anterolateral visual area	Pons, motor related
9	Primary motor area	Primary somatosensory area, upper limb	Midbrain, motor related	Brain stem	Pretectal region
10	Primary somatosensory area, mouth	Secondary motor area	Olfactory areas	Central lobule	Primary visual area
11	Secondary motor area	Striatum dorsal region	Pallidum, dorsal region	Cochlear nuclei	Retrosplenial area, dorsal part
12	Striatum dorsal region	Supplemental somatosensory area	Primary motor area	Cortical amygdalar area, posterior part	Retrosplenial area, lateral agranular part
13	Supplemental somatosensory area	Temporal association areas	Primary somatosensory area, mouth	Cortical plate	Striatum dorsal region
14	Thalamus, polymodal association cortex related	Visceral area	Secondary motor area	Culmen	Superior colliculus, motor related
15	Ventral group of the dorsal thalamus	corpus callosum	Striatum dorsal region	Dentate gyrus	corpus callosum
16	Visceral area	corticospinal tract	Supplemental somatosensory area	Dorsal auditory area	posteromedial visual area
17	corpus callosum	None	Temporal association areas	Ectorhinal area	None
18	corticospinal tract	None	Visceral area	Entorhinal area, lateral part	None
19	None	None	corpus callosum	Entorhinal area, medial part, dorsal zone	None
20	None	None	corticospinal tract	Frontal pole, cerebral cortex	None
21	None	None	None	Gustatory areas	None
22	None	None	None	Hemispheric regions	None
23	None	None	None	Hypothalamic lateral zone	None
24	None	None	None	Interbrain	None
25	None	None	None	Lateral visual area	None
26	None	None	None	Midbrain, motor related	None
27	None	None	None	Olfactory areas	None
28	None	None	None	Orbital area, lateral part	None
29	None	None	None	Orbital area, medial part	None
30	None	None	None	Orbital area, ventrolateral part	None
31	None	None	None	Pons, behavioral state related	None
32	None	None	None	Prelimbic area	None
33	None	None	None	Pretectal region	None
34	None	None	None	Primary auditory area	None
35	None	None	None	Primary motor area	None
36	None	None	None	Primary somatosensory area, barrel field	None
37	None	None	None	Primary somatosensory area, lower limb	None
38	None	None	None	Primary somatosensory area, mouth	None
39	None	None	None	Primary somatosensory area, nose	None
40	None	None	None	Primary somatosensory area, trunk	None
41	None	None	None	Primary somatosensory area, unassigned	None
42	None	None	None	Primary somatosensory area, upper limb	None
43	None	None	None	Primary visual area	None
44	None	None	None	Retrohippocampal region	None
45	None	None	None	Retrosplenial area, dorsal part	None
46	None	None	None	Retrosplenial area, ventral part	None
47	None	None	None	Rostrolateral visual area	None
48	None	None	None	Secondary motor area	None
49	None	None	None	Striatum dorsal region	None
50	None	None	None	Superior colliculus, motor related	None
51	None	None	None	Supplemental somatosensory area	None
52	None	None	None	Temporal association areas	None
53	None	None	None	Ventral auditory area	None
54	None	None	None	Ventral posterior complex of the thalamus	None
55	None	None	None	Vermal regions	None
56	None	None	None	Visceral area	None
57	None	None	None	cerebellar peduncles	None
58	None	None	None	cerebellum related fiber tracts	None
59	None	None	None	corpus callosum	None
60	None	None	None	fornix system	None
61	None	None	None	olfactory nerve	None
62	None	None	None	thalamus related	None
63	None	None	None	ventricular systems	None

Table S6: Areas exhibiting high variance at the tract-tracing maps that were formed by components 0, 1, 2, 5, 10 and 23 of the global analysis, comprised of projections from multiple cortical areas. Areas with more than 40 highlighted voxels have been selected.

	Global 0	Global 1	Global 2	Global 5	Global 10	Global 23
0	Agranular insular area, dorsal part	Agranular insular area, dorsal part	Agranular insular area, dorsal part	Agranular insular area, posterior part	Agranular insular area, dorsal part	Ammon's horn
1	Agranular insular area, posterior part	Basomedial amygdalar nucleus	Agranular insular area, posterior part	Ammon's horn	Agranular insular area, ventral part	Basomedial amygdalar nucleus
2	Basolateral amygdalar nucleus	Cerebral nuclei	Basolateral amygdalar nucleus	Basolateral amygdalar nucleus	Anterior cingulate area, dorsal part	Brain stem
3	Cortical subplate	Hypothalamic lateral zone	Basomedial amygdalar nucleus	Basomedial amygdalar nucleus	Cortical plate	Central amygdalar nucleus
4	Ectorhinal area	Hypothalamic medial zone	Central amygdalar nucleus	Central amygdalar nucleus	Lateral group of the dorsal thalamus	Dentate gyrus
5	Entorhinal area, lateral part	Interbrain	Cerebral nuclei	Cerebral nuclei	Medial group of the dorsal thalamus	Dorsal auditory area
6	Hindbrain	Lateral septal nucleus	Cortical subplate	Dentate gyrus	Olfactory areas	Lateral septal nucleus
7	Hypothalamic medial zone	Midbrain, motor related	Dorsal part of the lateral geniculate complex	Lateral septal nucleus	Orbital area, lateral part	Midbrain, motor related
8	Interbrain	Midline group of the dorsal thalamus	Endopiriform nucleus	Lateral visual area	Orbital area, medial part	Pallidum, ventral region
9	Lateral visual area	Pallidum, caudal region	Entorhinal area, lateral part	Medial septal complex	Orbital area, ventrolateral part	Primary auditory area
10	Medulla, motor related	Periventricular region	Hindbrain	Primary visual area	Prelimbic area	Primary motor area
11	Medulla, sensory related	Primary motor area	Lateral visual area	Striatum dorsal region	Primary motor area	Retrohippocampal region
12	Medullary reticular nucleus	Primary somatosensory area, mouth	Medulla, motor related	Striatum ventral region	Primary somatosensory area, lower limb	Secondary motor area
13	Midbrain, motor related	Secondary motor area	Medullary reticular nucleus	Striatum-like amygdalar nuclei	Primary somatosensory area, mouth	Striatum dorsal region
14	Paragigantocellular reticular nucleus	Striatum dorsal region	Olfactory areas	corpus callosum	Primary somatosensory area, upper limb	Striatum ventral region
15	Periventricular region	Striatum-like amygdalar nuclei	Paragigantocellular reticular nucleus	fornix system	Secondary motor area	Striatum-like amygdalar nuclei
16	Pons, behavioral state related	Supplemental somatosensory area	Primary visual area	None	Striatum dorsal region	Superior colliculus, motor related
17	Pons, motor related	None	Striatum dorsal region	None	Striatum ventral region	Supplemental somatosensory area
18	Primary visual area	None	Striatum ventral region	None	Supplemental somatosensory area	corpus callosum
19	Striatum dorsal region	None	Striatum-like amygdalar nuclei	None	Ventral posterior complex of the thalamus	None
20	Striatum ventral region	None	Superior colliculus, sensory related	None	corticospinal tract	None
21	Supplemental somatosensory area	None	corpus callosum	None	None	None
22	Visceral area	None	thalamus related	None	None	None

Table S7: For components of interest a number of areas have been highlighted by the color-coded maps with high variance in both the modality-specific and the shared spatial maps (labeled as bimodal), with high variance in both the gene and the shared spatial maps (labeled as gene expression driven) and with high variance in both the projection and the shared spatial maps (labeled as projection density driven). a) vis injection group. b) mrn and cp injection groups. c) global components. Areas with more than 40 highlighted voxels have been selected.

(a)

	Visual group 0	Visual group 2	Visual group 4	Visual group 5	Visual group 7	Visual group 8
bimodal	Olfactory areas	Primary visual area	None	None	None	Primary visual area
gene expression driven	Ammon’s horn	Cortical plate	Ansiform lobule	Cortical plate	Ammon’s horn	Agranular insular area, dorsal part
gene expression driven	Dentate gyrus	Hindbrain	Culmen	Dentate gyrus	Dentate gyrus	Ammon’s horn
gene expression driven	Hemispheric regions	Hypothalamic medial zone	Hemispheric regions	Hemispheric regions	Primary somatosensory area, barrel field	Cortical amygdalar area, posterior part
gene expression driven	Hindbrain	Medulla, motor related	Striatum dorsal region	Hindbrain	Primary somatosensory area, mouth	Dentate gyrus
gene expression driven	Retrosplenial area, lateral agranular part	Medulla, sensory related	Striatum ventral region	Olfactory areas	Primary somatosensory area, nose	Entorhinal area, lateral part
gene expression driven	Secondary motor area	Midbrain, motor related	Vermal regions	Striatum dorsal region	Primary somatosensory area, upper limb	Entorhinal area, medial part, dorsal zone
gene expression driven	cerebellar peduncles	Olfactory areas	cerebellum related fiber tracts	Striatum ventral region	Striatum dorsal region	Olfactory areas
gene expression driven	olfactory nerve	Pons, motor related	None	olfactory nerve	Supplemental somatosensory area	Postrhinal area
gene expression driven	None	Pons, sensory related	None	None	fornix system	Primary motor area
gene expression driven	None	Striatum dorsal region	None	None	ventricular systems	Secondary motor area
gene expression driven	None	Striatum ventral region	None	None	None	Temporal association areas
gene expression driven	None	Superior colliculus, motor related	None	None	None	fornix system
gene expression driven	None	Vestibular nuclei	None	None	None	None
gene expression driven	None	olfactory nerve	None	None	None	None
projection density driven	Lateral visual area	None	None	None	None	None
projection density driven	Primary visual area	None	None	None	None	None

(b)

	Caudoputamen group 0	Caudoputamen group 3	Caudoputamen group 5	Midbrain group 0	Midbrain group 4
bimodal	None	None	None	Ammon’s horn	None
bimodal	None	None	None	Cortical plate	None
bimodal	None	None	None	Dentate gyrus	None
bimodal	None	None	None	Olfactory areas	None
bimodal	None	None	None	Primary motor area	None
bimodal	None	None	None	Secondary motor area	None
gene expression driven	Ammon’s horn	Cortical plate	Ansiform lobule	Hemispheric regions	Ansiform lobule
gene expression driven	Anterior cingulate area, dorsal part	Hindbrain	Culmen	Hindbrain	Cortical plate
gene expression driven	Cortical plate	Hypothalamic lateral zone	Hemispheric regions	Primary visual area	Culmen
gene expression driven	Dentate gyrus	Hypothalamic medial zone	Hypothalamic lateral zone	Retrosplenial area, lateral agranular part	Hemispheric regions
gene expression driven	Hemispheric regions	Medulla, motor related	Hypothalamic medial zone	olfactory nerve	Hindbrain
gene expression driven	Hindbrain	Medulla, sensory related	Interbrain	None	Medulla, motor related
gene expression driven	Olfactory areas	Midbrain, motor related	Periventricular region	None	Medulla, sensory related
gene expression driven	Secondary motor area	Olfactory areas	Vermal regions	None	Olfactory areas
gene expression driven	olfactory nerve	Pons, motor related	cerebellum related fiber tracts	None	Pons, motor related
gene expression driven	None	Pons, sensory related	None	None	Striatum ventral region
gene expression driven	None	Striatum ventral region	None	None	Vermal regions
gene expression driven	None	Vestibular nuclei	None	None	Vestibular nuclei
gene expression driven	None	olfactory nerve	None	None	cerebellum related fiber tracts
gene expression driven	None	None	None	None	olfactory nerve
projection density driven	Agranular insular area, dorsal part	None	Midbrain, motor related	Midbrain, motor related	Midbrain, motor related
projection density driven	Agranular insular area, posterior part	None	Striatum dorsal region	Superior colliculus, motor related	Striatum dorsal region
projection density driven	Gustatory areas	None	None	None	Superior colliculus, motor related
projection density driven	Primary motor area	None	None	None	None
projection density driven	Primary somatosensory area, mouth	None	None	None	None
projection density driven	Striatum dorsal region	None	None	None	None
projection density driven	Supplemental somatosensory area	None	None	None	None
projection density driven	Visceral area	None	None	None	None

(c)

	Global 0	Global 1	Global 2	Global 5	Global 10	Global 23
bimodal	None	Hypothalamic medial zone	None	None	None	None
bimodal	None	Periventricular region	None	None	None	None
gene expression driven	Ammon’s horn	Olfactory areas	Ammon’s horn	Ansiform lobule	Agranular insular area, dorsal part	Dentate gyrus
gene expression driven	Anterior area	None	Ansiform lobule	Culmen	Cortical amygdalar area, posterior part	Entorhinal area, lateral part
gene expression driven	Anterior cingulate area, dorsal part	None	Culmen	Hemispheric regions	Dentate gyrus	Olfactory areas
gene expression driven	Cortical plate	None	Hemispheric regions	Olfactory areas	Entorhinal area, lateral part	Primary somatosensory area, mouth
gene expression driven	Dentate gyrus	None	Striatum dorsal region	Vermal regions	Entorhinal area, medial part, dorsal zone	Primary somatosensory area, nose
gene expression driven	Hemispheric regions	None	Vermal regions	cerebellum related fiber tracts	Frontal pole, cerebral cortex	Temporal association areas
gene expression driven	Olfactory areas	None	Vestibular nuclei	None	Olfactory areas	None
gene expression driven	Prelimbic area	None	cerebellum related fiber tracts	None	Prelimbic area	None
gene expression driven	Primary motor area	None	None	None	Primary visual area	None
gene expression driven	Primary visual area	None	None	None	Secondary motor area	None
gene expression driven	Retrosplenial area, lateral agranular part	None	None	None	Temporal association areas	None
gene expression driven	Secondary motor area	None	None	None	None	None
gene expression driven	Temporal association areas	None	None	None	None	None
gene expression driven	olfactory nerve	None	None	None	None	None
projection density driven	None	Hypothalamic lateral zone	Agranular insular area, posterior part	Ammon’s horn	Agranular insular area, ventral part	Ammon’s horn
projection density driven	None	Interbrain	Basolateral amygdalar nucleus	Lateral visual area	Orbital area, lateral part	Dorsal auditory area
projection density driven	None	Lateral septal nucleus	Basomedial amygdalar nucleus	Primary visual area	Orbital area, ventrolateral part	Midbrain, motor related
projection density driven	None	Midbrain, motor related	Central amygdalar nucleus	fornix system	None	Primary auditory area
projection density driven	None	Midline group of the dorsal thalamus	Cerebral nuclei	None	None	Striatum dorsal region
projection density driven	None	Pallidum, caudal region	Endopiriform nucleus	None	None	Striatum-like amygdalar nuclei
projection density driven	None	Primary motor area	Lateral visual area	None	None	Superior colliculus, motor related
projection density driven	None	Primary somatosensory area, mouth	Medulla, motor related	None	None	Supplemental somatosensory area
projection density driven	None	Secondary motor area	Primary visual area	None	None	None
projection density driven	None	Striatum dorsal region	Striatum ventral region	None	None	None
projection density driven	None	Striatum-like amygdalar nuclei	Striatum-like amygdalar nuclei	None	None	None

Table S8: Tables containing a number of areas with high variance in the MO group spatial maps. (a-e) ICAs 1, 2, 3, 8, 9 in the order of reference.

(a)

MO 1	voxel frequency per area
Striatum dorsal region	372
Secondary motor area	136
Ammon's horn	128
Dentate gyrus	74
Primary motor area	51
Vestibular nuclei	27
Pons, motor related	18
Midbrain, motor related	17
Lateral group of the dorsal thalamus	14
Hypothalamic lateral zone	14
Medulla, sensory related	14
Dorsal part of the lateral geniculate complex	11

(b) .

MO 2	voxel frequency per area
Secondary motor area	326
Striatum dorsal region	285
Cortical plate	113
olfactory nerve	87
Olfactory areas	64
Anterior cingulate area, dorsal part	49
Primary somatosensory area, barrel field	27
Primary motor area	24
Temporal association areas	12

(c) .

MO 3	voxel frequency per area
Secondary motor area	357
Primary motor area	316
Striatum dorsal region	286
Primary somatosensory area, upper limb	13
Supplemental somatosensory area	11
Primary somatosensory area, barrel field	11

(d) .

MO 8	voxel frequency per area
Striatum dorsal region	343
Secondary motor area	330
Primary motor area	94
Supplemental somatosensory area	25
Midbrain, motor related	16
Olfactory areas	16
corticospinal tract	16
Ventral group of the dorsal thalamus	15
corpus callosum	14
Primary somatosensory area, barrel field	14
corpus callosum, anterior forceps	11

(e) .

MO 9	voxel frequency per area
Striatum dorsal region	314
Secondary motor area	207
Primary motor area	172
Primary somatosensory area, barrel field	37
corticospinal tract	32
Visceral area	29
Supplemental somatosensory area	25
Primary somatosensory area, nose	20
Temporal association areas	17
Ectorhinal area	15
Primary somatosensory area, upper limb	15
Olfactory areas	13
Primary somatosensory area, lower limb	12
corpus callosum	12
Pons, motor related	12

Table S9: Tables containing correlation statistics between the *MO* group analysis and two additional analyses also performed in projection patterns from the motor cortex, specifically: factorisation using the gene expression and single-neuron data (a), and using the projection density and single-neuron data (b). The correlations were estimated using Pearson's rho, at the level of spatial maps (H) and coefficients (X).

(a) .

	gene-neuron ICA	spatial map rho	spatial map p	neuron coefficient rho	neuron coefficient p	gene coefficient rho	gene coefficient p
ICA 1	6	0.726	0.0	0.920	0.0	0.532	0.0
ICA 2	6	0.479	0.0	0.702	0.0	0.540	0.0
ICA 3	7	0.761	0.0	0.815	0.0	0.572	0.0
ICA 8	21	0.699	0.0	0.644	0.0	0.452	0.0
ICA 9	16	0.645	0.0	0.633	0.0	0.468	0.0

(b) .

	gene-injection ICA	spatial map rho	spatial map p	injection coefficient rho	injection coefficient p	gene coefficient rho	gene coefficient p
ICA 1	0	0.407	0.0	0.217	0.402	0.997	0.0
ICA 2	0	0.529	0.0	0.855	0.000	0.980	0.0
ICA 3	1	0.255	0.0	0.871	0.000	0.847	0.0
ICA 8	9	0.329	0.0	0.341	0.181	0.503	0.0
ICA 9	2	0.411	0.0	0.700	0.002	0.507	0.0

References

Bishop, C. M. (2006). *Pattern Recognition and Machine Learning*. Information Science and Statistics. Springer, first edition.

Cover, T. M. and Thomas, J. A. (1991). *Elements of Information Theory*. Handbook of Brain Connectivity. Wiley, 2 edition.

Daimon, C. M., Jasien, J. M., Wood, W. H., Zhang, Y., Becker, K. G., Silverman, J. L., Crawley, J. N., Martin, B., and Maudsley, S. (2015). Hippocampal transcriptomic and proteomic alterations in the btbr mouse model of autism spectrum disorder. *Frontiers in Physiology*, 6:324.

De la Rossa, A., Bellone, C., Golding, B., Vitali, I., Moss, J., Toni, N., Lüscher, C., and Jabaudon, D. (2013). In vivo reprogramming of circuit connectivity in postmitotic neocortical neurons. *Nature Neuroscience*, 16(2):193–200.

Economo, M. N., Clack, N. G., Lavis, L. D., Gerfen, C. R., Svoboda, K., Myers, E. W., and Chandrashekar, J. (2016). A platform for brain-wide imaging and reconstruction of individual neurons. *eLife*, 5:e10566.

French, L. and Pavlidis, P. (2011). Relationships between gene expression and brain wiring in the adult mouse brain. *PLoS Comput Biol*, 7:e1001049.

Friedman, J., Hastie, T., and Tibshirani, R. (2009). *The Elements of Statistical Learning. Data Mining, Inference, and Prediction*. Springer Series in Statistics. Springer, 2 edition.

Gold, D. L., Miecznikowski, J. C., and Liu, S. (2009). Error control variability in pathway-based microarray analysis. *Bioinformatics*, 25(17):2216–2221.

Goodman, J. V. and Bonni, A. (2019). Regulation of neuronal connectivity in the mammalian brain by chromatin remodeling. *Current Opinion in Neurobiology*, 59:59 – 68. Neural Epigenetics.

Groves, A. R., Beckmann, C. F., Smith, S. M., and Woolrich, M. W. (2011). Linked independent component analysis for multimodal data fusion. *NeuroImage*, 54(3):2198–2217.

Han, Y., Kebschull, J., Campbell, R., Cowan, D., Imhof, F., Zador, A. M., and Mrsic-Flogel, T. D. (2018). The logic of single-cell projections from visual cortex. *Nature*, 556(5):51–56.

Kohavi, R. (1995). A study of cross-validation and bootstrap for accuracy estimation and model selection. In *Proceedings of the 14th international joint conference on Artificial intelligence (IJCAI*, volume 2, pages 1137–

1143.

Lein, E. S. et al. (2007). Genome-wide atlas of gene expression in the adult mouse brain. *Nature*, 445:168–176.

Li, Y., Chen, H., Jiang, X., Li, X., Lv, J., Peng, H., Tsien, J., and Liu, T. (2017). Discover mouse gene coexpression landscapes using dictionary learning and sparse coding. *Brain Structure and Function*, 222(9):4253–4270.

Mairal, J., Bach, F., Ponce, J., and Sapiro, G. (2010). Online learning for matrix factorization and sparse coding. *Journal of Machine Learning Research*, 11:19–60.

Miller, J. A., Horvath, S., and Geschwind, D. H. (2010). Divergence of human and mouse brain transcriptome highlights alzheimer disease pathways. *Proceedings of the National Academy of Sciences*, 107(28):12698–12703.

Ogata, H., Goto, S., Sato, K., Fujibuchi, W., Bono, H., and Kanehisa, M. (1999). Kegg: Kyoto encyclopedia of genes and genomes. *Nucleic Acids Research*, 27(1):29–34.

Pearson, K. (1901). On lines and planes of closest fit to systems of points in space. *The London, Edinburgh, and Dublin Philosophical Magazine and Journal of Science*, 2(11):559–572.

Polleux, F. (2005). Genetic mechanisms specifying cortical connectivity: Let's makesome projections together. *Neuron*, 46(3):395 – 400.

Razoux, F., Russig, H., Mueggler, T., Baltes, C., Dikaiou, K., Rudin, M., and Mansuy, I. M. (2017). Transgenerational disruption of functional 5-ht1ar-induced connectivity in the adult mouse brain by traumatic stress in early life. *Molecular Psychiatry*, 22(4):519–526.

Rice, J. A. (2007). *Mathematical Statistics and Data Analysis*. Mathematics of Computation. Duxbury Press, 3 edition.

Rivals, I., Personnaz, L., Taing, L., and Potier, M. C. (2007). Enrichment or depletion of a go category within a class of genes: which test? *Bioinformatics*, 23(4):401–407.

Strehl, A. and Ghosh, J. (2002). Cluster ensembles - a knowledge reuse framework for combining multiple partitions. *Journal of Machine Learning Research*, 3:583–617.

Tasic, B. et al. (2018). Shared and distinct transcriptomic cell types across neocortical areas. *Nature*, 563(7729):72–78.

Tikhonov, A. N. and Arsenin, V. Y. (1977). *Solution of Ill-posed Problems*. Mathematics of Computation. Winston & Sons, 1 edition.

Varma, S. and Simon, R. (2006). Bias in error estimation when using cross-validation for model selection. *BMC Bioinformatics*, 7:91.

von Luxburg, U. (2007). A tutorial on spectral clustering. *Statistics and Computing*, 17(4):395–416.

Wang, Q., Ding, S.-L., Li, Y., Royall, J., Feng, D., Lesnar, P., Graddis, N., Naeemi, M., Facer, B., Ho, A., Dolbeare, T., Blanchard, B., Dee, N., Wakeman, W., Hirokawa, K. E., Szafer, A., Sunkin, S. M., Oh, S. W., Bernard, A., Phillips, J. W., Hawrylycz, M., Koch, C., Zeng, H., Harris, J. A., and Ng, L. (2020). The allen mouse brain common coordinate framework: A 3d reference atlas. *Cell*, 181(4):936 – 953.e20.

Winnubst, J., Bas, E., Ferreira, T. A., Wu, Z., Economo, M. N., Edson, P., Arthur, B. J., Bruns, C., Rokicki, K., Schauder, D., Olbris, D. J., Murphy, S. D., Ackerman, D. G., Arshadi, C., Baldwin, P., Blake, R., Elsayed, A., Hasan, M., Ramirez, D., Santos], B. D., Weldon, M., Zafar, A., Dudman, J. T., Gerfen, C. R., Hantman, A. W., Korff, W., Sternson, S. M., Spruston, N., Svoboda, K., and Chandrashekar, J. (2019). Reconstruction of 1,000 projection neurons reveals new cell types and organization of long-range connectivity in the mouse brain. *Cell*, 179(1):268 – 281.e13.
